# Phytochemistry, pharmacology, safety, and applications of *Buddleja globosa* Hope [Scrophulariaceae]: a systematic review from bioactive metabolites and mechanisms to translational perspectives

**DOI:** 10.3389/fphar.2026.1940132

**Published:** 2026-10-14

**Authors:** Gonzalo Fuentes-Barros, Sebastián Castro-Saavedra, Nicolas Montalva, Marco Mellado, Rolando Vernal, Marcela Hernández, Antonia Díaz-Valdés, Camila Zoppi, Javier Echeverría

**Affiliations:** 1 SAPHYCHEM, Santiago, Chile; 2 Programa de Doctorado en Políticas Públicas, Universidad Mayor, Santiago, Chile; 3 Departamento de Ciencias del Ambiente, Facultad de Química y Biología, Universidad de Santiago de Chile, Santiago, Chile; 4 Centro de Investigación en Sociedad y Salud, Facultad de Ciencias Sociales y Artes, Universidad Mayor, Santiago, Chile; 5 Dirección de Investigación, Universidad Bernardo O’Higgins, Santiago, Chile; 6 Laboratorio de Espectroscopía y Química Aplicada, Grupo de Investigación en Ciencias Biomédicas Aplicadas, Universidad Central de Chile, Santiago, Chile; 7 Laboratorio de Biología Periodontal, Facultad de Odontología, Universidad de Chile, Santiago, Chile; 8 Departamento de Odontología Conservadora, Facultad de Odontología, Universidad de Chile, Santiago, Chile; 9 Departamento de Patología y Medicina Oral, Facultad de Odontología, Universidad de Chile, Santiago, Chile; 10 Facultad de Ciencias Sociales y Artes, Psicología, Universidad Mayor, Santiago, Chile; 11 Centro de Gerociencia para la Salud Cerebral y el Metabolismo, Santiago, Chile

**Keywords:** Buddleja globosa, gastric ulcers, matico, medicinal plants, skin wound, wound-healing

## Abstract

**Background:**

*Buddleja globosa* Hope [Scrophulariaceae] is a shrub native to South America, known in Chile as *matico* or *pañil*. Its leaves are widely used in traditional medicine and have commercial applications, primarily as aqueous extracts administered orally or topically. Its use is supported by over a century of traditional practice, and it is one of the few native species recognized as a traditional herbal medicine in Chile based on long-standing use and emerging scientific evidence.

**Methodology:**

We retrieved bibliographic data on the traditional uses, ethnopharmacology, phytochemistry, and biological activities of *B. globosa* from Web of Science®, Scopus®, PubMed®, SciFinder®, University of Chile Repository® and grey literature. We conducted a systematic review in accordance with the PRISMA 2020 guidelines.

**Results and discussion:**

Fifty studies were included in the systematic review. Traditional uses have made *B. globosa* one of the most important medicinal plant species in Chile for both internal and topical applications, although the strength of evidence varies by indication. Its use in treating gastric ulcers and promoting skin wound healing is particularly noteworthy, with pharmacological activities reported in *in vitro* studies, murine models, and clinical trials. The species contains a remarkable diversity of bioactive metabolites (*n* = 119), including verbascoside, echinacoside, buddledin A, α-amyrin, β-amyrin, luteolin, and apigenin; however, its essential oil remains poorly characterized. Furthermore, the review identified important knowledge gaps, particularly in medicinal cultivation and silvicultural management, chemical standardization, metabolomic variability, toxicological and safety assessment, and preclinical and clinical validation. Addressing these gaps will be essential to support the development of standardized phytotherapeutic products based on *B. globosa*.

**Conclusion:**

*Buddleja globosa* represents a strategic medicinal resource due to its long history of traditional use, its widespread use in Chile, and the growing body of scientific evidence suggesting its biological properties, particularly in wound-healing applications. This review documents several bioactive metabolites, including verbascoside, amyrins, and flavonoids. In this context, future research should focus on strategies that promote knowledge transfer and on developing public policies to implement Good Manufacturing Practices, translating their use into evidence-based healthcare.

**Systematic Review Registration:**

https://www.crd.york.ac.uk/PROSPERO/view/CRD420261450024.

## 1 Introduction

*Buddleja globosa* Hope [Scrophulariaceae] is a plant species native to South America, distributed mainly in Argentina, Bolivia, Peru, and Chile. It is commonly known in Spanish as “*matico*” and, in the Mapuche language^1^, as “*palñin*” or “*pañil*” by the indigenous inhabitants of south-central Chile, and as “palguín” in Quechua^2^ (Houghton, 2003; Bustamante et al., 2014).

*Buddleja globosa* is an evergreen shrub, up to 5 m tall, with a slender, soft, and branching trunk; it commonly produces numerous suckers at its base, which continue to grow after pruning. Its leaves are large, elliptic-ovate to lanceolate, dark green, somewhat rough on the upper surface, and covered with dense yellowish-white down on the underside. The flowers are small, hermaphroditic, yellow to orange, and arranged in dense, globose heads (Vogel et al., 2011; Pastene-Navarrete and Torres-Vega, 2021). In Chile, *B. globosa* has a wide latitudinal distribution, extending from the Región de Valparaíso (32°02′S) to the Región de Los Lagos (44°14′S) (Pastene-Navarrete and Torres-Vega, 2021). Its altitudinal distribution varies with the geomorphological formation in which it occurs; in interior valleys, populations are predominantly found at low altitudes, whereas in the Coastal Range they are distributed between 500 and 2000 m a.s.l. (Pastene-Navarrete and Torres-Vega, 2021).

*Buddleja globosa* has been traditionally used for generations to treat wounds, stomach problems, and inflammation. In addition to its traditional use, *B. globosa* has attracted increasing scientific interest as a cultivable, standardizable medicinal plant, particularly for its phenylpropanoids and flavonoids, which exhibit antioxidant, anti-inflammatory, antimicrobial, and wound-healing activities (Pastene-Navarrete and Torres-Vega, 2021).

A recent focused systematic review by Becerra et al. (2026) identified at least 27 bioactive compounds in *B. globosa* and synthesized the available evidence on its pharmacological properties, with antioxidant and anti-inflammatory activities emerging as the most extensively investigated and pharmacologically relevant effects (Supplementary Table S1). Complementing this previous work, the present systematic review provides a broader assessment of the research landscape on *B. globosa*. In addition to updating evidence on its phytochemical composition and pharmacological activities, this review incorporates findings from clinical studies and specifically examines the biological and pharmacological evidence associated with verbascoside. Furthermore, it expands the scope of the analysis to include emerging applications of *B. globosa* in nanotechnology and green chemistry, the regulatory status of its medicinal use in Chile, propagation and silvicultural management, cultivation, perspectives and challenges related to the standardization of *B. globosa*, the influence of drying conditions on its active constituents, and future research perspectives. Together, these complementary dimensions provide an integrated view of the current state of knowledge, research development, and translational potential of this medicinal species.

Considering the historical and public health relevance of *B*. *globosa* in Chile, the following section describes the methodology used to identify, organize, and systematically analyze the available evidence on this species.

## 2 Methodology

### 2.1 Search strategy

A systematic literature review was conducted following the PRISMA 2020 (Preferred Reporting Items for Systematic Reviews and Meta-Analyses) guidelines. We conducted bibliographic searches in Web of Science Core Collection®, Scopus®, PubMed®, SciFinder®, Academic Repository of the University of Chile and grey literature. Selected for their complementary coverage of phytochemistry, natural products chemistry, pharmacology, biomedical sciences, and Chilean scientific literature. We applied no language restrictions and included studies published in English and Spanish. Additionally, we performed manual searches in the reference lists of key articles, local Chilean journals not indexed in the aforementioned databases, and relevant theses and conference abstracts to identify additional studies, thereby capturing grey literature not readily retrievable through conventional indexing. We also conducted patent searches in the World Intellectual Property Organization (WIPO) database to identify technological developments related to the species; however, we did not include patent documents in the systematic review. The search included all peer-reviewed publications available up to July 2026. Similar search strategies have been adopted in recent systematic reviews on medicinal plants and phytotherapy, combining multiple bibliographic databases to maximize the retrieval of experimental evidence (Calzetta et al., 2020; Sánchez et al., 2020; Tafazoli and Tafazoli Moghadam, 2020; Annaz et al., 2022). The search strategy was developed in two complementary phases. In the first phase, broad searches were conducted using only the scientific and common names of *B. globosa* (“Buddleja globosa” OR “matico”) to maximize sensitivity and retrieve the largest possible number of potentially relevant records. In the second phase, these species terms were combined with thematic descriptors related to the scope of the review, including “phytochemistry,” “ethnomedicinal use,” and “pharmacological activity,” using Boolean operators (AND/OR) to focus the search on studies addressing chemical composition, traditional use, and pharmacological evaluation.

### 2.2 Eligibility criteria

Original studies investigating *B. globosa* were considered eligible when they provided experimental evidence on its phytochemistry, biological activity, medicinal cultivation, chemical characterization, or pharmacological properties, either as the sole study species or as part of comparative studies involving multiple plant species. Peer-reviewed original research articles constituted the primary source of evidence. In addition, we included a limited number of non-peer-reviewed sources when they contained original experimental data unavailable elsewhere and were considered scientifically relevant. These included a doctoral dissertation from the University of Chile and a conference contribution that reported UHPLC–MS-based phytochemical characterization and biological evaluations of *B. globosa*, compared with other native Chilean species. We included them because they contained original experimental evidence that had not subsequently been published in peer-reviewed journals (Godin et al., 2015; Scherer and Saldanha, 2019; Amanpour et al., 2023). Publications lacking original experimental data, duplicate records, editorials, narrative reviews, opinion articles, book chapters, and studies unrelated to this review’s objectives were excluded.

### 2.3 Identification and removal of duplicate records

The database searches identified 285 records, distributed among the six information sources as follows: Web of Science (*n* = 60), Scopus (*n* = 72), PubMed (*n* = 25), SciFinder (*n* = 52), Academic Repository of the University of Chile (*n* = 75), and conference abstracts (*n* = 1). An additional record was identified through a manual search of conference abstracts. All retrieved records were integrated into a single bibliographic database. Duplicate publications were identified manually using the article title as the primary matching criterion and, when necessary, verified by comparing authors, publication year, and journal information to resolve minor differences in bibliographic formatting among databases. Each duplicated publication was consolidated into a single record while preserving information about the databases from which it was retrieved, thereby documenting overlap among the different information sources. After duplicate removal, 175 records remained for screening.

### 2.4 Study selection

The systematic review protocol was retrospectively registered with PROSPERO (CRD420261450024) on 14 July 2026. We initiated systematic database searches on 14 June 2026 and continued through July 2026. We organized the eligibility criteria according to the PECO framework. Population (P) included studies evaluating *B*. *globosa* in experimental, observational, ethnobotanical, or preclinical contexts. Exposure (E) referred to the use, presence, or administration of *B*. *globosa* preparations, including extracts, fractions, isolated compounds, or traditional preparations. Comparator (C) included untreated groups, vehicle controls, placebo, standard treatments, or other relevant experimental comparators when applicable. Outcomes (O) included phytochemical composition, ethnomedicinal uses, and preclinical pharmacological activities.

After removing duplicates, 175 records remained and were screened by title and abstract. During this stage, 106 records were excluded, including 13 review articles and 93 records deemed irrelevant based on their titles or abstracts. The remaining 69 studies were assessed for eligibility. Subsequently, 19 reports were excluded: 10 because they did not meet the eligibility criteria and nine because they were outside the scope of the review. Ultimately, 50 studies met all inclusion criteria and constituted the constituted the final corpus included in the systematic review (Supplementary Table S2). The complete study selection process is summarized in the corresponding PRISMA 2020 flow diagram (Page et al., 2021) (Figure 1).

**FIGURE 1:**
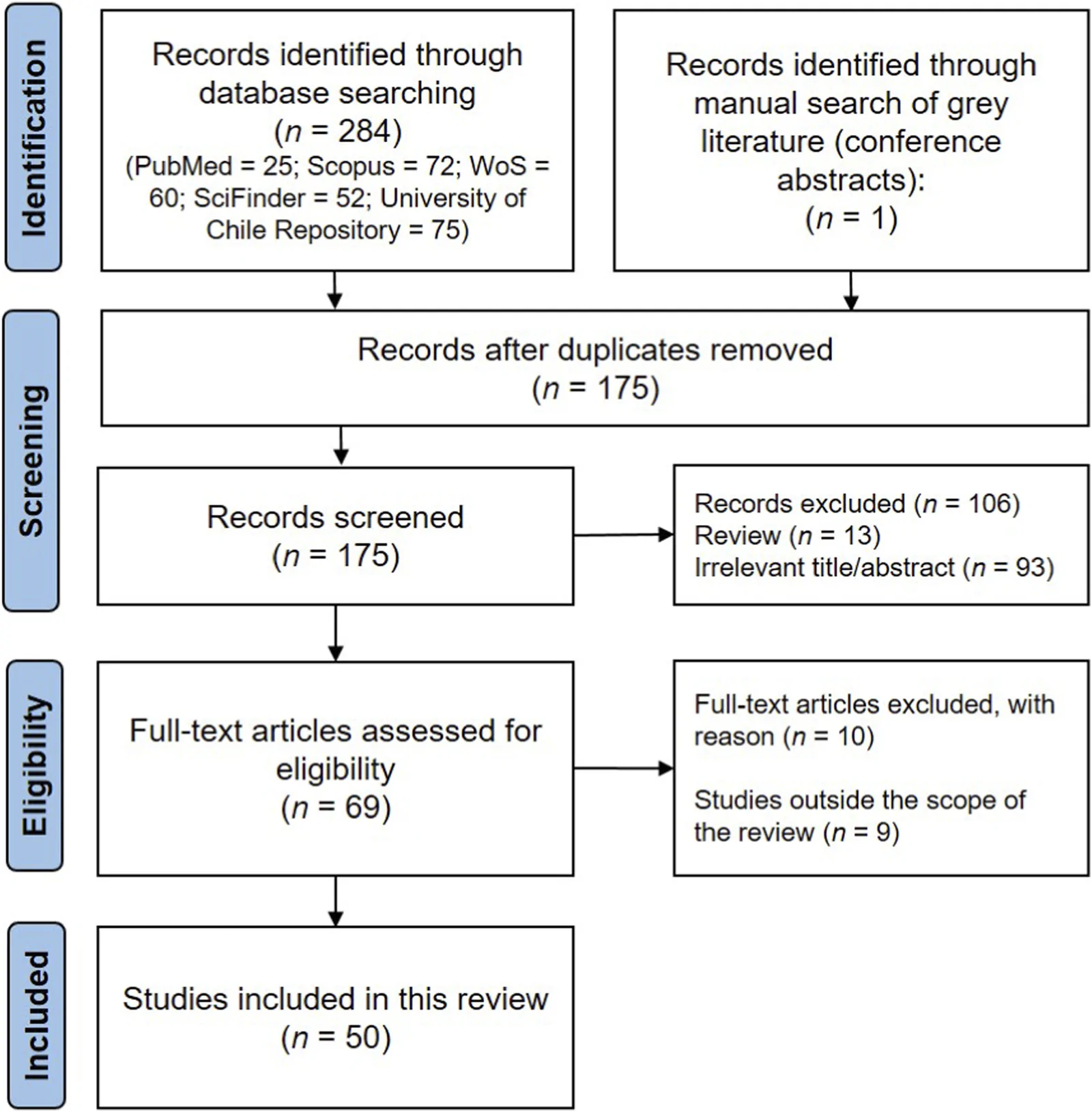
PRISMA 2020 flow diagram illustrating the identification, screening, eligibility, and inclusion of studies.

### 2.5 Data extraction

Data extraction was performed independently by two reviewers using a standardized data extraction form developed for this review. The following information was collected from each included study, when applicable: bibliographic information (authors and year of publication), study design, country of origin, study objectives, plant material used, extraction method and solvent, phytochemical composition, identified bioactive compounds, experimental models (*in vitro*, *in vivo*, clinical, or ethnobotanical), biological and pharmacological activities evaluated, main outcomes, and principal conclusions. For studies addressing cultivation, propagation, regulatory aspects, or industrial applications, the corresponding technical information was also extracted. Reviewers resolved discrepancies through discussion and, when necessary, by consulting a third reviewer.

### 2.6 Quality assessment

The methodological quality of the included studies was critically appraised by two reviewers independently. This assessment considered the clarity of the experimental design, the adequacy of controls, the description of procedures, reproducibility, sample size and sample type when applicable, the appropriateness of statistical analyses, and the completeness of reporting of results. Discrepancies were resolved by discussion and, when necessary, by consultation with a third reviewer. The study identified from a conference abstract was evaluated using the same quality criteria, based on the methodological information available in the abstract.

### 2.7 Risk of bias assessment

The risk of bias in the included animal studies was assessed using the SYRCLE risk-of-bias tool, which covers ten domains of internal validity. Each domain was classified as having a “Low,” “High,” or “Unclear” risk of bias. Two reviewers assessed the risk of bias independently and resolved discrepancies through discussion or consultation with a third reviewer (Hooijmans et al., 2014).

### 2.8 Structural classification

The chemical compounds identified in the biomass were classified by pathway and superclass using the NPClassifier tool (Kim et al., 2021). Structural similarity and compound property analyses were performed based on the literature.

### 2.9 ADME properties

The Absorption, Distribution, Metabolism, and Excretion (ADME) properties of the secondary metabolites were calculated using the SwissADME platform (Daina et al., 2017). The parameters analyzed included the number of atoms with hydrogen-bond acceptor capacity (H-bond acceptors), molar refractivity (MR), octanol/water partition coefficient estimated from solvation free energy in implicit solvents (iLogP), and aqueous solubility predicted directly from molecular structure (ESOL log S), along with the corresponding solubility classification (ESOL class). Additional descriptors comprised predicted gastrointestinal absorption (GI absorption), blood–brain barrier permeability (BBB permeant), and the probability of acting as a P-glycoprotein substrate (P-gp substrate). Furthermore, cytochrome P450 isoenzyme inhibition profiles (CYP2C19, CYP2C9, CYP2D6, and CYP3A4), the number of Lipinski’s rule-of-five violations, and the predicted oral bioavailability according to the Abbott score were also considered.

### 2.10 PAINS alert assessment

Structural alerts associated with Pan-assay interference compounds (PAINS) were evaluated individually for each compound using SwissADME (accessed August 2026).

## 3 Results

### 3.1 Phytochemistry of *Buddleja globosa*

#### 3.1.1 Polyphenolic compounds: Flavonoids and phenolic acids

The presence of flavonoids, such as luteolin and 6-hydroxyluteolin, was initially detected in the leaves of *B. globosa* (Harborne and Williams, 1971). Subsequently, linarin (acacetin-7-*O*-rutinoside) was isolated from leaves (Houghton and Hikino, 1989), and luteolin-7-*O*-glucoside and 6-hydroxyluteolin-7-*O*-glucoside were detected (Marin et al., 1979). Later, the presence of linarin, along with luteolin and 6-hydroxyluteolin, was confirmed again (Mensah et al., 2001). These flavonoids are widely distributed in the genus *Buddleja* and have been reported to exhibit multiple biological activities (Houghton, 1984; Mottaghipisheh et al., 2021).

Later, luteolin-7-*O*-glucoside (220 mg) and apigenin-7-*O*-glucoside (5 mg) were isolated from leaf extracts, compounds that have been further characterized in subsequent studies on *B. globosa* extracts (Backhouse et al., 2008a; 2008b; Parada et al., 2014). Anthocyanins and phenolic acids were also quantified in leaves using HPLC-DAD, including cyanidin (0.7 mg/mL), malvidin (37.7 mg/mL), benzoic acid (3.1 mg/mL), salicylic acid (4.6 mg/mL), caffeic acid (2.4 mg/mL), and vanillin (0.5 mg/mL) (Vera et al., 2023).

More recent studies using UHPLC-MS/MS have identified syringic acid, ferulic acid, chlorogenic acid, caffeic acid, pinocembrin, rutin, quercetin, luteolin, apigenin, and quercitrin in the leaves (Hernández et al., 2025). Caffeic acid, syringic acid, chlorogenic acid, cryptochlorogenic acid, luteolin, and chrysin were quantified in the leaves using UHPLC-MS/MS, with quercetin and rutin standing out as the major compounds (Quintero-Pertuz et al., 2025). It has also been characterized by its rutin content (867.1 ± 480 μg/g dry matter) and, in some studies, by the absence of detectable catechins (Castro-Saavedra et al., 2016). Other quantified polyphenols include luteolin (480.8 ± 40 μg/g), apigenin (319.6 ± 308 μg/g), coumaric acid (100.6 ± 45 μg/g), and caffeic acid (74.3 ± 30 μg/g) (Castro-Saavedra et al., 2016).

Various analytical technologies have been used to characterize *B. globosa* extracts. The commercial extract BG-126 was characterized by HPLC-DAD, identifying catechin, verbascoside, and quercetin (Araya et al., 2022; Ceriani et al., 2025). In lyophilized aqueous leaf extracts, quercetin, gallic acid, ferulic acid, and luteolin were identified by HPLC (Suwalsky et al., 2017).

LC-DAD-MS analyses identified numerous flavonoids and derivatives were identified in aqueous extracts of aerial parts, including caffeic acid, rutin, quercetin, quercetin 3-*O*-glucoside, luteolin 4′-*O*-rutinoside, robinin, pentoside of 3′ or 4′-*O*-quercetin, teucrol succinoyl glucoside, quercitrin, 4′-*O*-rutinoside of 7-*O*-methylated apigenin, 6-methoxy apigenin, 7-*O*-methylated apigenin, hexahydroxy flavone tetra-*O*-methylated, luteolin 6,8-di-C-glucoside succinate, and 3′-*O*-rutinoside of 7-methylated flavone isorhamnetin glucosyl-rhamnoside (Gastaldi et al., 2018).

Also, using LC-IT-MS/MS and LC-ESI-QTOF/MS-MS, a relevant fraction was identified in the leaves, composed mainly of 6-hydroxyluteolin-7-*O*-glucoside, quercetin-3-*O*-glucoside, luteolin-7-*O*-glucoside, and apigenin-7-*O*-glucoside (Torres-Vega et al., 2021).

Finally, in aqueous extracts of flowers, catechin (682.4 mg/100 g DW) and epicatechin (304.6 mg/100 g DW) were detected (Zamorano-Aguilar et al., 2020), and the compounds acacetin-7-*O*-rutinoside, apigenin-7-*O*-glucoside, quercetin-3-*O*-rutinoside, and scutellarein-7-*O*-glucoside have been isolated (Marin et al., 1979).

The phenolic compounds found in *B. globosa* are listed in Table 1, and structures are shown in Figure 2.

**TABLE 1:** Phenolic compounds identified in *Buddleja globosa*.

n°	Compound	Pathway	Superclass	Class	Identification	References
1	Luteolin	Shikimates and Phenylpropanoids	Flavonoids	Flavones	HPLC-UV + IS; HPLC-MS/MS + IS; UHPLC-MS/MS; HPLC-DAD + IS; HPLC + LC-DAD-MS	(Harborne and Williams, 1971; Mensah et al., 2001; Castro-Saavedra et al., 2016; Suwalsky et al., 2017; Hernández et al., 2025; Quintero-Pertuz et al., 2025)
2	6-hydroxyluteolin	Shikimates and Phenylpropanoids	Flavonoids	Flavones	HPLC-UV + IS	(Harborne and Williams, 1971; Mensah et al., 2001)
3	Luteolin 7-*O*-glucoside	Shikimates and Phenylpropanoids	Flavonoids	Flavones	^1^H and ^13^C NMR; HPLC-DAD + ^1^H and ^13^C NMR	(Marin et al., 1979; Backhouse et al., 2008a ; 2008b; Torres-Vega et al., 2021)
4	6-Hydroxyluteolin-7-*O*-glucoside	Shikimates and Phenylpropanoids	Flavonoids	Flavones	TLC; LC-IT-MS/MS + LC-ESI-QTOF/MS-MS	(Marin et al., 1979; Torres-Vega et al., 2021)
5	Apigenin 7-*O*-glucoside	Shikimates and Phenylpropanoids	Flavonoids	Flavones	^1^H NMR; HPLC–DAD + IS; LC-IT-MS/MS + LC-ESI-QTOF/MS-MS	(Marin et al., 1979; Backhouse et al., 2008a ; 2008b; Torres-Vega et al., 2021)
6	Rutin	Shikimates and Phenylpropanoids	Flavonoids	Flavonols	UHPLC-MS/MS; LC-DAD-MS + IS	(Castro-Saavedra et al., 2016; Gastaldi et al., 2018; Hernández et al., 2025; Quintero-Pertuz et al., 2025)
7	Quercetin	Shikimates and Phenylpropanoids	Flavonoids	Flavonols	UHPLC-MS/MS; HPLC-DAD; Raman spectra; HPLC-DAD + IS; LC-DAD-MS + IS	(Castro-Saavedra et al., 2016; Suwalsky et al., 2017; Gastaldi et al., 2018; Ceriani et al., 2025; Hernández et al., 2025; Quintero-Pertuz et al., 2025)
8	Apigenin	Shikimates and Phenylpropanoids	Flavonoids	Flavones	UHPLC-MS/MS	(Castro-Saavedra et al., 2016; Hernández et al., 2025)
9	Quercitrin	Shikimates and Phenylpropanoids	Flavonoids	Flavonols	UHPLC-MS/MS; LC-DAD-MS	(Gastaldi et al., 2018; Hernández et al., 2025)
10	Chrysin	Shikimates and Phenylpropanoids	Flavonoids	Flavones	UHPLC-MS/MS	(Quintero-Pertuz et al., 2025)
11	Quercetin 3-*O*-glucoside	Shikimates and Phenylpropanoids	Flavonoids	Flavonols	LC-DAD-MS + IS; LC-IT-MS/MS + LC-ESI-QTOF/MS-MS	(Gastaldi et al., 2018; Torres-Vega et al., 2021)
12	Luteolin 4′-*O*-rutinoside	Shikimates and Phenylpropanoids	Flavonoids	Flavones	LC-DAD-MS + IS	(Gastaldi et al., 2018)
13	Robinin	Shikimates and Phenylpropanoids	Flavonoids	Flavonols	LC-DAD-MS + IS	(Gastaldi et al., 2018)
14	4′-*O*-rutinoside of 7-*O*-methylated apigenin/Genkwanin 4′-*O*-rutinoside	Shikimates and Phenylpropanoids	Flavonoids	Flavones	LC-DAD-MS	(Gastaldi et al., 2018)
15	6-methoxy-apigenin/Hispidulin	Shikimates and Phenylpropanoids	Flavonoids	Flavones	LC-DAD-MS	(Gastaldi et al., 2018)
16	7-*O*-methylated apigenin/Genkwanin	Shikimates and Phenylpropanoids	Flavonoids	Flavones	LC-DAD-MS	(Gastaldi et al., 2018)
17	Isorhamnetin glucosyl-rhamnoside	Shikimates and Phenylpropanoids	Flavonoids	Flavonols	LC-DAD-MS	(Gastaldi et al., 2018)
18	Acacetin-7-*O*-rutinoside/Linarin	Shikimates and Phenylpropanoids	Flavonoids	Flavones	^1^H NMR; IS; HPLC-UV + IS	(Marin et al., 1979; Houghton and Hikino, 1989; Mensah et al., 2001)
19	Quercetin-3-*O*-rutinoside	Shikimates and Phenylpropanoids	Flavonoids	Flavonols	^1^H NMR	(Marin et al., 1979)
20	Scutellarein-7-*O*-glucoside	Shikimates and Phenylpropanoids	Flavonoids	Flavones	^1^H NMR	(Marin et al., 1979)
21	3′-*O*-rutinoside of 7-methylated flavone	Shikimates and Phenylpropanoids	Flavonoids	Flavones	LC-DAD-MS	(Gastaldi et al., 2018)
22	Luteolin 6,8-di-C-glucoside succinate	Shikimates and Phenylpropanoids	Flavonoids	Flavones	LC-DAD-MS	(Gastaldi et al., 2018)
23	Pinocembrin	Shikimates and Phenylpropanoids	Flavonoids	Flavanones	UHPLC-MS/MS	(Hernández et al., 2025)
24	Catechin	Shikimates and Phenylpropanoids	Flavonoids	Flavan-3-ols	HPLC-DAD + IS; Raman spectra	(Zamorano-Aguilar et al., 2020; Araya et al., 2022; Ceriani et al., 2025)
25	Epicatechin	Shikimates and Phenylpropanoids	Flavonoids	Flavan-3-ols	HPLC-DAD + IS	(Zamorano-Aguilar et al., 2020)
26	Cyanidin	Shikimates and Phenylpropanoids	Flavonoids	Anthocyanidins	HPLC-DAD + IS	(Vera et al., 2023)
27	Malvidin	Shikimates and Phenylpropanoids	Flavonoids	Anthocyanidins	HPLC-DAD + IS	(Vera et al., 2023)
28	Benzoic acid	Shikimates and Phenylpropanoids	Phenolic acids (C6-C1)	Simple phenolic acids	HPLC-DAD + IS	(Vera et al., 2023)
29	Salicylic acid	Shikimates and Phenylpropanoids	Phenolic acids (C6-C1)	Simple phenolic acids	HPLC-DAD + IS	(Vera et al., 2023)
30	Syringic acid	Shikimates and Phenylpropanoids	Phenolic acids (C6-C1)	Simple phenolic acids	UHPLC-MS/MS	(Hernández et al., 2025; Quintero-Pertuz et al., 2025)
31	Gallic acid	Shikimates and Phenylpropanoids	Phenolic acids (C6-C1)	Simple phenolic acids	HPLC-DAD + IS	(Suwalsky et al., 2017)

**FIGURE 2:**
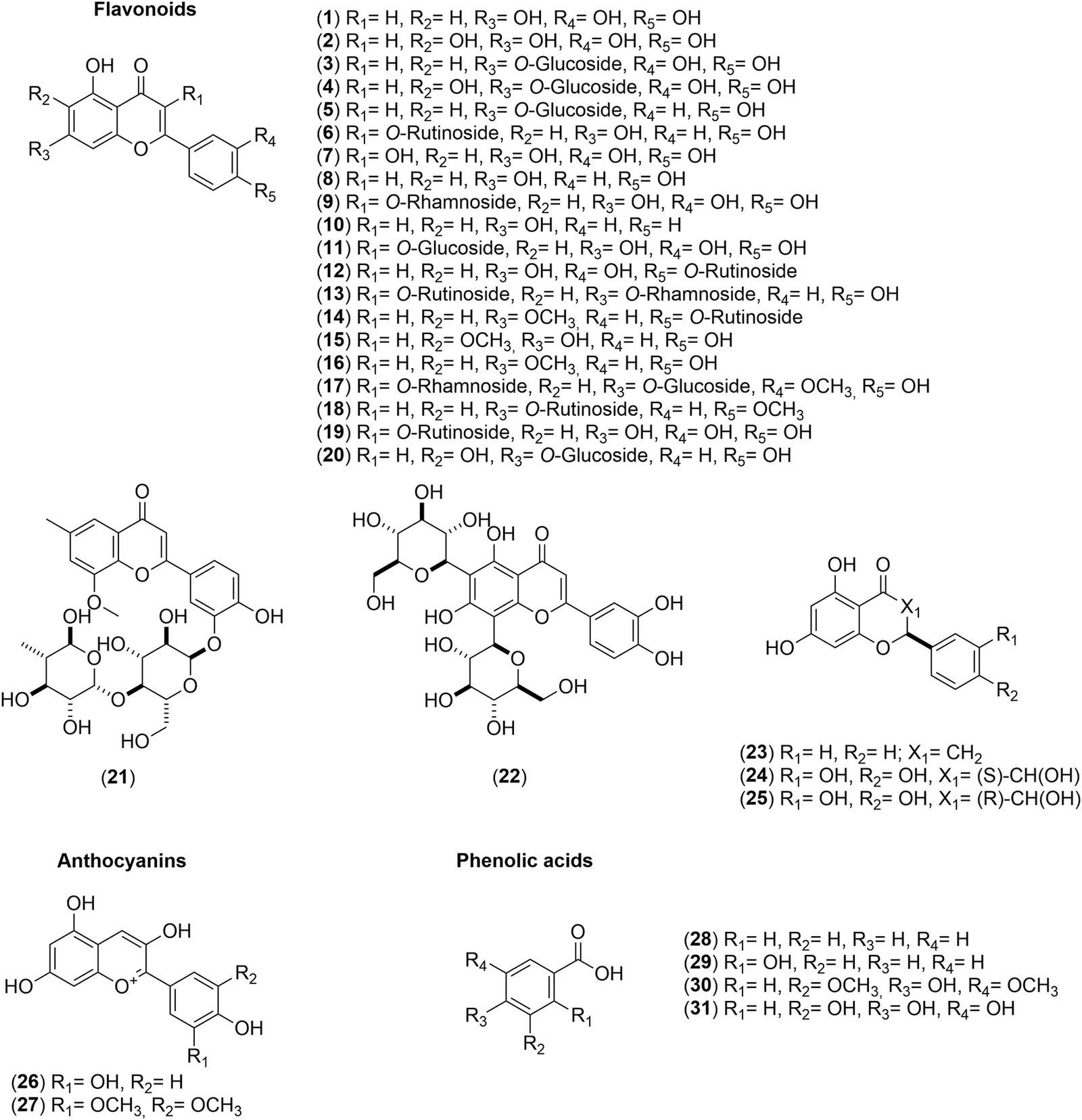
Chemical structures of flavonoids, anthocyanins, and phenolic acids identified from *Buddleja globosa.*

#### 3.1.2 Phenylpropanoids and phenylethanoids

Echinacoside and verbascoside have been isolated from the leaves of *B. globosa* on at least two occasions (Houghton, 1989; Mensah et al., 2001); regarding verbascoside yield, it is known that, from 4.8 kg of leaves, 350 mg could be isolated (Backhouse et al., 2008a; 2008b) and, from 12.4 g of methanolic extract, 72.4 mg could be isolated (Parada et al., 2014). Angoroside A, a phenylethanoid glycoside, was isolated from the ethanolic leaf extract of *B. globosa*, yielding 25 mg from the acetone fraction (Pardo et al., 1997).

Phenylpropanoid glycosides were identified in the leaves of *B. globosa* by LC-IT-MS/MS and LC-ESI-QTOF/MS/MS, highlighting verbascoside and isoverbascoside, along with their β-hydroxylated derivatives, as well as forsythoside A and B, campneoside I, lipedoside A, eukovoside, and martynoside (Torres-Vega et al., 2021). Additionally, LC-IT-MS/MS and LC-ESI-QTOF/MS/MS identified Caffeoyl shikimic acid in the leaves (Torres-Vega et al., 2021).

The stem of *B. globosa* has historically been discarded, but recent studies highlight its potential, with polyphenol concentrations of 2749.78 mg GAE/100 g DW and flavonoid concentrations of 2096.53 mg EC/100 g DW (Zamorano-Aguilar et al., 2025). The flowers have also been analyzed, with 6336.7 ± 224.3 mg GAE/100 g DW and 3331 ± 36.5 mg EC/100 g DW (Zamorano-Aguilar et al., 2020).

The total polyphenol content (TPC) and flavonoid content (TFC) of *B. globosa* vary considerably depending on the organ analyzed. In the leaves, concentrations of 15.93 ± 1.37 mg GAE/g dry weight and 2.57 ± 0.02 mg QE/g dry weight have been reported, values significantly higher than those found in the roots (2.40 mg GAE/g and 0.35 mg QE/g) (Chicaiza Coba and Fernández Rivero, 2025). An infusion of the leaves (2 g in 150 mL of water at 95 °C–100 °C for 5 min) yielded 165 ± 5 mg GAE/L of total phenols (Alarcón et al., 2008). Another study with a larger sample size reported TPC of 80.5 ± 58.7 mg GAE/g and TFC of 63.3 ± 5.2 mg QE/g (Castro-Saavedra et al., 2016).

The phenylpropanoids and phenylethanoids identified in *B. globosa* are summarized in Table 2, and their corresponding chemical structures are shown in Figure 3.

**TABLE 2:** Phenylpropanoids and phenylethanoids identified in *Buddleja globosa*.

n°	Compound	Pathway	Superclass	Class	Identification	References
32	Caffeic acid	Shikimates and Phenylpropanoids	Phenylpropanoids (C6-C3)	Cinnamic acids and derivatives	HPLC-DAD + IS; LC-DAD-MS + IS; UHPLC-MS/MS	(Gastaldi et al., 2018; Vera et al., 2023; Hernández et al., 2025; Quintero-Pertuz et al., 2025)
33	Ferulic acid	Shikimates and Phenylpropanoids	Phenylpropanoids (C6-C3)	Cinnamic acids and derivatives	HPLC-DAD + IS; UHPLC-MS/MS	(Suwalsky et al., 2017; Hernández et al., 2025)
34	*p*-coumaric acid	Shikimates and Phenylpropanoids	Phenylpropanoids (C6-C3)	Cinnamic acids and derivatives	UHPLC-MS/MS	(Castro-Saavedra et al., 2016)
35	Vanillin	Shikimates and Phenylpropanoids	Phenylpropanoids (C6-C3)	Cinnamic acids and derivatives	HPLC-DAD + IS	(Vera et al., 2023)
36	Caffeoyl shikimic acid	Shikimates and Phenylpropanoids	Phenylpropanoids (C6-C3)	Cinnamic acids and derivatives	LC-IT-MS-MS + LC-ESI-QTOF/MS-MS	(Torres-Vega et al., 2021)
37	Chlorogenic acid	Shikimates and Phenylpropanoids	Phenylpropanoids (C6-C3)	Cinnamic acids and derivatives	UHPLC-MS/MS	(Hernández et al., 2025; Quintero-Pertuz et al., 2025)
38	Cryptochlorogenic acid	Shikimates and Phenylpropanoids	Phenylpropanoids (C6-C3)	Cinnamic acids and derivatives	UHPLC-MS/MS	(Quintero-Pertuz et al., 2025)
39	Acteoside/verbascoside	Shikimates and Phenylpropanoids	Phenylethanoids (C6-C2)	Cinnamic acids and derivatives	MS + ^1^H NMR; HPLC-UV + IS; ^1^H and ^13^C NMR; HPLC -DAD + ^1^H and ^13^C NMR; LC-IT-MS-MS + LC-ESI-QTOF/MS-MS; HPLC-DAD + IS	(Houghton and Hikino, 1989; Mensah et al., 2001; Backhouse et al., 2008a ; 2008b; Parada et al., 2014; Torres-Vega et al., 2021; Araya et al., 2022)
40	Echinacoside	Shikimates and Phenylpropanoids	Phenylethanoids (C6-C2)	Cinnamic acids and derivatives	MS and ^1^H NMR; HPLC-UV + IS	(Houghton and Hikino, 1989; Mensah et al., 2001)
41	Isoverbascoside	Shikimates and Phenylpropanoids	Phenylethanoids (C6-C2)	Cinnamic acids and derivatives	LC-IT-MS-MS + LC-ESI-QTOF/MS-MS	(Torres-Vega et al., 2021)
42	Forsythoside A	Shikimates and Phenylpropanoids	Phenylethanoids (C6-C2)	Cinnamic acids and derivatives	LC-IT-MS-MS + LC-ESI-QTOF/MS-MS	(Torres-Vega et al., 2021)
43	Campneoside I	Shikimates and Phenylpropanoids	Phenylethanoids (C6-C2)	Cinnamic acids and derivatives	LC-IT-MS-MS + LC-ESI-QTOF/MS-MS	(Torres-Vega et al., 2021)
44	Martynoside	Shikimates and Phenylpropanoids	Phenylethanoids (C6-C2)	Cinnamic acids and derivatives	LC-IT-MS-MS + LC-ESI-QTOF/MS-MS	(Torres-Vega et al., 2021)
45	Lipedoside A	Shikimates and Phenylpropanoids	Phenylethanoids (C6-C2)	Cinnamic acids and derivatives	LC-IT-MS-MS + LC-ESI-QTOF/MS-MS	(Torres-Vega et al., 2021)
46	Eukovoside	Shikimates and Phenylpropanoids	Phenylpropanoids (C6-C3)	Cinnamic acids and derivatives	LC-IT-MS-MS + LC-ESI-QTOF/MS-MS	(Torres-Vega et al., 2021)
47	Forsythoside B	Shikimates and Phenylpropanoids	Phenylethanoids (C6-C2)	Cinnamic acids and derivatives	LC-IT-MS-MS + LC-ESI-QTOF/MS-MS	(Torres-Vega et al., 2021)
48	Angoroside A	Shikimates and Phenylpropanoids	Phenylpropanoids (C6-C3)	Cinnamic acids and derivatives	^1^H and ^13^C NMR	(Pardo et al., 1997)

**FIGURE 3:**
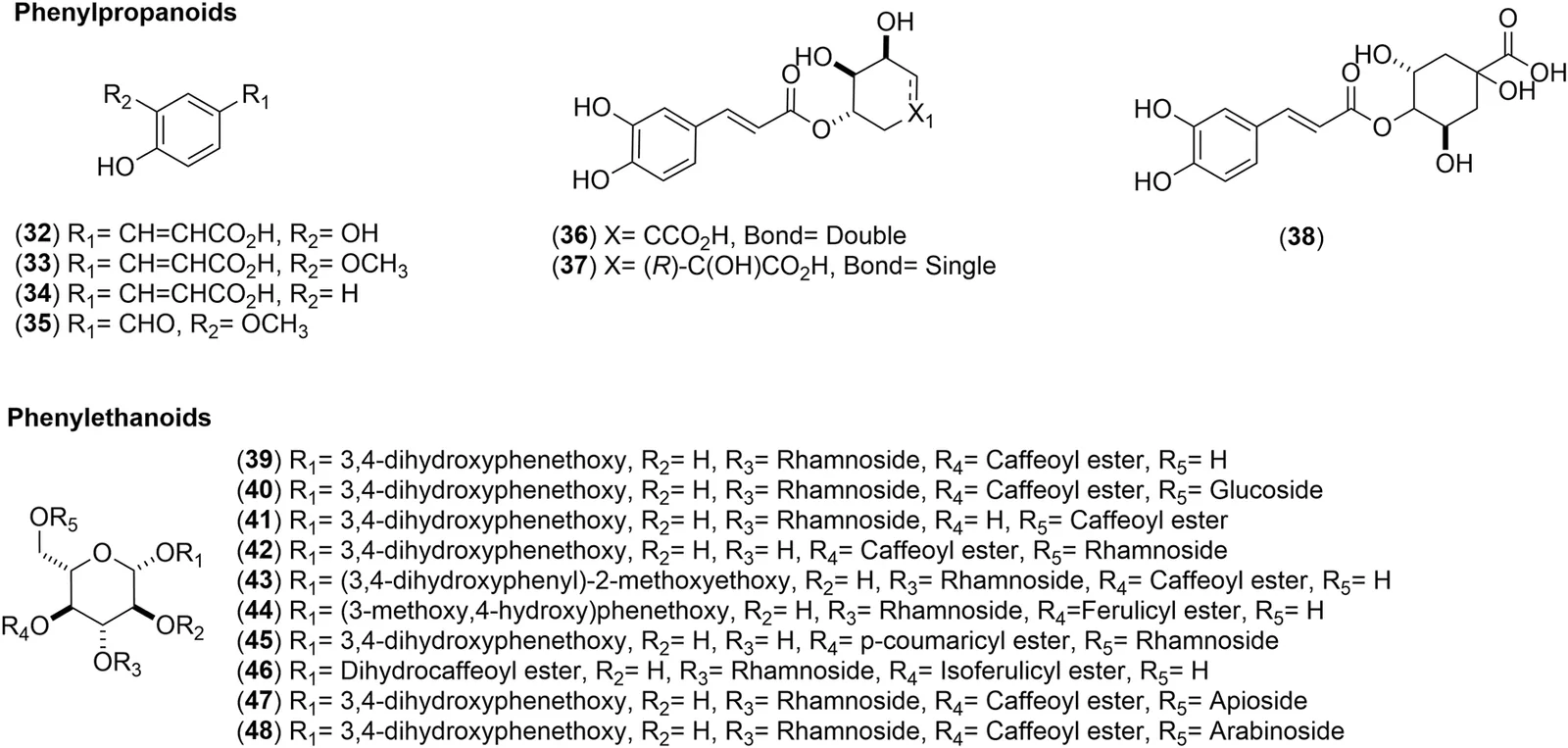
Chemical structures of phenylpropanoids and phenylethanoids identified from *Buddleja globosa.*

### 3.2 Terpenoids

#### 3.2.1 Iridoids

The first compound isolated from the leaves of *B. globosa* was aucubin (Trim and Hill, 1952). Years later, other compounds besides aucubin and catalpol were also isolated from the leaves (Duff et al., 1965). Subsequently, the isolation of *p*-methoxycinnamoyl-aucubin, *p*-methoxycinnamoyl-catalpol, catalposide, catalpol, and again aucubin was reported (Houghton and Hikino, 1989). More recently, 6-*O*-methylcatalpol was isolated from the leaves (Quintero-Pertuz et al., 2025).

The iridoids identified in *B. globosa* are summarized in Table 3, and their corresponding chemical structures are shown in Figure 4.

**TABLE 3:** Iridoids identified in *Buddleja globosa*.

n°	Compound	Pathway	Superclass	Class	Identification	References
49	Aucubin	Terpenoids	Monoterpenoids	Iridoid monoterpenoids	Isolation; MS and ^1^H -NMR	(Trim and Hill, 1952; Duff et al., 1965; Houghton and Hikino, 1989)
50	*p*-methoxycinnamoyl-aucubin	Terpenoids	Monoterpenoids	Iridoid monoterpenoids	MS and ^1^H-NMR	(Houghton and Hikino, 1989)
51	Catalpol	Terpenoids	Monoterpenoids	Iridoid monoterpenoids	Isolation	(Duff et al., 1965; Houghton and Hikino, 1989)
52	*p*-methoxycinnamoyl-catalpol	Terpenoids	Monoterpenoids	Iridoid monoterpenoids	MS and ^1^H-NMR	(Houghton and Hikino, 1989)
53	Catalposide	Terpenoids	Monoterpenoids	Iridoid monoterpenoids	MS and ^1^H-NMR	(Houghton and Hikino, 1989)
54	6-*O*-methylcatalpol	Terpenoids	Monoterpenoids	Iridoid monoterpenoids	^1^H and ^13^C NMR	(Quintero-Pertuz et al., 2025)

**FIGURE 4:**
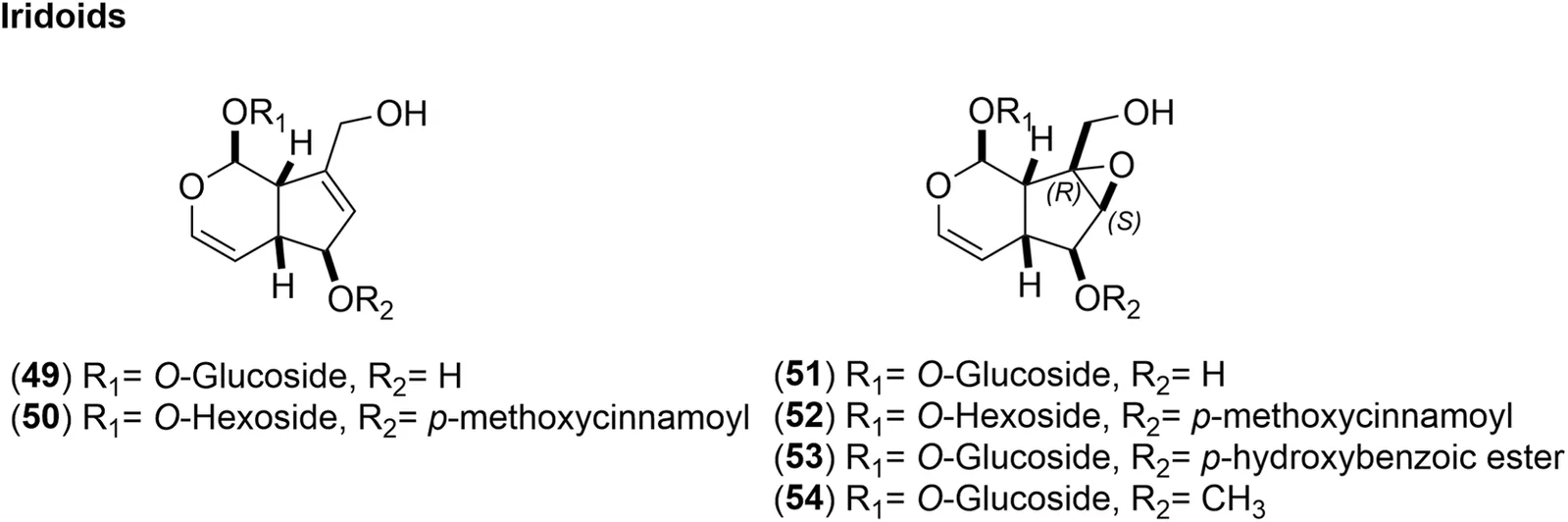
Chemical structures of iridoids identified from *Buddleja globosa.*

#### 3.2.2 Other terpenoids

The chemical characterization of the woody and subterranean organs of *B. globosa* has enabled the identification of various terpene compounds. From the stem bark, the diterpenes buddlejone and deoxybuddlejone (the latter reported as a new compound), the bisditerpene maytenone, and the sesquiterpenes buddledin A and B were isolated (Mensah et al., 2000). Buddlejone had previously been isolated from the root bark of *B. globosa* (Houghton et al., 1996).

In contrast, analysis of the chloroform extract of the root revealed a high content of terpenoids, with buddledin A as the major component, yielding 800 mg. In this organ, lower concentrations of dihydrobuddledin A (60 mg), buddledin C (25 mg), buddledin B (22 mg), zerumbone (17 mg), as well as buddledones A and B (6.4 mg and 7.3 mg, respectively) were also identified (Liao et al., 1999). From the fractionation of the hexane extract (HE) of the leaves, a mixture of α- and β-amyrins (2:3) was obtained, which was identified by GC-MS (Backhouse et al., 2008b). The triterpene lupeol was isolated from the leaves (Marin et al., 1979), and the commercial extract of *B. globosa* BG-126 has been reported to contain thymol and carvacrol, among other metabolites (Ceriani et al., 2025).

The other terpenoids identified in *B. globosa* are summarized in Table 4, and their corresponding chemical structures are shown in Figure 5.

**TABLE 4:** Other terpenoids identified in *Buddleja globosa*.

n°	Compound	Pathway	Superclass	Class	Identification	References
55	Buddlejone	Terpenoids	Diterpenoids	Abietane diterpenoids	^1^H and ^13^C NMR	(Houghton et al., 1996; Mensah et al., 2000)
56	Desoxybuddlejone	Terpenoids	Diterpenoids	Abietane diterpenoids	^1^H and ^13^C NMR + MS	(Mensah et al., 2000)
57	Maytenone	Terpenoids	Triterpenoids	Fernane and Arborinane triterpenoids	^1^H and ^13^C NMR	(Mensah et al., 2000)
58	Lupeol	Terpenoids	Triterpenoids	Lupane triterpenoids	Isolation + IS	(Marin et al., 1979)
59	α-amyrin	Terpenoids	Triterpenoids	Ursane and Taraxastane triterpenoids	GC-MS	(Backhouse et al., 2008b)
60	β-amyrin	Terpenoids	Triterpenoids	Oleanane triterpenoids	GC-MS	(Backhouse et al., 2008b)

**FIGURE 5:**
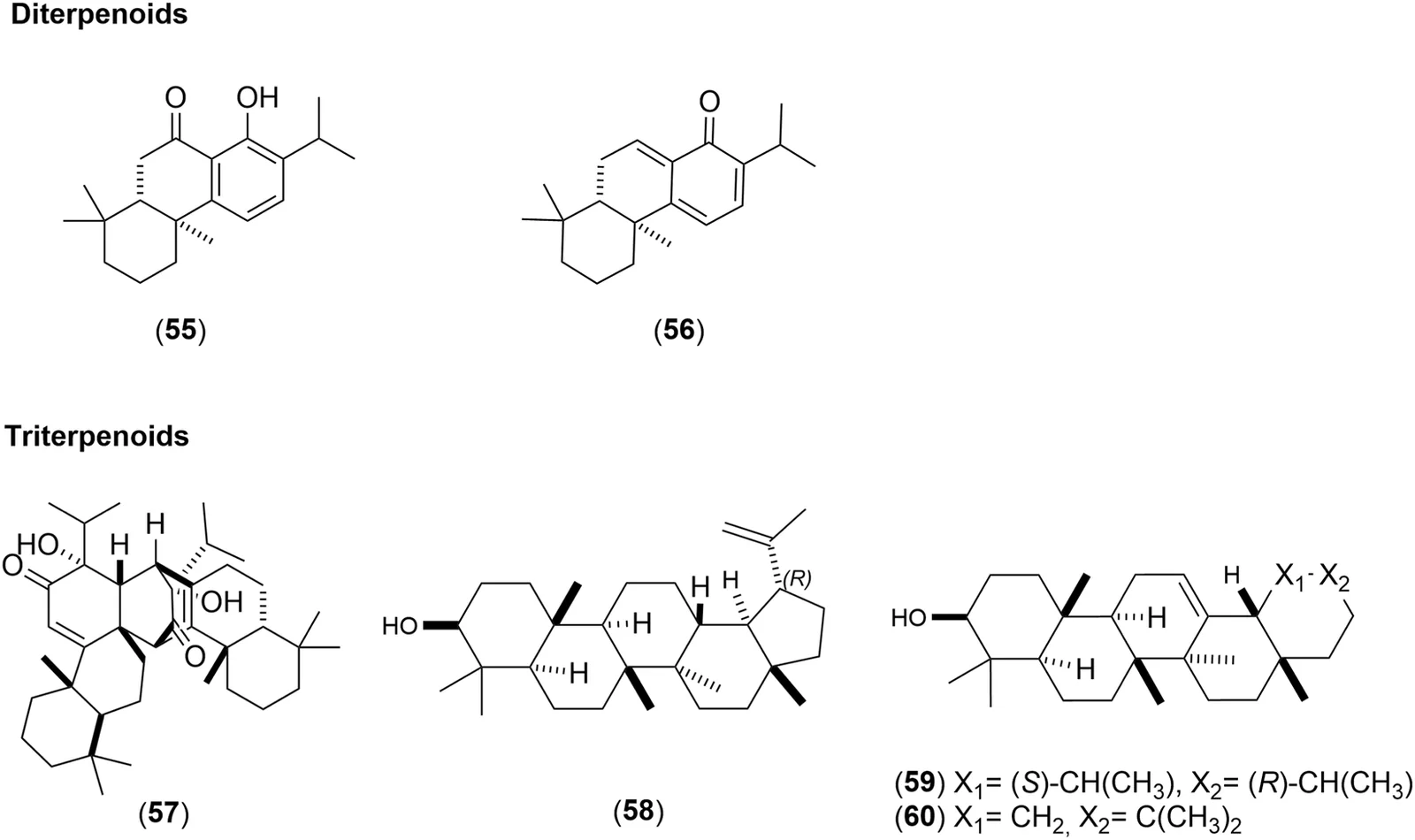
Chemical structures of diterpenoids and triterpenoids identified from *Buddleja globosa.*

#### 3.2.3 Steroids

From the serial dichloromethane (DCM) extract of *B. globosa* leaves, a steroidal mixture was obtained, whose constituents were identified by GC-MS as β-sitosterol and stigmasterol (major components), along with campesterol, stigmastanol, and ergosterol (Backhouse et al., 2008b). Additionally, β-sitosterol glucoside was isolated as a single compound and identified by spectral data and comparison with an authentic standard (Backhouse et al., 1997).

Table 5 summarizes the steroids identified in *B. globosa*, and Figure 6 shows their corresponding chemical structures.

**TABLE 5:** Steroids identified in *Buddleja globosa*.

n°	Compound	Pathway	Superclass	Class	Identification	References
61	β-sitosterol	Terpenoids	Steroids	Stigmastane steroids	GC-MS	(Backhouse et al., 2008b)
62	β-sitosterol glucoside	Terpenoids	Steroids	Stigmastane steroids	^1^H and ^13^C NMR + IS	(Backhouse et al., 2008b)
63	Stigmasterol	Terpenoids	Steroids	Cholestane steroids	GC-MS	(Backhouse et al., 2008b)
64	Campesterol	Terpenoids	Steroids	Cholestane steroids	GC-MS	(Backhouse et al., 2008b)
65	Stigmastanol	Terpenoids	Steroids	Cholestane steroids	GC-MS	(Backhouse et al., 2008b)
66	Ergosterol	Terpenoids	Steroids	Ergostane steroids	GC-MS	(Backhouse et al., 2008b)

**FIGURE 6:**
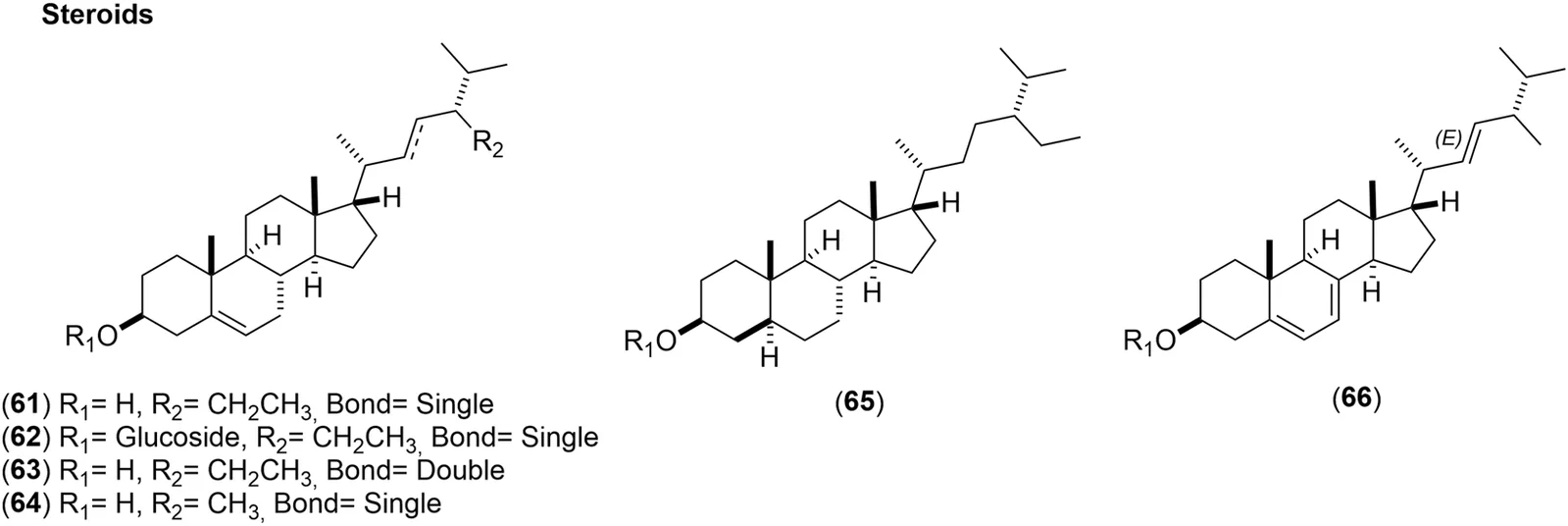
Chemical structures of steroids identified from *Buddleja globosa.*

### 3.3 Essential oils

The essential oil of *B. globosa* has only been analyzed on one occasion; its composition was determined by GC-FID and GC-MS, finding α-pinene, camphene, sabinene, β-pinene, myrcene, *p*-cymene, limonene, γ-terpinene, 1,8-cineole, *cis*-sabinene hydrate, linalool, *cis*-thujone, *trans*-thujone, *cis*-*p*-mentha-2,8-dien-1-ol, *trans*-sabinol, camphor, thujanol, citronellal, *cis*-chrysanthenol, borneol, terpinen-4-ol, thuj-3-en-10-al, α-terpineol, dihydrocarveol, *trans*-carveol, carvone, thymol, carvacrol, α-humulene, γ-gurjunene, γ-cadinene, δ-cadinene, (*E*)-caryophyllene, elemol, spathulenol, caryophyllene oxide, β-cedrene epoxide, caryophylla-4(12),8(13)-dien-5-ol, murrolol, bulnesol, sabina ketone, and α-curcumene (Pellegrini et al., 2017). In contrast, other species of the genus *Buddleja* have been the subject of numerous studies on their essential oils. For example, analyses of essential oils obtained from the aerial parts of *Buddleja perfoliata* Kunth (Juárez et al., 2016), *B. tucumanensis* (Lorenzo et al., 2006), *Buddleja davidii* Franch. and *Buddleja asiatica* Lour. (Joshi et al., 2012), as well as from the flowers of *Buddleja alternifolia* Maxim. (Yang et al., 2020) and from the leaves and flowers of *Buddleja polystachya* Fresen. have been reported (Ati and Fawzy, 2014).

The compounds of the essential oil of *B. globosa* are summarized in Table 6, and their corresponding chemical structures of monoterpenoids and sesquiterpenoids are shown in Figure 7; Figure 8, respectively.

**TABLE 6:** Compounds identified in the essential oil of *Buddleja globosa*.

n°	Compound	Pathway	Superclass	Class	Identification	References
67	Thymol	Terpenoids	Monoterpenoids	Menthane monoterpenoids	Raman spectra; GC-FID + GC-MS	(Pellegrini et al., 2017; Ceriani et al., 2025)
68	Carvacrol	Terpenoids	Monoterpenoids	Menthane monoterpenoids	Raman spectra; GC-FID + GC-MS	(Pellegrini et al., 2017; Ceriani et al., 2025)
69	*p*-cymene	Terpenoids	Monoterpenoids	Menthane monoterpenoids	GC-FID + GC-MS	(Pellegrini et al., 2017)
70	Myrcene	Terpenoids	Monoterpenoids	Acyclic monoterpenoids	GC-FID + GC-MS	(Pellegrini et al., 2017)
71	Linalool	Terpenoids	Monoterpenoids	Acyclic monoterpenoids	GC-FID + GC-MS	(Pellegrini et al., 2017)
72	Citronellal	Terpenoids	Monoterpenoids	Acyclic monoterpenoids	GC-FID + GC-MS	(Pellegrini et al., 2017)
73	*cis*-thujone	Terpenoids	Monoterpenoids	Thujane monoterpenoids	GC-FID + GC-MS	(Pellegrini et al., 2017)
74	*trans*-thujone	Terpenoids	Monoterpenoids	Thujane monoterpenoids	GC-FID + GC-MS	(Pellegrini et al., 2017)
75	Thujanol	Terpenoids	Monoterpenoids	Thujane monoterpenoids	GC-FID + GC-MS	(Pellegrini et al., 2017)
76	*trans*-sabinol	Terpenoids	Monoterpenoids	Menthane monoterpenoids	GC-FID + GC-MS	(Pellegrini et al., 2017)
77	sabina ketone	Terpenoids	Monoterpenoids	Thujane monoterpenoids	GC-FID + GC-MS	(Pellegrini et al., 2017)
78	*cis*-sabinene hydrate	Terpenoids	Monoterpenoids	Thujane monoterpenoids	GC-FID + GC-MS	(Pellegrini et al., 2017)
79	Sabinene	Terpenoids	Monoterpenoids	Thujane monoterpenoids	GC-FID + GC-MS	(Pellegrini et al., 2017)
80	Thuj-3-en-10-al	Terpenoids	Monoterpenoids	Thujane monoterpenoids	GC-FID + GC-MS	(Pellegrini et al., 2017)
81	Borneol	Terpenoids	Monoterpenoids	Camphane monoterpenoids	GC-FID + GC-MS	(Pellegrini et al., 2017)
82	Camphor	Terpenoids	Monoterpenoids	Camphane monoterpenoids	GC-FID + GC-MS	(Pellegrini et al., 2017)
83	Camphene	Terpenoids	Monoterpenoids	Camphane monoterpenoids	GC-FID + GC-MS	(Pellegrini et al., 2017)
84	α-pinene	Terpenoids	Monoterpenoids	Pinane monoterpenoids	GC-FID + GC-MS	(Pellegrini et al., 2017)
85	β-pinene	Terpenoids	Monoterpenoids	Pinane monoterpenoids	GC-FID + GC-MS	(Pellegrini et al., 2017)
86	1,8-cineole	Terpenoids	Monoterpenoids	Menthane monoterpenoids	GC-FID + GC-MS	(Pellegrini et al., 2017)
87	*cis*-chrysanthenol	Terpenoids	Monoterpenoids	Pinane monoterpenoids	GC-FID + GC-MS	(Pellegrini et al., 2017)
88	Limonene	Terpenoids	Monoterpenoids	Menthane monoterpenoids	GC-FID + GC-MS	(Pellegrini et al., 2017)
89	Terpinen-4-ol	Terpenoids	Monoterpenoids	Menthane monoterpenoids	GC-FID + GC-MS	(Pellegrini et al., 2017)
90	α-terpineol	Terpenoids	Monoterpenoids	Menthane monoterpenoids	GC-FID + GC-MS	(Pellegrini et al., 2017)
91	*trans*-carveol	Terpenoids	Monoterpenoids	Menthane monoterpenoids	GC-FID + GC-MS	(Pellegrini et al., 2017)
92	Carvone	Terpenoids	Monoterpenoids	Menthane monoterpenoids	GC-FID + GC-MS	(Pellegrini et al., 2017)
93	γ-terpinene	Terpenoids	Monoterpenoids	Monocyclic monoterpenoids	GC-FID + GC-MS	(Pellegrini et al., 2017)
94	*cis*-*p*-mentha-2,8-dien-1-ol	Terpenoids	Monoterpenoids	Menthane monoterpenoids	GC-FID + GC-MS	(Pellegrini et al., 2017)
95	Dihydrocarveol	Terpenoids	Monoterpenoids	Menthane monoterpenoids	GC-FID + GC-MS	(Pellegrini et al., 2017)
96	Buddledin B	Terpenoids	Sesquiterpenoids	Caryophyllane sesquiterpenoids	^1^H and ^13^C NMR	(Liao et al., 1999; Mensah et al., 2000)
97	Dihydrobuddledin A	Terpenoids	Sesquiterpenoids	Caryophyllane sesquiterpenoids	^1^H and ^13^C NMR + MS	(Liao et al., 1999)
98	Caryophylla-4(12),8(13)-dien-5-ol	Terpenoids	Sesquiterpenoids	Caryophyllane sesquiterpenoids	GC-FID + GC-MS	(Pellegrini et al., 2017)
99	(*E*)-caryophyllene	Terpenoids	Sesquiterpenoids	Caryophyllane sesquiterpenoids	GC-FID + GC-MS	(Pellegrini et al., 2017)
100	Buddledin A	Terpenoids	Sesquiterpenoids	Humulane sesquiterpenoids	^1^H and ^13^C NMR	(Liao et al., 1999; Mensah et al., 2000)
101	Buddledin C	Terpenoids	Sesquiterpenoids	Caryophyllane sesquiterpenoids	^1^H and ^13^C NMR	(Liao et al., 1999)
102	Zerumbone	Terpenoids	Sesquiterpenoids	Humulane sesquiterpenoids	^1^H and ^13^C NMR	(Liao et al., 1999)
103	Buddledone A	Terpenoids	Sesquiterpenoids	Humulane sesquiterpenoids	^1^H and ^13^C NMR + MS	(Liao et al., 1999)
104	Buddledone B	Terpenoids	Sesquiterpenoids	Eudesmane sesquiterpenoids	^1^H and ^13^C NMR + MS	(Liao et al., 1999)
105	γ-cadinene	Terpenoids	Sesquiterpenoids	Cadinane sesquiterpenoids	GC-FID + GC-MS	(Pellegrini et al., 2017)
106	δ-cadinene	Terpenoids	Sesquiterpenoids	Cadinane sesquiterpenoids	GC-FID + GC-MS	(Pellegrini et al., 2017)
107	Murrolol	Terpenoids	Sesquiterpenoids	Cadinane sesquiterpenoids	GC-FID + GC-MS	(Pellegrini et al., 2017)
108	γ-gurjunene	Terpenoids	Sesquiterpenoids	Aromadendrane sesquiterpenoids	GC-FID + GC-MS	(Pellegrini et al., 2017)
109	Bulnesol	Terpenoids	Sesquiterpenoids	Guaiane sesquiterpenoids	GC-FID + GC-MS	(Pellegrini et al., 2017)
110	Elemol	Terpenoids	Sesquiterpenoids	Elemane sesquiterpenoids	GC-FID + GC-MS	(Pellegrini et al., 2017)
111	Spathulenol	Terpenoids	Sesquiterpenoids	Aromadendrane sesquiterpenoids	GC-FID + GC-MS	(Pellegrini et al., 2017)
112	Caryophyllene oxide	Terpenoids	Sesquiterpenoids	Caryophyllane sesquiterpenoids	GC-FID + GC-MS	(Pellegrini et al., 2017)
113	β-cedrene epoxide	Terpenoids	Sesquiterpenoids	Cedrane and Isocedrane sesquiterpenoids	GC-FID + GC-MS	(Pellegrini et al., 2017)
114	α-curcumene	Terpenoids	Sesquiterpenoids	Bisabolane sesquiterpenoids	GC-FID + GC-MS	(Pellegrini et al., 2017)
115	α-humulene	Terpenoids	Sesquiterpenoids	Humulane sesquiterpenoids	GC-FID + GC-MS	(Pellegrini et al., 2017)

**FIGURE 7:**
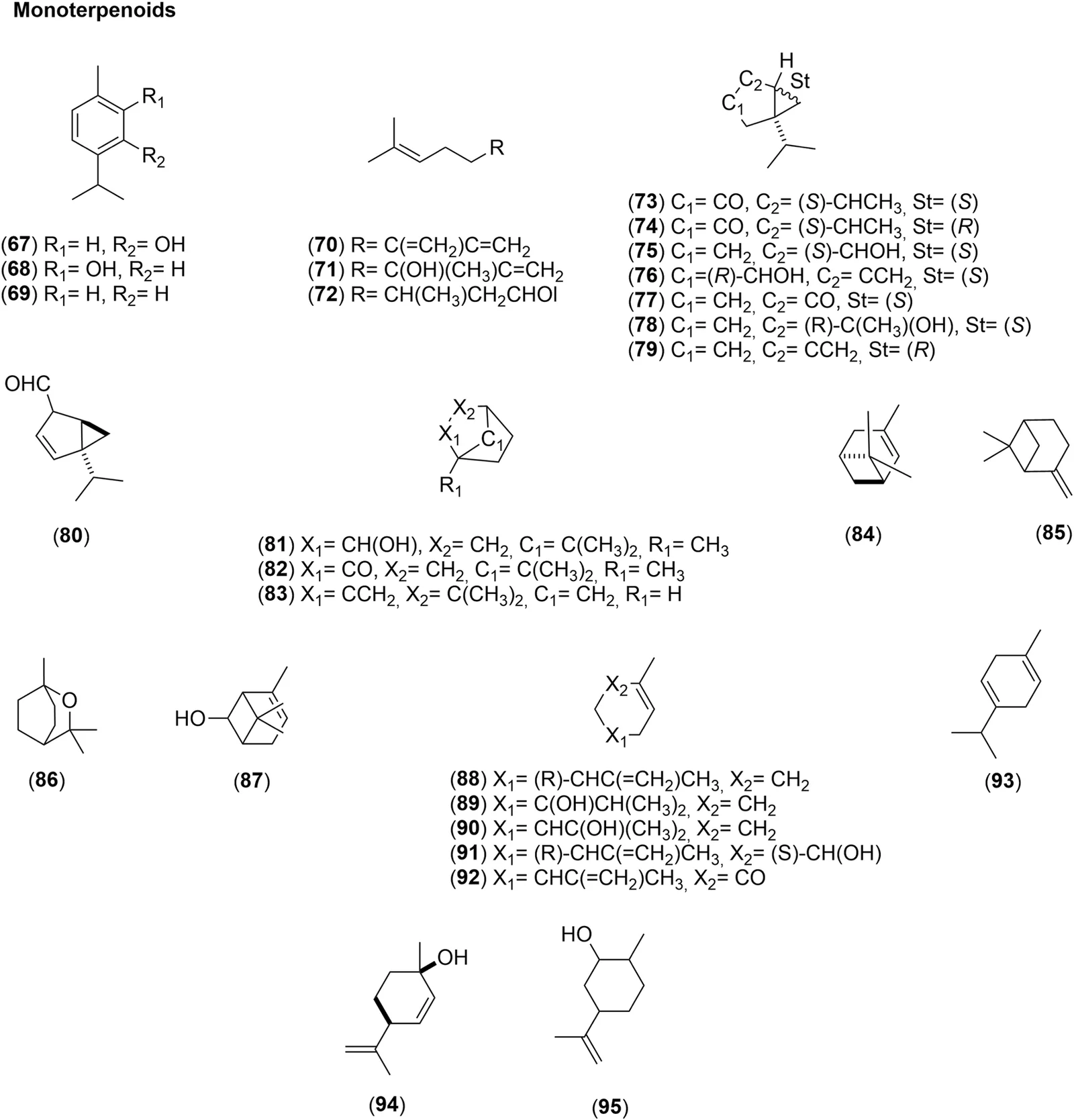
Chemical structures of monoterpenoids identified in the essential oil of *Buddleja globosa*.

**FIGURE 8:**
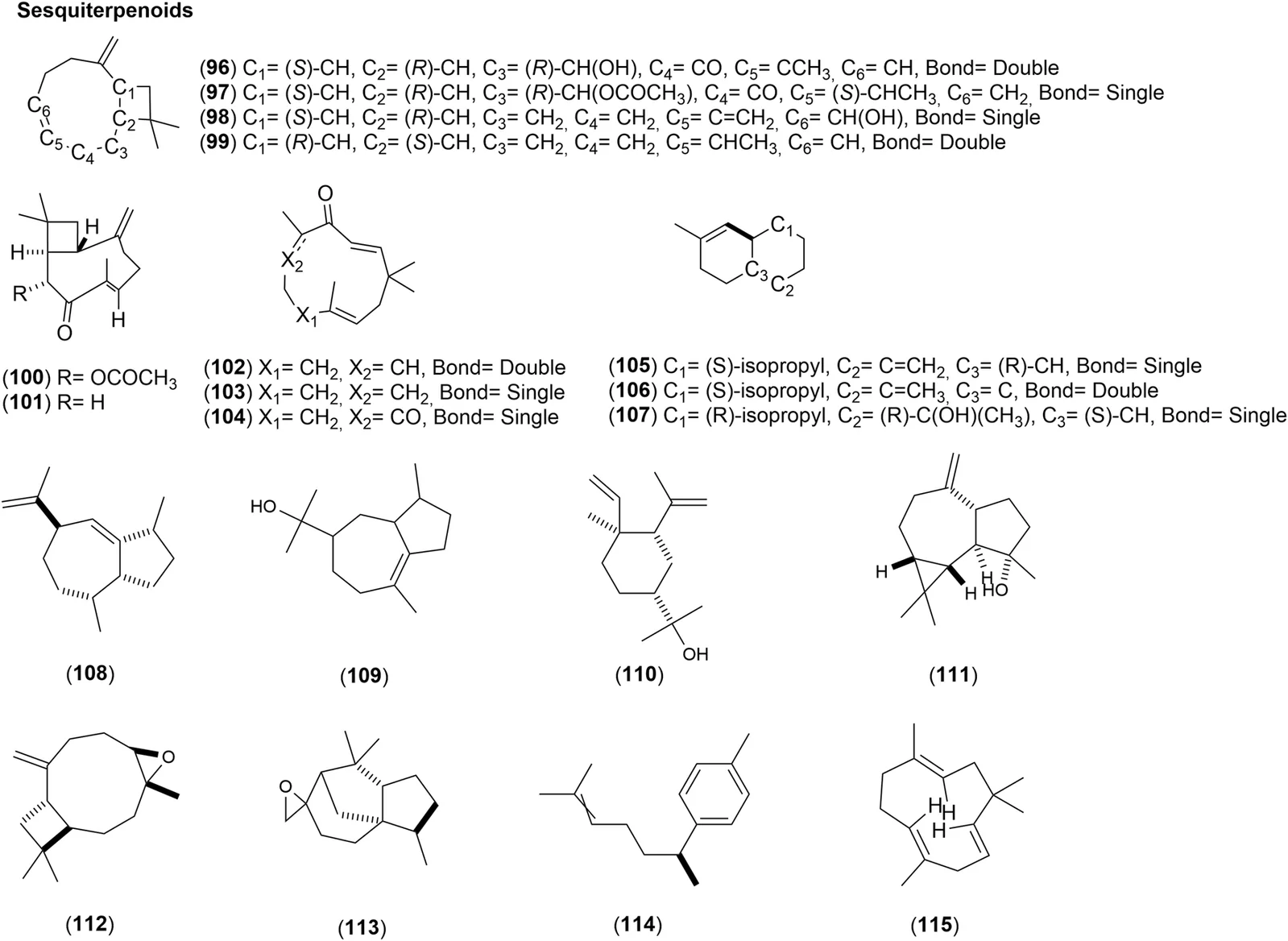
Chemical structures of sesquiterpenoids identified in the essential oil of *Buddleja globosa*.

### 3.4 Polyketides

Houghton (1989) reported the occurrence of polyketide-derived constituents in *B. globosa*, including 2-(4-hydroxyphenyl)ethyl esters of long-chain fatty acids such as lignoceric and docosanoic acids, as well as lignoceryl 4-hydroxycinnamyl alcohol (Houghton, 1989). The polyketides identified in *B. globosa* are summarized in Table 7, and their corresponding chemical structures are shown in Figure 9.

**TABLE 7:** Polyketides identified in *Buddleja globosa*.

n°	Compound	Pathway	Superclass	Class	Identification	References
116	2-(4-hydroxyphenyl)ethanol ester of lignoceric acid	Polyketides	Aromatic polyketides	Catechols with side chains	^1^H and ^13^C NMR + MS	(Houghton, 1989)
117	Ester of 2-(4-hydroxyphenyl) ethanol of docosanoic acid/2-(4-hydroxyphenyl)ethyl dodecanoate	Polyketides	Aromatic polyketides	Catechols with side chains	^1^H and ^13^C NMR + MS	(Houghton, 1989)
118	Ester of lignoceric acid and 4-hydroxycinnamyl alcohol/lignoceryl 4-hydroxycinnamyl alcohol	Polyketides	Aromatic polyketides	Catechols with side chains	^1^H and ^13^C NMR + MS	(Houghton, 1989)
119	Ester of 4-hydroxycinnamyl alcohol and docosanoic acid	Polyketides	Aromatic polyketides	Catechols with side chains	^1^H and ^13^C NMR + MS	(Houghton, 1989)

**FIGURE 9:**
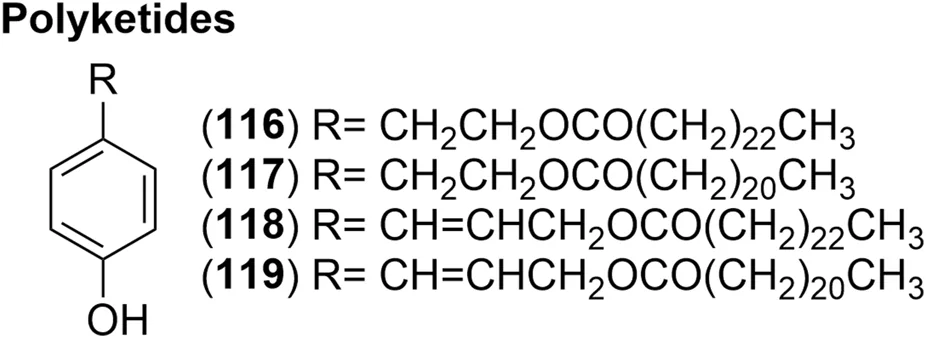
Chemical structures of polyketides identified from *Buddleja globosa.*

## 4 Traditional, cultural, and ethnopharmacological uses of *B. globosa*

The medicinal use of *B. globosa* has been based primarily on its leaves, which have been consumed and used therapeutically for over a century in Chilean tradition, where the species has become established as one of the most effective vulneraries for tissue repair (Mellado Gutiérrez, 1923). In the southern part of the country, it is common to use its leaves in decoction or infusion for washing wounds (Oyarzo, 2024). Historically, the dried leaves were ground into a fine powder and applied directly to skin lesions to accelerate healing (Mellado Gutiérrez, 1923; Muñoz et al., 1981). It shares skin-protective properties with other species of the genus *Buddleja* (Houghton, 1984; Hernández-Pasteur et al., 2019).

This therapeutic versatility is especially evident in treating external wounds, positioning the species as a key component of Chilean herbal medicine (Muñoz, 2001). Beyond its external regenerative capacity, the leaves of *B. globosa* are attributed with internal therapeutic properties. They are therefore recommended to treat simple gastric ulcers, chronic dysentery, and intestinal catarrh. Folk medicine also attributes protective properties to them for the liver, with the infusion administered to treat liver ailments and abscesses, as well as a soothing effect in cases of hemoptysis and purulent processes (Mellado Gutiérrez, 1923; Muñoz et al., 1981).

The use of *B. globosa* is an essential part of Chile’s biocultural heritage, highlighting the importance of Indigenous peoples' ancestral knowledge, particularly that of the Mapuche (Rodríguez-Díaz, 2025). Along with bailahuen (*Haplopappus bailahuen* J.Rémy) [Asteraceae] (Mitsi and Echeverría, 2024) and boldo (*Peumus boldus* Molina) [Monimiaceae] (Cassels et al., 2019), it stands out as one of the few native species with a widespread presence in both urban domestic consumption and the development of higher-value-added products (Fuentes-Barros et al., 2026).

In the global context of integrating diverse medical systems, phytotherapy is consolidating its position as a fundamental pillar, supported by a growing body of scientific evidence from preclinical and, increasingly, clinical studies. Under Chile’s current intercultural approach to health, it is urgent to integrate scientific rigor with traditional medicine (Fuentes-Barros et al., 2025a). This synergy is key to addressing national regulatory challenges and ensuring the rational, safe, and sustainable use of phytotherapeutic resources. In line with the World Health Organization’s (WHO) objectives on the preventive role and access to herbal medicine, these species are fundamental to popular consumption and traditional Chilean medicine, especially for treating dermatological and gastric conditions. Furthermore, the consumption of *B. globosa* remains prevalent among rural and indigenous communities in central-north Chile (Hernández et al., 2025), with traditional cultural and therapeutic significance for the Diaguita people (Cortés, 2025).

*Buddleja globosa* use also occurs among older adults attending Primary Healthcare (PHC) centers in southwestern Santiago, Chile’s capital (Bridi et al., 2023). Its use is also common in rural areas of the Metropolitan Region (Barraza et al., 2014), in the Ñuble Region (Burgos and Morales, 2010), and in Chol Chol, a town in the province of Cautín (Torri, 2010). Due to its usefulness, it is often cultivated in gardens in agricultural communities (Peredo Parada and Barrera Salas, 2016).

The use of *B. globosa* is also recorded among patients attending primary care centers in the city of Esquel, in Argentine Patagonia (Ferreras et al., 2022), by the Mapuche people in different communities, from Curruhuinca (Estomba et al., 2006), from northwest Patagonia in Lake Rosario, Chubut province (Molares and Ladio, 2009), by Mapuche-Tehuelche communities of Nahuelpán, Chubut province (Molares and Ladio, 2014), which shows the continuity with Argentina of its traditional medicinal use. This pattern extends to other countries in the region: in Peru, indigenous communities use *B. globosa* (Silva et al., 2025). For example, a traditional use prevails in the Túpac Amaru settlement, where it is used to treat candidiasis (Salas Ramos and Rosas Rivera, 2023). In Ecuador, the use of aqueous extracts of its leaves for topical treatment of skin conditions has been reported in rural areas of the Babahoyo-Pimocha and Febres-Cordero cantons, in the southern part of the Los Ríos province (Zurita and Gallego, 2017). However, it should not be confused with the Peruvian matico, *Piper aduncum* L. [Piperaceae] (Ore Areche et al., 2021).

Commercially, its leaves are available in supermarkets as a sachet-prepared infusion (Benedetti Ruiz et al., 2009). They are also sold as traditional herbal medicines (THMs) in transparent containers and are commonly distributed as tinctures.

In Chile, no public records exist on trade volumes to assess their magnitude and market impact. Furthermore, it is not included in export figures for the non-timber forest products (NTFPs) segment, understood as goods of biological origin other than wood derived from forests, other wooded land, and trees outside forests, suggesting that its use is mainly in the domestic market (Benedetti Ruiz et al., 2009; Poblete et al., 2025). Various cosmetic and dermocosmetic products have been developed from this species, including shampoos and creams such as Matikomp® and Maticreme® (Knop Laboratories), as well as creams from ANC Laboratories and facial and diaper rash formulations from Apícola del Alba.

The healing properties of *B. globosa* have led to its incorporation into veterinary medicine. Among these products is MATIPET® from DRAG PHARMA, a line for dogs, cats, and other small animals, formulated with natural *B. globosa* extracts and designed to promote healing, reduce inflammation, and provide topical pain relief. This segment also includes products like Cicatrivet® (Veterline). For equine care, formulations such as Kawell® spray, AllGreen Pets' matico coat care spray, and DRAG PHARMA’s Matihorse® are available, demonstrating the diversification of *B. globosa*-based products into animal care and dermocosmetics.

Finally, there is a patent (Letelier and Osandon, 2012) that describes the production of a standardized ethanolic extract of *B. globosa*, designated BG-126. The invention relates to a composition intended to prevent or alleviate gastric pathologies and damage to the digestive system, including ulcers, abdominal pain, dyspepsia, nausea, vomiting, diarrhea, constipation, and inflammation. It also considers its use to mitigate headaches and other symptoms associated with nitrofurantoin or other antimicrobial treatments (Letelier and Osandon, 2012).

## 5 Human health-related bioactivity and toxicity

### 5.1 Antibacterial activity

Ethanolic extracts of *B. globosa* leaves have demonstrated antibacterial activity against *Staphylococcus aureus* and *Escherichia coli* (Pardo et al., 1993). More recently, the hydroalcoholic extract of *B. globosa* leaves showed MICs of 50.0 ± 2.4 and 6.25 ± 0.24 mg/mL against *S. aureus* and *E. coli*, respectively (Hernández et al., 2025). Angoroside A, isolated from the ethanolic leaf extract of *B. globosa*, exhibited antibacterial activity against *S. aureus* (ATCC 25923) at concentrations above 2 mM, as determined by the broth dilution method (Pardo et al., 1997).

The methanolic extract of *B. globosa* leaves was inactive against a panel of pathogenic microorganisms. In antibacterial activity tests, it showed no inhibition against Gram-negative strains such as *E. coli* (ATCC 11229) and *Pseudomonas aeruginosa* (ATCC 9027), nor against Gram-positive strains such as *S. aureus* (ATCC 6538) and *Bacillus subtilis* (ATCC 6633) (Mølgaard et al., 2011).

The essential oil from the leaves of *B. globosa* exhibited an MIC of 25 μg/mL against *Paenibacillus larvae,* permeabilizing and altering the cell and cytoplasmic membranes, thereby causing leakage of cytoplasmic components (Pellegrini et al., 2017).

A standardized *B. globosa* hydroalcoholic extract (BG-126, obtained with 53% ethanol) and its spray-dried (SD) variants were evaluated against *P. aeruginosa*. These included an additive-free formulation (BG-SD) and two variants with solubility enhancers: polyvinylpyrrolidone (BG-SD PVP) and Soluplus® (BG-SD SP) (Araya et al., 2022). In activity tests, BG-SD showed the greatest antimicrobial potency, with a minimum inhibitory concentration (MIC) of 512 μg/mL, surpassing the original BG-126 extract, which had an MIC of 1,024 μg/mL. These results suggest that dry formulations of *B. globosa* maintain or enhance their bacterial inhibitory capacity, positioning them as viable candidates for developing biocompatible topical therapies for wound healing (Araya et al., 2022).

### 5.2 Antifungal activity

The antifungal activity of five terpenes was evaluated, and none inhibited fungal growth at 1000 μg/mL against *Saccharomyces cerevisiae* NCIMB 080178, *Candida albicans* ATCC 10234, *Aspergillus niger* ATCC 16404, *Penicillium notatum* ATCC 11654, *Scopulariopsis brevicaulis* EL 3839, and *Scytalidium dimidiatum*. However, buddledin A and buddledin B showed selective antifungal activity against *Epidermophyton floccosum* SJH, *Trichophyton rubrum* EL 5095, and *Trichophyton interdigitale* EL 5171, with MICs of 43 µM and 51 μM, respectively (Mensah et al., 2000). The methanolic extract of *B. globosa* leaves was inactive against the yeasts and filamentous fungi *C. albicans* (IMI 349010) and *Penicillium expansum* (IMI 285521) in the evaluation of antifungal activity (Mølgaard et al., 2011).

### 5.3 Antiparasitic activity

The trypanocidal potential of the methanolic extract of *B. globosa* and its purified fraction (BG500), enriched with the iridoid 6-*O*-methylcatalpol, was evaluated. The results against *Trypanosoma cruzi* trypomastigotes were less potent than those obtained with the reference drug, nifurtimox (Quintero-Pertuz et al., 2025).

Extracts from the aerial parts of *B. globosa* exhibited significant *in vitro* antiplasmodial activity against *Plasmodium falciparum*, with activity varying by extraction solvent. The methanolic extract (M) was the most active, with an IC_50_ of 6.2 μg/mL. In contrast, the aqueous extract (W) showed lower activity, with an IC_50_ of 8.7 μg/mL (Debenedetti et al., 2002).

### 5.4 Anti-inflammatory activity

The anti-inflammatory activity of *B. globosa* has been primarily attributed to lipophilic extracts and terpenoid constituents that act on the arachidonic acid cascade. In a whole-cell assay using rat peritoneal leukocytes (comprising ∼85% polymorphonuclear cells) by an intraperitoneal injection of 6% oyster glycogen, the CHCl_3_ extract obtained from *B. globosa* roots strongly inhibited both cyclooxygenase (COX) and 5-lipoxygenase (5-LOX) pathways, with 86.7% and 100% inhibition at 50 μg/mL, respectively. This extract yielded several sesquiterpenes, among which buddledin A showed the most relevant activity. Buddledin A inhibited COX with an IC_50_ of 13.7 µM and 5-LOX with an IC_50_ of 50.4 µM, suggesting a dual inhibitory effect on eicosanoid generation. In contrast, buddledins B and C showed only moderate or weak inhibitory activity, whereas dihydrobuddledin A and zerumbone were inactive under the same experimental conditions (Liao et al., 1999).

The anti-inflammatory activity of orally administered *B. globosa* extracts has been evaluated in guinea pigs (*Cavia porcellus* Linnaeus) using the β-carrageenan-induced paw edema model. At 600 mg/kg, the serial methanolic extract showed the greatest efficacy, with 61.4% inhibition, exceeding that of the reference drug, sodium naproxen, at 4 mg/kg. The global methanolic extract showed moderate activity, while the defatted DCM extract showed 43.7% inhibition. In contrast, the hexane extract showed lower oral anti-inflammatory activity, with only 17.2% inhibition (Backhouse et al., 2008b). Topical anti-inflammatory activity was also evaluated in mice using arachidonic acid (AA)- and 12-*O*-tetradecanoylphorbol acetate (TPA)-induced ear edema models at a dose of 3 mg/ear. The defatted DCM extract was the most active in both models, achieving 64.4% inhibition against AA and 83.8% inhibition against TPA. The hexane extract also showed high topical efficacy, particularly in the TPA model, with 79.0% inhibition, compared with 47.7% against AA. The global methanolic extract exhibited moderate, balanced activity, with 32.7% inhibition against AA and 60.3% against TPA. Finally, the methanol-1 extract, despite being the most active by the oral route, showed comparatively lower topical activity, particularly against AA (13.8% inhibition); however, it reached 56.7% inhibition in the TPA model (Backhouse et al., 2008b).

Bioguided fractionation of the defatted DCM extract further supported the contribution of lipophilic constituents to the topical anti-inflammatory effect. The fractions CD4-N and CD5-N reduced TPA-induced inflammation by 78.2% and 83.7%, respectively, at 1 mg/ear. CD4-N contained a mixture of α- and β-amyrins; a steroid mixture composed mainly of β-sitosterol and stigmasterol, together with campesterol, stigmastanol, and ergosterol; and β-sitosterol glucoside, whereas CD5-N contained mainly β-sitosterol glucoside. These findings suggest that sterols, triterpenoids, and their glycosylated derivatives may contribute substantially to the topical anti-inflammatory activity of *B. globosa* (Backhouse et al., 2008b).

Additionally, the ethanolic extract of *B. globosa* leaves has been evaluated for its inhibitory activity against lipoxygenase-15 (LOX-15). In this assay, the extract was considered inactive, with an IC_50_ above 200 μg/mL, whereas baicalein showed an IC_50_ of 25.12 μg/mL (Castro-Saavedra et al., 2016). Taken together, the available evidence indicates that the anti-inflammatory potential of *B. globosa* is more consistently supported by lipophilic extracts and selected terpenoid- or sterol-rich fractions than by crude ethanolic extracts evaluated against LOX-15.

### 5.5 Antinociceptive activity

The antinociceptive activity of *B. globosa* was evaluated using the acetic acid-induced writhing test. The defatted DCM extract was the most active fraction, achieving 76.7% activity, followed by the hexane extract at 41.2% and the methanolic extract at 38.5%. The crude methanolic extract of *B. globosa* showed the lowest effect, at 27.3% (Backhouse et al., 2008b).

In the abdominal contortion test, *B. globosa* extracts showed dose-dependent antinociceptive activity. The summer extract was significantly more active than the autumn extract, with an ED_50_ of 59.5 mg/kg (Backhouse et al., 2008a).

In the tail-flick tests, both oral and topical administration of all extracts induced a dose-dependent antinociceptive effect. The orally administered ethanolic extract had an ED_50_ of 59.0 ± 6.1 mg/kg, while topical application showed an EC25 of 1.1% ± 0.1% (w/v) (Backhouse et al., 2008a).

In the topical formalin assay, all extracts showed very high antinociceptive activity at a single concentration (5%). Notably, the overall methanolic extract (GME) and the summer extract outperformed even ibuprofen, achieving pain-inhibitory values of 85%–90% or higher (Backhouse et al., 2008a).

Various nociceptive antagonists were used to investigate the pharmacological mechanisms underlying the tail-flick model. Prior administration of naltrexone significantly increased the analgesic effect in both extracts, suggesting that the opioid system modulates the action of *B. globosa*. Similar results were observed with naltrindole and nor-binaltorfimine, indicating the specific involvement of delta and kappa-opioid receptors in this modulation. Likewise, pretreatment with tropisetron and prazosin increased the antinociceptive response, demonstrating the involvement of serotonergic (5-HT_3_) and adrenergic (α1) pathways. In contrast, yohimbine and atropine produced only minor changes, ruling out a significant role for α2 adrenergic and muscarinic receptors. Finally, inhibition of nitric oxide synthase (iNOS) by L-NAME significantly increased analgesia, suggesting that the nitric oxide pathway negatively regulates species-induced antinociception. Overall, these results demonstrate that the analgesic effect of *B. globosa* is multimodal, involving complex interactions among the opioid, serotonergic, adrenergic, and nitrergic systems (Backhouse et al., 2008a).

### 5.6 Antioxidant capacity and protective effect against drug-induced oxidative stress

The stem of *B. globosa* has been studied far less than the leaves. However, relevant antioxidant capacity values have been reported for stem extracts, with values of 35,661.7 μmol TE/100 g DW in the ORAC-H assay, 13,404.4 μmol TE/100 g DW in the ABTS assay, and 8,268.5 μmol TE/100 g DW in the DPPH assay (Zamorano-Aguilar et al., 2025). This background suggests that this organ could constitute a complementary source of antioxidant compounds.

Most of the available research on the antioxidant capacity of *B. globosa* has focused on the leaves. In this organ, extracts have shown a high reducing capacity in the FRAP assay (7,492.1 ± 312.7 mg ascorbic acid equivalents/100 g DW), along with consistent values in ABTS (10,475.7 ± 1,105.3 μmol Trolox eq/100 g DW) and DPPH (25,836.6 ± 155.8 μmol Trolox eq/100 g DW). The highest value was observed in the ORAC-H assay (134,147.3 ± 5,792.5 μmol eq. of Trolox/100 g DW), confirming a high overall antioxidant capacity that depends on the method and the radical system evaluated (Zamorano-Aguilar et al., 2020). In the DPPH assay, the hydroalcoholic tincture of *B. globosa* (50 g/275 mL ethanol 70:30, 7 days) exhibited higher antioxidant capacity than the aqueous infusion (5 g/100 mL, 20 min), with EC_50_ values of 0.38 ± 0.01 and 1.59 ± 0.08 µL, respectively (Gastaldi et al., 2016).

From ethanolic extracts prepared from different individuals, moderate antioxidant activity was observed, with IC_50_ values of 60.6 ± 14.8 μg/g in ABTS and 92.1 ± 10.5 μg/g in DPPH, in addition to detectable responses in FRAP (0.74 ± 0.54 mM TE/g) and CUPRAC (1.32 ± 0.80 mM TE/g) (Castro-Saavedra et al., 2016). In the case of aqueous leaf extracts, relevant antioxidant values have been reported in the ORAC-FL (2,175 ± 62 mg/L) and ORAC-PGR (25 ± 1 mg/L) assays (Alarcón et al., 2008), as well as an ORAC-PGR/ORAC-FL ratio of 0.011 (Poblete et al., 2009). These findings are consistent with subsequent studies that reported an ORAC value of 3,720 μmol TE/g (Suwalsky et al., 2017). In addition, *B. globosa* leaf extract has been shown to protect biological targets against oxidative damage in microsomal systems. In a Cu^2+^/ascorbate pro-oxidant model, the extract inhibited microsomal lipid peroxidation, microsomal thiol oxidation, and oxygen consumption, supporting its ability to counteract oxygen-free radical-mediated damage (Letelier et al., 2008). Moreover, a hydroalcoholic leaf extract of *B. globosa* protected hepatic microsomes against oxidative damage induced by oxygen-free radicals (Letelier et al., 2008). However, it did not prevent modifications triggered by the synthetic DPPH radical, suggesting that antioxidant activity measured in chemical assays does not necessarily reflect antioxidant protection in biological systems (Letelier et al., 2008). Furthermore, these extracts have demonstrated concentration-dependent inhibition of microsomal lipid peroxidation, reaching an IC_50_ of 0.13 μL/mg of microsomal protein for the most active extract (Letelier et al., 2012). Additionally, a hydroalcoholic leaf extract of *B. globosa*, characterized by a total polyphenol content of 0.129 mg catechin equivalents/mg of extract, inhibited microsomal glutathione S-transferase (GST) activity involved in drug biotransformation, with inhibition values of 42.4%, 44.2%, and 32.3% for nitrofurantoin, nifurtimox, and acetaminophen, respectively. These findings further support the antioxidant potential of *B. globosa* extracts in the microsomal system (Letelier et al., 2017b).

In contrast, the hydroethanolic (3:7) extracts obtained from leaves and stems did not show relevant activity as xanthine oxidase inhibitors, with IC_50_ values greater than 50 μg/mL (Schmeda-Hirschmann et al., 1992), suggesting that the antioxidant activity of *B. globosa* is not primarily associated with the modulation of this specific enzymatic pathway.

The infusion of *B. globosa* leaves (2 g in 150 mL) exhibited significant antioxidant activity, as demonstrated through complementary experimental approaches. Commercial *B. globosa* tea bags showed a TEAC value of 1.4 mM, lower than black tea (3.2 mM) and boldo (2.15 mM), but comparable to mint (1.35 mM) (Campos and Lissi, 1995). In another TEAC assay, it presented an IC_50_ of 1.8 mL, equivalent to 206 mg of Trolox per cup; in the hypochlorite elimination, an IC_50_ of 1.1 mL (83.5 mg of 5-ASA per cup); and against peroxynitrite, a marked oxidation delay (Δ_time_) of 36.1 min, one of the highest values in the study (Speisky et al., 2006).

*In vitro* studies have shown that the methanolic extract inhibits lipid peroxidation by up to 60% and exhibits an IC_50_ of 8.4 mg/mL in the DPPH assay (Backhouse et al., 2008b). Furthermore, aqueous extracts have demonstrated a protective antioxidant effect in human fibroblasts, primarily attributable to low-molecular-weight compounds that attenuate H_2_O_2_-induced oxidative damage, especially under cell pretreatment conditions (Mensah et al., 2001).

Extracts from *B. globosa* leaves exhibited differential inhibition of oxidative effects generated during the biotransformation of nifurtimox, nitrofurantoin, and acetaminophen in rat liver microsomes. In particular, the hydroalcoholic extract showed a marked protective effect against microsomal lipid peroxidation induced by these compounds (Letelier et al., 2017a). Lipid peroxidation induced by nifurtimox biotransformation was reduced by approximately 65.0% in the presence of the *B. globosa* extract. Similarly, lipid peroxidation associated with nitrofurantoin metabolism decreased by approximately 50%, while lipid damage induced by oxidative metabolism of acetaminophen decreased by 77.3% (Letelier et al., 2017a).

Furthermore, under NADPH-dependent biotransformation conditions, nitrofurantoin, nifurtimox, and acetaminophen inhibited microsomal GST activity by 47.1%, 48.6%, and 47.5%, respectively. The presence of *B. globosa* extract significantly attenuated this inhibition: GST activity increased to 42.4% with nitrofurantoin, 44.2% with nifurtimox, and 32.3% with acetaminophen (Letelier et al., 2017a).

Overall, these findings, which show considerable variability, support the need for further research into the antioxidant activity of *B. globosa*, as it has demonstrated significant antioxidant capacity and efficacy against key reactive oxygen species implicated in human inflammatory processes (Hernández et al., 2025).

### 5.7 Wound healing activity

The wound-healing activity of *B. globosa* is one of the most consistently documented biological properties of the species. It supports its traditional use for treating wounds, ulcers, and skin lesions. The aqueous extract displayed dose-dependent cytoprotective and antioxidant effects against hydrogen peroxide-induced damage in fibroblasts at concentrations ranging from 1 to 5 μg/mL, while higher concentrations (50 μg/mL) resulted in cytotoxicity, suggesting that protection against oxidative stress may contribute to its tissue repair potential (Mensah et al., 1998; 2001). Fractionation of the aqueous extract isolated flavonoids and caffeic acid derivatives, including verbascoside, echinacoside, linarin, luteolin, and 6-hydroxyluteolin, all of which contributed to the antioxidant effect at low concentrations (Mensah et al., 2001).

Additional *in vitro* studies showed that aqueous extracts of fresh *B. globosa* leaves induced keratinocyte differentiation. At concentrations of 50 and 100 μg/mL, the extract significantly increased involucrin expression and enhanced cytokeratin K1 and K10 expression, whereas keratinocyte proliferation increased only slightly, by approximately 6% (Houghton et al., 2005). These findings indicate that the extract’s predominant effect is not mitogenic but instead promotes the maturation and specialization of epidermal cells. The same study also reported changes in the protein expression profile of cultured human dermal fibroblasts and effects on collagen lattice contraction, suggesting possible roles in extracellular matrix remodeling (Houghton et al., 2005).

*In vivo* evidence further supports *B. globosa* wound-healing properties. In a Sprague-Dawley rat model, topical treatment with a standardized hydroalcoholic extract of *B. globosa* accelerated wound closure compared with untreated controls (Letelier et al., 2012). Histological analysis revealed increased fibroblast infiltration and improved extracellular matrix organization in treated tissues. At the molecular level, COX-2 expression peaked earlier in *B. globosa*-treated wounds than in controls, suggesting an earlier inflammatory response followed by faster resolution. Ki-67 expression also showed an earlier proliferative peak, and epidermal thickness analysis confirmed more efficient re-epithelialization in the treated group (Letelier et al., 2012).

Recent studies have expanded the use of *B. globosa* extracts in advanced wound-care biomaterials. Polymeric scaffolds containing the standardized hydroalcoholic extract BG-126 reduced planktonic bacterial growth and biofilm proliferation and improved compatibility with normal human fibroblasts compared with scaffolds without the extract (Ceriani et al., 2025). Further optimization studies using *B. globosa*-loaded scaffolds against dual-species biofilms of *P. aeruginosa* and *S. aureus* showed that intermediate extract concentrations provided the best balance between antimicrobial activity, scaffold structure, wound closure, and re-epithelialization in a murine model of infected wounds (Bahamondez-Canas et al., 2026). More recently, a gelatin–chitosan–hyaluronic acid scaffold enriched with *B. globosa* extract showed a favorable porous architecture, high swelling capacity, fibroblast compatibility, increased secretion of proangiogenic mediators, and enhanced vascularization and dermal tissue organization in a chronic wound model (Ceriani F et al., 2026). Together, these findings indicate that the regenerative potential of *B. globosa* depends not only on extract bioactivity but also on rational formulation design, extract loading, and scaffold physicochemical properties (Ceriani et al., 2025; Bahamondez-Canas et al., 2026).

Electrospun polycaprolactone fibers loaded with extracts of *Gunnera tinctoria* (Molina) Mirb. [Gunneraceae] and *B. globosa* have also been developed for potential applications in treating skin lesions. The best formulation showed favorable morphology, cytocompatibility, and antifungal activity against *C. albicans*; however, it showed no antibacterial activity against *P. aeruginosa* or *S. aureus* (Hermosilla et al., 2026). The specific contribution of *B. globosa* could not be isolated because both plant extracts were combined (Hermosilla et al., 2026).

Overall, the wound-healing activity of *B. globosa* appears to be multifactorial, involving antioxidant protection, modulation of inflammation, support of fibroblast activity, promotion of keratinocyte differentiation, antimicrobial effects, and improved re-epithelialization. These effects may be associated with phenylethanoids such as verbascoside and echinacoside, flavonoids such as linarin and luteolin derivatives, iridoids such as aucubin and catalpol, and lipophilic constituents such as buddledin A and β-sitosterol (Houghton and Mensah, 1999).

### 5.8 Antiplatelet and antithrombotic activity

The ethanolic extract of *B. globosa* leaves showed significant potential in the context of thrombosis, as, at low concentrations and in a dose-dependent manner, it acted as a potent inhibitor of collagen-, convulxin-, and ADP-induced platelet aggregation, with IC_50_ values of 61 μg/mL, 72 μg/mL, and 290 μg/mL, respectively. Furthermore, the percentage of P-selectin-positive platelets decreased. The cellular mechanism underlying this antiplatelet activity may involve inhibition of phospholipase C-γ2 and protein kinase C phosphorylation (Fuentes et al., 2018).

### 5.9 Hypotensive activity

A single dose of the EtOH/H_2_O (7:3) leaf extract of *B. globosa* (i.v., 5 mg/kg) produced a slight decrease in mean arterial pressure of 11.4% ± 1.1%, compared with acetylcholine and with some extracts of the evaluated species (Schmeda-Hirschmann et al., 1992).

### 5.10 Antiproliferative activity

The cytotoxicity of an infusion of the aerial parts of *B. globosa* was evaluated using the MTT assay in the T84 (colon adenocarcinoma) and HTR-8/SVneo (non-tumor trophoblast) cell lines. The results showed EC_50_ values of 1.37 ± 0.17 mg/mL for the T84 tumor cell line and 0.29 ± 0.09 mg/mL for the HTR-8/SVneo cell line. The lyophilized extract did not exhibit selective antiproliferative activity against tumor cells under the evaluated conditions (Gastaldi et al., 2018). In contrast, the ethanolic extract of *B. globosa* leaves was inactive against the MCF-7 and MCF-10A cell lines, with an IC_50_ > 150 μg/mL (Castro-Saavedra et al., 2016).

### 5.11 Antimutagenic activity

Aqueous extracts of *B. globosa* leaves have demonstrated significant antimutagenic activity in the *Drosophila melanogaster* model. Combined treatment with the mutagen ethyl methanesulfonate (EMS) and the plant extract was observed to significantly reduce induced mutations in a concentration-dependent manner, achieving reductions of 39%–55% in small single spots, 42%–71% in large spots, and up to 53% in total mutant spots (Carmona et al., 2016).

### 5.12 Hepatoprotective activity

The hepatoprotective activity of *B. globosa* has been evaluated mainly in relation to its traditional use as an aqueous preparation for liver ailments. Aqueous extracts of Buddleja species, prepared by boiling plant material in water to mimic traditional infusions, were tested in cultured hepatocytes exposed to carbon tetrachloride (CCl_4_), galactosamine (GalN), and complement-mediated cytotoxicity (CMC). In this model, the aqueous extract of *B. globosa* leaves showed inhibitory effects against induced hepatocyte damage, supporting the ethnopharmacological use of the species for liver-related disorders (Houghton and Hikino, 1989).

More recent studies have provided mechanistic support for the hepatoprotective potential of linarin; however, these studies evaluated the isolated compound rather than *B. globosa* extracts. In an *in vitro* NAFLD model using AML12 hepatocytes stimulated with oleic and palmitic acids, and in mice fed a high-fat diet, linarin reduced lipid accumulation, improved serum lipid and liver injury markers, enhanced autophagy, suppressed inflammatory mediators, and modulated gut microbiota composition. These effects were associated with inhibition of PI3K/Akt/mTOR pathway phosphorylation and activation of autophagy-dependent mechanisms (Lv et al., 2025; Xiao et al., 2025). In another study, linarin attenuated high-fat diet-induced hepatic steatosis in C57BL/6J mice and reduced lipid accumulation in oleic/palmitic acid-stimulated HepG2 cells, mouse primary hepatocytes, and AML12 cells. This effect was associated with reduced hepatic fat deposition, improved liver function, decreased oxidative stress, and enhanced mitochondrial function. Mechanistically, linarin was proposed to inhibit PDE4D activity and activate the cAMP/PKA/CREB pathway, thereby improving lipid metabolism and reducing oxidative and ferroptosis-related injury (Lv et al., 2025; Xiao et al., 2025).

Available evidence supports the hepatoprotective potential of B. globosa based on early *in vitro* studies using aqueous leaf extracts and isolated constituents, particularly linarin and phenylpropanoid glycosides. Nevertheless, the recent mechanistic evidence derives from studies of purified linarin, and further studies using standardized *B. globosa* extracts are required to determine whether these mechanisms can be directly attributed to the whole-plant preparation.

### 5.13 Inhibitory activity of β-glucuronidase

The search for β-glucuronidase inhibitors aims to reduce drug toxicity, inflammation, and cancer-related processes, particularly in the gut (Awolade et al., 2020). In this assay, ethanolic/aqueous (EtOH/H_2_O, 7:3) extracts of *B. globosa* leaves showed an IC_50_ of 12 μg/mL. In comparison, stem bark extracts showed an IC_50_ of 9 μg/mL, demonstrating relevant inhibitory activity (Schmeda-Hirschmann et al., 1992).

## 6 Non-human health-related bioactivity, safety, and toxicological profile

### 6.1 Cytotoxicity

The aqueous extract of *B. globosa* leaves at 10 μg/mL showed no cytotoxicity in normal human keratinocytes (NHK); however, mild cytotoxicity was observed in the HaCaT keratinocyte cell line, which the authors considered non-toxic (Houghton et al., 2005).

HaCaT cells were treated with various concentrations of verbascoside, which decreased viability at 100 μg/mL and above (de Moura Sperotto et al., 2018).

In safety assessments in fibroblasts and HaCaT cells, verbascoside at 150 μg/mL was non-cytotoxic and prevented photodamage (Kanlayavattanakul et al., 2024).

### 6.2 Hemolytic activity

The aqueous extract of *B. globosa* leaves induced moderate hemolysis (≈20%) even at high concentrations, suggesting low cytotoxicity to human erythrocytes (Suwalsky et al., 2017). In rats, administration of a standardized alcoholic extract of *B. globosa* leaves did not alter hematological parameters, which remained stable within physiological ranges and showed no significant differences compared to the control group (Letelier et al., 2012). Most clinical chemistry indicators, including biomarkers of renal function and glucose metabolism, showed no alterations, confirming high systemic tolerance to the treatment (Letelier et al., 2012).

### 6.3 Insecticidal activity

The insecticidal effect of *B. globosa* powder was evaluated against the maize weevil (*Sitophilus zeamais* Motschulsky). However, only a 23.7% mortality rate was observed, making the results for *B. globosa* the least promising compared with the control and the powders of the two other species evaluated (Zurita Vásquez et al., 2017). The essential oil of *B. globosa* exhibited significant repellent activity against *Rhyzopertha dominica*, a beetle considered an important pest of stored agricultural products (Parichanon et al., 2025).

### 6.4 Nematicidal activity

The incorporation of dried *B. globosa* leaves as an organic amendment at a low concentration (0.5%; 2 g of dried leaf tissue) reduced the number of *Meloidogyne hapla* eggs by approximately 30% and decreased the presence of second-stage juveniles (J2) sixfold compared with the untreated control (Böhm et al., 2009).

### 6.5 Mutagenic activity

The concentrations of *B. globosa* extracts used in the mutagenic experiments with *D. melanogaster* ranged from 3.83 to 15.32 mg/mL. The results indicate that no concentration of *B. globosa* administered by ingestion significantly increased the frequency of mutant spots, supporting the validity of the negative results obtained (Carmona et al., 2016).

### 6.6 Toxicity

The *B. globosa* infusion showed a median lethal concentration (LC_50_) of 17.20 ± 2.55 mg/mL in the *Artemia salina* bioassay. Although this value is within the range analyzed in the study, it is significantly above the established toxicological risk threshold of 1 mg/mL. These results suggest that the extract is safe for consumption at the concentrations evaluated. However, further chronic studies are recommended to determine the safety profile and long-term effects of consumption (Gastaldi et al., 2018).

In another study, the toxicity of crude *B. globosa* extracts was evaluated using a bioassay with *A. salina*. Extracts obtained using an ethanol/water (7:3) mixture showed LC_50_ values of 2300 μg/mL for leaves and 465 μg/mL for stems. These values suggest that the stem components are more toxic than the leaf extracts (Schmeda-Hirschmann et al., 1992).

The leaf extract of *B. globosa*, especially at high doses, alters the reproductive cycle and significantly reduces ovulation in rats. This phenomenon is attributed, at least in part, to the ability of both verbascoside and the total extract to physically bind to estrogen receptors, thereby competing with natural hormones. However, because considerably higher concentrations than those of estradiol are required to displace the hormone from the receptor, its effect is mild at low doses; nevertheless, caution should be exercised with very high or concentrated consumption, given its potential antiestrogenic character (Parada et al., 2014).

In the case of the aqueous extract of *B. globosa* flowers, the results show a moderate and stable cytotoxic effect in the Chinese hamster ovary cell line (CHO-K1) at concentrations of 50 μg/mL or greater. The neutral red assay showed cell viability close to 80%, indicating limited impairment of cytoplasmic membrane integrity, with an approximate 20% reduction. In contrast, the MTT assay indicated approximately 60% viability, suggesting a 40% decrease in cellular metabolic capacity. These results indicate that the effect of *B. globosa* extract on normal cells is largely independent of the concentration evaluated and that, in all cases, cell viability remained above 50%, suggesting low to moderate cytotoxicity (Zamorano-Aguilar et al., 2020).

## 7 Pharmacokinetic properties of phytochemicals of *B. globosa*

The pharmacokinetic properties of plant secondary metabolites can provide valuable insights into the biological activities traditionally attributed to medicinal plants. In this context, the ADME properties of metabolites reported in *B. globosa* were predicted using the SwissADME web platform. The predicted parameters are summarized in Supplementary Table S3 (see Supplementary Material) and discussed below in relation to the pharmacological and ethnomedicinal uses of this species.

One of the ethnopharmacological properties reported for *B. globosa* is its ability to promote tissue repair (Mellado Gutiérrez, 1923; Oyarzo, 2024), which has been associated with the presence of verbascoside (Houghton, 1989; 1995; 2003; Houghton and Mensah, 1999; Liao et al., 1999). Structurally, this compound is a phenylpropanoid derivative linked to two sugar moieties (glucose and rhamnose), which strongly enhances its water solubility (LogS = −2.87) while limiting skin permeability (LogKp = −10.46). These properties favor its accumulation on the dermal surface, which may help explain its ethnopharmacological use as a wound-healing agent. Similar physicochemical characteristics are shared by other metabolites identified in this species, including isoverbascoside, forsythoside A, forsythoside B, campneoside I, lipedoside A, eukovoside, and martynoside (Supplementary Table S3).

Other ethnopharmacological uses involve the digestive system, where infusions of this plant are traditionally recommended for treating simple gastric ulcers, chronic dysentery, and intestinal catarrh. Additionally, hepatoprotective effects have been reported (Mellado Gutiérrez, 1923; Muñoz et al., 1981). These traditional uses may be partially associated with water solubility (LogS), gastrointestinal absorption (GI), and predicted interactions with cytochrome P450 (CYP) enzymes. Among the metabolites reported in this species, several flavonoids, phenolic acids, and phenylpropanoids exhibit favorable water solubility (LogS between −2.0 and −4.0) together with high gastrointestinal absorption, including luteolin, apigenin, pinocembrin, quercetin, chrysin, catechin, salicylic acid, syringic acid, gallic acid, caffeic acid, ferulic acid, chlorogenic acid, and *p*-coumaric acid, among others (Supplementary Table S3). Although many flavonoids are predicted to act as substrates of certain CYP enzymes (Supplementary Table S3), several phenolic acids and phenylpropanoids are predicted not to be substrates of these enzymes. Therefore, the hepatoprotective activity of hydroalcoholic extracts of *B. globosa* may be associated with the direct antioxidant effects of these compounds and their potential regulation of endogenous antioxidant systems (Houghton and Hikino, 1989). In addition, some flavonoids have demonstrated relevant hepatoprotective effects in cellular and murine models (Lv et al., 2025; Xiao et al., 2025).

Another pharmacological property reported for *B. globosa*, which may relate to its hepatoprotective effects, is inhibition of β-glucuronidase, an enzyme associated with inflammatory processes and cancer development, particularly in the intestinal tract (Awolade et al., 2020). In this context, the water solubility and gastrointestinal absorption properties of the flavonoids, phenolic acids, and phenylpropanoids described above may play a relevant role in this pharmacological activity. For instance, lineranin, a flavonoid reported in this species, has been associated with hepatoprotective effects in both cellular and murine models (Lv et al., 2025; Xiao et al., 2025).

Another biological activity derived from the ethnopharmacological use of this plant for wound washing is its antimicrobial activity. In this case, polar extracts of *B. globosa* did not show activity against *S. aureus*, *B. subtilis*, *E. coli*, or *P. aeruginosa* (Pardo et al., 1993; Mølgaard et al., 2011). However, the essential oil, rich in terpenes, showed a MIC of 25 μg/mL against *P. larvae* (Pellegrini et al., 2017). This suggests that the relatively high lipophilicity of these metabolites (iLogP >2.0, Supplementary Table S3) facilitates their passage across bacterial cell membranes. Indeed, the essential oil was reported to disrupt the cell wall and cytoplasmic membrane, leading to leakage of cytoplasmic components (Pellegrini et al., 2017).

As an extension of the antimicrobial evaluation, the antiparasitic activity of *B. globosa* extracts has also been investigated. In particular, activity has been reported against *P. falciparum* and *Trypanosoma cruzi*, with the methanolic extract showing the highest activity (Debenedetti et al., 2002; Quintero-Pertuz et al., 2025). These results suggest that the active metabolites may possess partial water solubility (LogS between −1.0 and −2.0) combined with moderate lipophilicity (LogP between 1.0 and 2.0), which favors their ability to cross biological membranes. In this context, Supplementary Table S3 indicates that several iridoids, including aucubin, catalpol, *p*-methoxycinnamoyl-aucubin, *p*-methoxycinnamoyl-catalpol, catalposide, and 6-*O*-methylcatalpol, display these properties and could therefore contribute to the observed antiparasitic activity. Notably, 6-*O*-methylcatalpol has been identified as an active compound against *T. cruzi* trypomastigotes (Quintero-Pertuz et al., 2025).

Finally, hypotensive activity has been observed in animal (rat) models, while antiplatelet effects have been demonstrated in *in vitro/ex vivo* platelet assays with *B. globosa* extracts (Schmeda-Hirschmann et al., 1992; Fuentes et al., 2018). These biological effects are associated with the circulatory system, suggesting that the metabolites involved should present adequate water solubility (LogS > −4.0) and high gastrointestinal absorption to ensure systemic bioavailability in the vascular compartment. Several phenolic acids, phenylpropanoids, phenylethanoids, and flavonoids reported in *B. globosa* meet these criteria (Supplementary Table S3). However, an additional factor to consider is their interaction with membrane transport systems, such as P-glycoprotein, which may influence intestinal absorption and systemic exposure, thereby contributing to the observed pharmacological effects.

In contrast, the wound-healing properties traditionally associated with the topical application of compresses prepared from this species (Mellado Gutiérrez, 1923; Muñoz et al., 1981) may be linked to interactions between more lipophilic metabolites and dermal cell membranes. In this regard, compounds such as luteolin (1), apigenin (8), and buddledin A (100) may exhibit greater membrane affinity and enhanced local retention, thereby contributing to the cicatrizing properties attributed to *B. globosa* (Supplementary Figure S1).

The BOILED-Egg model did not accommodate acteoside/verbascoside (39) and echinacoside (40), as their topological polar surface area (TPSA) values fell outside the model’s defined scale. This behavior indicates a highly polar character and pronounced hydrophilicity, consistent with their high aqueous solubility. These physicochemical properties may support the ethnopharmacological use of *B. globosa* decoctions for wound cleansing, as described by Oyarzo (2024).

Within the same framework, luteolin, luteolin 7-*O*-glucoside, apigenin 7-*O*-glucoside, and apigenin were positioned outside the yolk and, in some cases, beyond the BOILED-Egg model boundaries, which is likewise consistent with their hydrophilic nature. Traditionally, infusions of this species have been used to treat hepatic disorders (Mellado Gutiérrez, 1923; Muñoz et al., 1981), a use that may be associated with the potential for gastrointestinal absorption of these metabolites. Notably, these compounds were predicted to be non-substrates of P-glycoprotein (PGP−), suggesting a reduced likelihood of intestinal efflux and, consequently, potentially greater systemic exposure (Supplementary Figure S1).

Overall, the predicted ADME properties of metabolites reported in *B. globosa* suggest that their physicochemical characteristics may contribute to the diverse pharmacological activities traditionally attributed to this species. Compounds displaying high water solubility and favorable gastrointestinal absorption may account for the systemic effects associated with vascular and hepatoprotective activities. Conversely, metabolites with moderate lipophilicity could facilitate membrane permeability and intracellular access to microbial or parasitic targets. These pharmacokinetic predictions provide a mechanistic framework for rationalizing the ethnopharmacological applications of *B. globosa* and support further experimental studies to validate the biological relevance of these metabolites.

In addition, structural alerts related to assay interference were assessed to better understand the pharmacological significance of these compounds. PAINS alerts were found for twenty-eight of the phenolic compounds examined (catechol alerts) (Supplementary Table S4). Although these molecules tend to react non-specifically and generate false positives in high-throughput screening, the presence of a PAINS core indicates a likelihood of problematic behavior but does not mean the compound is a false positive in 100% of cases.

## 8 Pharmacological effects of the main phytochemical of *Buddleja globosa*: verbascoside

Verbascoside, also known as acteoside, kusaginin, or orobanquin (Marčetić et al., 2025), is a water-soluble phenylethanoid ester composed of four structural subunits: caffeic acid, glucose, rhamnose, and phenylethyl alcohol (Alipieva et al., 2014). This compound was first isolated from *Verbascum thapsus* (Scarpati and Delle Monache, 1963) and is widely distributed in medicinal species (Pongkitwitoon et al., 2024).

In particular, high concentrations of verbascoside have been reported in certain species, such as *Barleria prionitis* L., *Barleria lupulina* Lindl., *Rhinacanthus nasutus* (L.) Kurz, *Orthosiphon aristatus* (Blume) Miq., and *Nicoteba betonica* (L.) Lindau (Mohammed et al., 2025). In *B. globosa*, verbascoside was first documented in leaves more than 3 decades ago and has since become a key bioactive marker (Pardo et al., 1993). Verbascoside has also been identified in various species of the genus *Buddleja*, including *B. davidii* (Fan et al., 2008), *Buddleja officinalis* Maxim. (Xie et al., 2020), *Buddleja salviifolia* (L.) Lam. (Pendota et al., 2013), *Buddleja brasiliensis* J.Jacq. ex Spreng. (Filho et al., 2012), *Buddleja cordata* Kunth (Arano-Varela et al., 2020), *Buddleja saligna* Willd. (Nina et al., 2024), *B. scordioides* (Avila Acevedo et al., 2005), *B. polystachya* (Al Ati et al., 2015), *B. perfoliata* (García-Bores et al., 2013), *Buddleja thyrsoides* Lam. (Mahlke et al., 2009), and *Buddleja cordobensis* Griseb. (Garro et al., 2021), suggesting chemotaxonomic relevance within the genus.

This secondary metabolite has attracted increasing scientific interest due to its broad spectrum of biological activities. Recent studies have shown that verbascoside has antioxidant and anti-inflammatory properties and can modulate processes involved in tissue regeneration (De Moura Sperotto et al., 2018). These advances have prompted the publication of numerous reviews since 2020, focusing on its therapeutic potential and the molecular mechanisms underlying its biological activity (Nigro et al., 2020; Zhou et al., 2020; Khalaf et al., 2021; Khan et al., 2022; Xiao et al., 2022; Rossi et al., 2023; Saha et al., 2024; Marčetić et al., 2025; Mohammed et al., 2025; Szkudlarek et al., 2025; Zhou, 2025; Rao et al., 2026; Zhang et al., 2026).

### 8.1 Antihyperalgesic activity

Verbascoside at a dose of 100 mg/kg reversed mechanical hyperalgesia in rats subjected to chronic constriction injury (CCI) of the sciatic nerve, a model of neuropathic pain, and in those injected with sodium monoiodoacetate (MIA), a model of osteoarthritis/chronic osteoarticular pain, without affecting nociceptive thresholds in untreated animals, indicating a disease-modifying effect rather than a classic analgesic effect (Isacchi et al., 2011).

### 8.2 Anti-inflammatory activity and attenuation of atopic dermatitis

In this study, an inflammatory state was induced by treating THP-1 cells (human myelomonocytic leukemia cells) with pro-inflammatory stimuli, such as LPS and IFN-γ. This treatment significantly decreased iNOS expression and activity, extracellular O_2_ production, and SOD, CAT, and GPx activity (Speranza et al., 2010). On the other hand, verbascoside showed a significant inhibitory effect on carrageenan-induced hind paw edema in mice (19.3%–26.9%) (Akdemir et al., 2011). Verbascoside also significantly attenuated overall symptoms of atopic dermatitis, scratching behavior, and skin lesion severity in the 2,4-dinitrochlorobenzene (DNCB)-induced murine model. At the systemic level, verbascoside significantly reduced serum IgE levels and Th2-associated cytokines, thereby suppressing the sensitization-induced Th2 immune response (Li et al., 2018). Locally, at the site of the skin lesion, verbascoside treatment inhibited the expression of the proinflammatory cytokines TNF-α, IL-6, and IL-4, which correlated with the observed histopathological improvement (Li et al., 2018).

Additionally, in a human monocyte THP-1 *in vitro* model, verbascoside dose-dependently suppressed DNCB-mediated induction of the surface molecules CD86 and CD54, which are involved in T-cell activation. Furthermore, it reduced TNF-α and IL-6 secretion and blocked activation of the NF-κB signaling pathway (Li et al., 2018).

### 8.3 Antinociceptive activity

The antinociceptive activity of verbascoside was evaluated against *p*-benzoquinone-induced contortions in mice; at 100 mg/kg, it inhibited contortions by 23.2% (Küpeli et al., 2007). In another study, verbascoside at 200 mg/kg exhibited 32.4% antinociceptive activity in the *p*-benzoquinone-induced contortion model in mice (Akdemir et al., 2011).

### 8.4 Multimodal modulatory effect of neuroprotection, inflammation, and oxidative stress in a spinal cord injury model

Administration of verbascoside at an optimal dose of 60 mg/kg demonstrated superior efficacy in functional and molecular recovery following spinal cord injury (SCI), achieving a BBB score of 8.00 by the third week, indicative of increased joint mobility and the initiation of walking. At the cellular level, this compound acts as a potent multimodal modulator: it protects neuronal integrity by increasing MAP-2 levels and enhancing autophagy via MAP1LC3A, while reducing glial scar density by decreasing GFAP protein expression. Its neuroprotective capacity is based on critical regulation of apoptosis, whereby it reduces Caspase-3 to 28.8 pmol/L, decreases the pro-apoptotic marker Bax to less than half, and triples the level of the protective protein Bcl-2. Furthermore, it significantly mitigates the inflammatory response by inhibiting iNOS and reducing key cytokines, including TNF-α (from 3365 to 946.3 pg/mL) and IL-1β. It also neutralizes ROS and reduces MDA levels, thereby protecting the cell membrane from lipid peroxidation. This effect is enhanced by stabilizing the Nrf2/Keap1 genetic defense system, thereby restoring the cell’s endogenous antioxidant capacity. Finally, molecular docking studies confirm a strong binding affinity of verbascoside for the proteins Casp3 (−8.5 kcal/mol), TNF-α, and MAP1LC3A (−8.3 kcal/mol), physically validating its ability to intervene in the mechanisms of cell death, inflammation, and recycling (Ning et al., 2024).

### 8.5 Wound healing activity

In accordance with the traditional uses attributed to plant species rich in verbascoside, the healing activity of this compound has become a priority focus of biotechnological research (Houghton and Hikino, 1989; Houghton, 1995; 2003; Houghton and Mensah, 1999; Liao et al., 1999). The scientific validation of this ancestral knowledge seeks to elucidate the molecular mechanisms by which verbascoside promotes tissue repair and skin regeneration (Akdemir et al., 2011; Kanlayavattanakul et al., 2024). In this context, species such as *Acanthus ebracteatus* Vahl., rich in verbascoside, have been proposed as promising candidates for developing innovative skin care products, supported by consistent experimental evidence (Kanlayavattanakul et al., 2024). In UVB-irradiated HaCaT cells (human immortalized keratinocytes), the use of verbascoside in combination with oleuropein and hydroxytyrosol (O/H/V) attenuated collagen degradation and reduced the expression of pro-inflammatory factors, an effect associated with the upregulation of Nrf2. In the HaCaT-HDF co-culture system, the treatment further led to a significant decrease in the expression of genes related to collagen degradation and pro-inflammatory mediators in HDF cells (Wang et al., 2025).

*In vitro*, using various assays (Mensah et al., 2001; Houghton et al., 2005; Korkina et al., 2007; Kanlayavattanakul et al., 2024) with HaCaT keratinocytes, verbascoside significantly improves wound closure at concentrations between 1 and 10 µM and stimulates fibroblast and keratinocyte migration (Crivellari et al., 2018). It stimulates wound closure in HaCaT keratinocytes; at all concentrations tested, a significant increase in the rate of wound closure was observed. The best results were observed at concentrations of 5 and 10 μg/mL, with closure percentages of 58.7% and 57.7%, respectively, after 48 h of culture (De Moura Sperotto et al., 2018). In another more recent study, verbascoside at 20 μg/mL significantly improved wound closure in fibroblasts and HaCaT cells compared to the control (Kanlayavattanakul et al., 2024).

In normal human dermal fibroblasts (NHDFs), verbascoside administration is associated with increased expression of MT1-MMP, a membrane metalloproteinase that activates proMMP-2 on the cell surface. Thus, verbascoside does not act at the transcriptional level of MMP-2 but rather modulates its post-translational activation via MT1-MMP. Furthermore, the conversion of proMMP-2 (the inactive form) to its active form is enhanced in a dose- and time-dependent manner via the PI3K pathway, even under IL-1β- and TNF-α-induced inflammatory conditions (Si et al., 2018).

Verbascoside increases Rab31 protein expression, a direct regulator of the proliferation, migration, and differentiation of hair follicle stem cells (HFSCs), thereby enabling their activation from a resting state and promoting re-epithelialization after injury. This demonstrates that Rab31 overexpression enhances these regenerative processes, while its silencing inhibits them, and that verbascoside’s pro-healing effects depend directly on this pathway. In addition, *in vivo* studies have shown that hydrogels loaded with verbascoside significantly accelerate wound closure and skin regeneration (Liu et al., 2025).

*In vivo* models show that verbascoside treatment promotes near-complete healing of skin wounds after 14 days of observation (Crivellari et al., 2018). Pure verbascoside achieved 40.52% wound closure by day 12. Analysis with HE and Van Gieson stains confirmed that verbascoside accelerates re-epithelialization (formation of new skin) and improves tissue remodeling, more rapidly passing through the phases of inflammation and proliferation (Akdemir et al., 2011).

### 8.6 Pharmacokinetic efficacy of liposomal verbascoside in models of chronic inflammatory and neuropathic pain

Advanced delivery systems, such as liposomes, enhance the therapeutic efficacy of verbascoside by stabilizing it and preventing hydrolysis. In chronic pain models of inflammatory (MIA) and neuropathic (CCI) origin, the liposomal formulation at 100 mg/kg demonstrated superior pharmacokinetics compared with the free solution, extending the antihyperalgesic effect for up to 60 min after administration (Isacchi et al., 2016).

### 8.7 Cardioprotective effects in myocardial ischemia-reperfusion injury

Results show that pre-administration of verbascoside exerts a clear cardioprotective effect in rats with myocardial ischemia-reperfusion injury (MIRI). The treatment reduced infarct size, improved electrocardiographic abnormalities, and attenuated myocardial histological damage. At the biochemical level, serum biomarkers of cardiac injury, including cTnI and CK-MB, decreased significantly (Li et al., 2025a).

Furthermore, verbascoside reduced oxidative stress, as evidenced by decreased MDA and ROS levels and increased SOD activity (Li et al., 2025b). In parallel, it inhibited cardiomyocyte apoptosis by modulating key proteins in the apoptotic pathway (increasing Bcl-2 and decreasing Bax and caspase-3), thus promoting cell survival (Li et al., 2025a; 2025b). Mechanistically, the results suggest that verbascoside’s cardioprotective effect is mediated by activation of the HSP90AA1/PI3K/Akt/GSK-3β signaling pathway, a key regulator of cell survival, apoptosis inhibition, and the oxidative stress response (Li et al., 2025a).

Intraperitoneal administration of verbascoside (20 mg/kg) protected the myocardium after heart transplantation by inhibiting ferroptosis, reducing cardiac injury biomarkers, and attenuating structural damage to myocardial tissue, possibly through activation of the GDF15/GPX4/SLC7A11 pathway (Zhang et al., 2025). Therefore, verbascoside is postulated to activate the SLC7A11/GPX4 antiferroptotic axis, suggesting that modulation of ferroptosis is a central, shared mechanism underlying its cytoprotective effect (Zhang et al., 2026).

### 8.8 Pharmacokinetics of verbascoside

A critical parameter to consider is verbascoside’s limited oral absorption. Its rapid metabolism and systemic excretion contribute to very low bioavailability. This makes it difficult for the compound to reach therapeutically effective plasma concentrations, thereby limiting its biological activity. Furthermore, as with many glycosides, verbascoside can be hydrolyzed in the gastrointestinal tract by digestive enzymes or by the gut microbiota before absorption, further reducing the fraction of intact compound available systemically (Xiao et al., 2022).

### 8.9 Safety of verbascoside

Verbascoside has shown a favorable safety profile in normal cells *in vitro*, with no cytotoxicity in human HepG2 hepatocytes or human embryonic kidney cells (HEK293) at concentrations >200 μM, with high viability (Shi et al., 2022). Similarly, another study found no cytotoxic effects in HepG2 or NIH cells at high concentrations (Etemad et al., 2015).

In primary human lymphocytes, verbascoside did not induce cytotoxicity or significantly increase apoptosis at 1.25–160 μM; at 80 μM, it even promoted increased cell proliferation (Song et al., 2016). In normal human IMR90 lung fibroblasts, prolonged exposure (3 weeks) to 100 μM did not produce toxicity; instead, it stimulated cell growth and delayed senescence (Cheimonidi et al., 2018). In normal human MeT-5A mesothelial cells, verbascoside showed no cytotoxic effects across a wide concentration range (10–500 μM) (Jaramillo-Morales et al., 2023). At 100 μg/mL, verbascoside showed no toxic effects on peripheral blood mononuclear cells (PBMCs) or normal prostatic epithelial cells (HPrECs). Similarly, in human non-tumor hepatocytes (THLE-3) exposed to 50 μg/mL, the cells maintained their full viability, with no evidence of apoptosis or necrosis (Kaewdaungdee et al., 2025). From a genetic standpoint, verbascoside has been confirmed as a non-genotoxic compound, as DNA damage assays showed no nuclear material fragmentation (Kaewdaungdee et al., 2025).

Analysis of cytochrome P450 (CYP) isoenzyme expression in THLE-3 cells treated with verbascoside (50 μg/mL) reveals a favorable metabolic biosafety profile, albeit with a specific pharmacological restriction. On the one hand, the stability observed in CYP3A4 (82.87%) is a key safety indicator, as this enzyme metabolizes approximately 50% of conventional drugs; the lack of difference compared to the control suggests that the compound would not interfere with most current medical treatments. This neutrality extends to CYP1A2 (82.23%), CYP2A6 (90.14%), and CYP2E1 (84.63%), implying that verbascoside does not impair the liver’s ability to metabolize alcohol or environmental toxins. However, CYP2D6 expression was reduced to 38.38%, below half its normal level. Given that this enzyme is responsible for the metabolism of 25% of clinical drugs, including opioid analgesics, psychotropic drugs, and beta-blockers, this finding highlights the risk of drug-nutrient interactions that could increase systemic toxicity or reduce the therapeutic efficacy of such drugs if administered concomitantly with high doses of verbascoside (Kaewdaungdee et al., 2025).

The safety of verbascoside has been evaluated using the rota-rod test in male Sprague-Dawley rats. At the highest doses tested (up to 600 mg/kg), the compound did not alter the animals' overall behavior. The number of falls indicated no impairment of motor coordination. Furthermore, during each experimental session, the number of falls progressively decreased, demonstrating intact locomotor activity and normal motor learning (Isacchi et al., 2011).

Verbascoside showed a high safety profile in animal models: a single intraperitoneal injection was administered to mice at doses up to 5 g/kg to assess acute toxicity; the dose did not reach the LD_50_, indicating a very wide safety margin. In subacute toxicity, after 21 days of administration, no deaths or visible signs of toxicity were observed. Furthermore, hematological, biochemical, and pathological parameters did not differ significantly from those of the control group (Etemad et al., 2015).

## 9 Clinical trials

### 9.1 Clinical trials of *Buddleja globosa*

Until 2011, the main scientific evidence supporting and validating the traditional uses of *B. globosa* came primarily from cell-free studies, *in vitro* models, and murine models, with no clinical trials in humans (Houghton and Hikino, 1989; Pastene-Navarrete and Torres-Vega, 2021).

To date, four clinical studies have been conducted in Chile with *B. globosa*. The first focused on treating palmar-plantar erythrodysesthesia (PPE) in patients undergoing chemotherapy. This study evaluated 207 chemotherapy regimens in 121 patients, totaling 1262 treatment cycles. In all regimens, patients received the best available supportive care. Additionally, in 46 regimens, patients received a lotion formulated with standardized *B. globosa* extract, while in 43 regimens, they received a lotion without the extract (placebo). Of the three groups evaluated, those receiving the best supportive care plus *B. globosa* showed the greatest clinical benefit, with a statistically significant difference (Rubio Lara, 2011).

The second study focused on preventing radioepithelitis in patients undergoing radiotherapy. This trial enrolled 78 patients; 43 received a gel formulated with standardized *B. globosa* extract as prophylaxis, while 35 received a placebo gel. 16.2% of the patients treated with *B. globosa* did not develop radioepithelitis, compared to 8.5% in the placebo group. Furthermore, 53.5% of patients in the *B. globosa* group experienced grade 1 toxicity, compared with 25% in the placebo group. Grade 2 toxicity was observed in 27.9% of the patients treated with *B. globosa* and in 57.1% of the placebo group. Finally, only 2.3% of patients in the *B. globosa* group experienced grade 3 toxicity, compared with 8.5% in the placebo group. These results indicate that patients treated with *B. globosa* experienced less severe toxicity and a longer delay than those who received a placebo (Rubio Lara, 2011).

The third clinical study evaluated BG126®, a standardized hydroalcoholic extract of *B. globosa* leaves. The objective of this trial was to assess the effect of the BG126® extract on nitrofurantoin (NFT)-induced gastrointestinal adverse effects, a widely used antimicrobial for the treatment of lower urinary tract infections that frequently causes undesirable gastrointestinal effects (Letelier et al., 2017a). For this study, 56 patients were randomized to receive NFT in combination with the standardized *B. globosa* extract BG126® (*n* = 28) or placebo (*n* = 28) for 10 days. By the 10th day of treatment, patients receiving BG126® showed a 47% reduction in the incidence of nausea and a lower frequency of abdominal pain compared with the control group. At the end of the protocol, the group treated with BG126® had negative urine cultures, demonstrating superior therapeutic efficacy and better gastric tolerance compared to the placebo group (Letelier et al., 2017a).

A clinical trial by Bustamante et al. on wound care with *B. globosa* cream is registered; however, at the time of this review, the results were not yet available (Bustamante et al., 2014).

### 9.2 Clinical trials of verbascoside and verbascoside-rich plants

*In vitro* biosafety findings strengthen verbascoside’s profile for therapeutic applications and highlight the need for further human validation. Historically, there have been few robust clinical studies on this phenylethanoid glycoside. However, this trend is shifting toward translational medicine, as seen in studies with verbascoside-rich plants (Xiao et al., 2022), as well as in the registered clinical trial titled “Validity and Safety of Reh-acteoside Therapy for Patients of IgA Nephropathy” (Deng, 2016) and in the study with the pure compound (Campo et al., 2015).

The only study with verbascoside is a randomized, single-center, double-masked, placebo-controlled phase II trial designed to evaluate the efficacy and tolerability of verbascoside in modulating platelet aggregation (PA) values in subjects with cardiovascular (CV) risk factors. One hundred adult participants with at least one CV risk factor were randomly assigned to placebo, verbascoside 50 mg, or verbascoside 100 mg for 2 weeks. Following 2 weeks of treatment with verbascoside at the 100 mg dose, PA values decreased significantly with both arachidonic acid (AA) stimulation (from 51% ± 13% to 39% ± 15%, *p* < 0.01) and adenosine diphosphate (ADP) stimulation (from 60% ± 12% to 49% ± 15%, *p* = 0.01). However, aggregation values did not change significantly in the verbascoside 50 mg group or the placebo group. No serious adverse events were reported during the study, and no subjects withdrew due to such events (Campo et al., 2015).

Information exists regarding trials with verbascoside-rich extracts, as this compound is included in commercial products. One example is Mucosyte®, a concentrated solution based on a standardized extract of *Syringa vulgaris* L. [Oleaceae] containing verbascoside, formulated as an oral mucosa protectant. This product has been used in the management of oropharyngeal lesions associated with chemotherapy-induced oral mucositis, a frequent and painful complication in children with leukemia. This condition results from chemotherapy-induced cytotoxic damage to the oral mucosal epithelium, leading to inflammation, ulceration, and severe pain, thereby impairing basic functions such as eating and speaking (Bardellini et al., 2016).

The leaves of *Aloysia polystachya* (Griseb.) Moldenke [Verbenaceae] are traditionally used in southern Brazil to relieve anxiety symptoms. In this study, adults with anxiety symptoms received capsules containing *A. polystachya* leaf powder or a placebo for 8 weeks (Carmona et al., 2019). This was a randomized, double-masked, placebo-controlled, phase II clinical trial. Fifty-four adults with self-reported anxiety symptoms were randomly assigned to receive capsules containing *A. polystachya* leaf powder (300 mg twice daily; standardized to 115.7 ± 1.9 μg/mg verbascoside) or a placebo (maltodextrin) for 8 weeks. Treatment with *A. polystachya* resulted in a significantly greater reduction in Hamilton Anxiety Rating Scale (HAM-A) scores compared to placebo at the end of the study (Carmona et al., 2019).

## 10 Applications in nanotechnology and green chemistry of *Buddleja globosa*

### 10.1 Nanotechnology

Green synthesis was conducted using the aqueous extract of *B. globosa*, owing to its high reducing capacity, thereby enabling rapid and efficient biosynthesis of silver nanoparticles (AgNPs). The results indicated that mild conditions, such as room temperature and low concentrations of the plant extract and silver nitrate, were sufficient for AgNP formation (Carmona et al., 2017).

Recently, the contribution of polyphenolic compounds from the aqueous extract of *B. globosa* to the formation of zinc oxide nanoparticles (ZnO NPs) has been highlighted. This contribution is demonstrated by the decrease in TPC during the synthesis reaction, indicating their consumption as reducing and stabilizing agents. Furthermore, the yield of ZnO NPs correlated strongly with TPC values, suggesting that phenolic compounds are primarily responsible for nanoparticle formation and efficiency (Vera et al., 2023).

Polymeric scaffolds were developed from a commercial *B. globosa* extract (BG126®), enriched with chitosan, hyaluronic acid, and gelatin to improve compatibility and antimicrobial activity. Subsequent studies further advanced these biomaterial-based systems by optimizing *B. globosa* extract loading and scaffold composition, showing that biological performance depends on the interaction between extract concentration and physicochemical properties rather than on extract dose alone (Bahamondez-Canas et al., 2026; Ceriani F et al., 2026). These findings support using *B. globosa* extracts as bioactive components in rationally designed scaffold platforms for advanced wound-care applications (Ceriani et al., 2025).

A recent study evaluated compounds present in *B. globosa* leaves as potential natural dyes for dye-sensitized solar cells (DSSCs). The behavior of verbascoside, luteolin 7-*O*-glucoside, apigenin 7-*O*-glucoside, luteolin, and gallic acid during photoexcitation and electron injection into TiO_2_ was analyzed using theoretical calculations (TD-DFT and periodic DFT). Although all molecules showed favorable adsorption to the semiconductor, and some compounds, especially gallic acid and verbascoside, exhibited good electron transfer capacity, the overall conversion efficiency (η) was low, along with a low short-circuit current density (Jsc) and a low form factor (FF). This phenomenon is mainly attributed to the weak photoexcitation of these molecules, which is associated with their wide HOMO–LUMO energy gap, thereby limiting efficient absorption of visible light (Espinoza-Curilén et al., 2024).

### 10.2 Use of green chemistry

Available results indicate that natural deep eutectic solvents (NADES) are a promising green-chemistry alternative to conventional organic solvents for extracting phenolic compounds. In *B. globosa*, the proline-citric acid (1:1) combination has been shown to yield high verbascoside extraction among the evaluated NADES. Meanwhile, luteolin-7-*O*-glucoside extraction is favored by using NADES formulated with choline chloride-propanediol (1:2), which provides higher extraction yields (Torres-Vega et al., 2021).

## 11 Regulatory status in Chile of the medicinal use of *Buddleja globosa*

The leaves of *B. globosa* were officially included in the first Chilean Pharmacopeia (1886), given that various medicinal properties had been attributed to them since earlier times. In Chile, plant species with recognized therapeutic properties are regulated by the national system for the control of pharmaceutical products for human use.

According to Article 8 of Supreme Decree No. 3 of 2010 (Ministerio de Salud de Chile, 2011), the Public Health Institute (ISP) is the body responsible for establishing, through a reasoned resolution, the control regime applicable to products that claim or possess specific properties. In this context, the traditional therapeutic uses of *B. globosa* leaves for treating skin conditions, inflammation, and other pathologies have been officially recognized, thereby allowing their use and commercialization. In this regard, the sanitary registrations for this species are outdated and are mainly concentrated in products manufactured by Knop Laboratorios S.A., including those listed in Table 8.

**TABLE 8:** Current health registrations from the ISP (Chile) for phytotherapeutic products based on *Buddleja globosa* Hope [Scrophulariaceae].

Product	Registration number	Holder	Type	Date
Caléndula-Matico	N-409/25	Knop Laboratorios S.A.	Fluid extract	01-08-1988
Matico Compuesto T.M.	H-815/25	Knop Laboratorios S.A.	Fluid extract	23-08-1988
Matico Extracto Fluido	N-181/21	Knop Laboratorios S.A.	Oral drops	10-05-1990
Matikomp	K-74/25	Knop Laboratorios S.A.	Topical ointment	04-05-1988
Dermimam	N-211/21	Laboratorio Pasteur S.A.	Cream	02-05-1991
G.U.G. Solución	H-717/25	Laboratorio Ximena Polanco González	Oral drops	08-02-1989

Institute of Public Health of Chile. ISPCH sanitary records. Santiago: ISP; [cited 2026 June 26]. Available from: https://registrosanitario.ispch.gob.cl/.

However, infusion bags containing ground *B. globosa* are marketed in Chile under the Food Sanitary Code (DS 977/97).

Packaged and labeled dried leaves can be marketed as Traditional Herbal Medicines (THM), as they were included in Exempt Resolution No. 522 of 2007 (Ministerio de Salud de Chile, 2007). The recognized properties based on traditional use correspond to the aqueous extract, which is indicated for internal use as a healing agent for digestive ulcers, indigestion, stomach pain, and liver dysfunction. Similarly, it is indicated for external use as a vulnerary, with healing and antiseptic actions on wounds. The use of the infusion in compresses to treat bruises and hematomas is also recognized, as are its analgesic, antifungal, healing, and anti-inflammatory effects.

This medicinal basis, although not systematically updated, has been progressively validated over time and has become part of the list approved by Technical Standard No. 133 and Exempt Decree No. 25 of 2012 of the MINSAL (Ministerio de Salud de Chile, 2012), enabling greater development of *B. globosa*.

For medicinal plants to become a real, safe, and evidence-based alternative, it is essential to strengthen public-sector research and to promote a greater role for the Chilean state. This would allow not only the evaluation and validation of their therapeutic properties but also the implementation of robust quality-control methods and the development of educational strategies for consumers. In this way, the safe and effective use of medicinal plants within the public health framework can be ensured (Fuentes-Barros et al., 2025a).

In this sense, the available scientific information on medicinal plants, especially those native to Chile, should be incorporated and made available online free of charge in binding regulatory instruments (Llivisaca-Contreras et al., 2025) as an update of the Chilean pharmacopeia, whose objective is to establish standards of quality, safety, and efficacy that plant drugs and their derivatives must meet for their therapeutic use.

## 12 Propagation and silvicultural management of *Buddleja globosa*

An efficient micropropagation protocol for *B. globosa* was developed through direct organogenesis without callus formation, achieving up to 14.4 shoots per node with benzyladenine, high leaf regeneration with 2,4-dichlorophenoxyacetic acid, and 87% survival after acclimatization (Aboshama, 2011).

There is limited systematic information on methods for obtaining biomass from *B. globosa*. However, it is generally recognized that the use of *B. globosa* populations is strongly influenced by the geographical and climatic characteristics of their habitat. In this context, a rotational management system has been documented to ensure a continuous supply of plant material throughout the year, based on apical pruning by manual topping. The cut length varies seasonally, decreasing to approximately 15 cm in winter and increasing to 40 cm in summer, allowing greater use of the plant’s vegetative vigor during periods of higher temperatures (Wilckens et al., 2013).

Harvesting logistics are adapted to altitude to overcome climatic limitations. During winter, the harvest is concentrated in low-altitude areas, as snow or low temperatures hinder access to mountain areas, which are reserved for the summer season (Wilckens et al., 2013).

Regeneration capacity is a key factor that determines significant differences in productivity between areas. Plants growing at lower altitudes exhibit higher growth rates, allowing for a second harvest in autumn. In contrast, regeneration in mountain environments is considerably slower, resulting in shorter new shoots and limiting the frequency and intensity of harvesting (Wilckens et al., 2013).

The phenological behavior of the species varies markedly across environments. In the valley, the vegetative cycle is longer, lasting approximately 8 months, with a relatively short winter dormancy period of about 4 months. In the mountains, growth is late and brief, lasting about 6 months, followed by a winter dormancy period that can extend up to 6 months. Likewise, flowering occurs early in valley populations, between September and November, whereas in mountain areas it is delayed until December or January due to accumulated cold. Morphologically, valley plants develop more numerous sylleptic shoots and a more highly branched architecture, whereas mountain plants exhibit a simpler, less branched architecture (Wilckens et al., 2013).

In terms of productivity, a significant gap exists between the two environments. While a valley plant can regenerate biomass at approximately 10.7% after pruning, in the Andes Mountains, this rate barely reaches 1.97%. Accordingly, the average yield per plant differs markedly, with values of approximately 450 g of dry weight in valley populations compared to only 25 g in Andean populations, a difference attributed mainly to the less developed branches in the latter. Finally, basal pruning is considered a highly aggressive practice for the plant, especially in Andean areas, where recovery rates are minimal, close to 0.95% (Wilckens et al., 2013).

Therefore, many aspects remain to be explored further, for example, the use of waste generated by *B. globosa* leaves, since in other species they have been considered an important source of polyphenols, particularly verbascoside (Cardullo et al., 2025).

## 13 Cultivation of *Buddleja globosa*

Phenylethanoid glycosides, such as echinacoside and verbascoside, constitute a family of natural compounds of recognized nutraceutical and pharmaceutical importance. Despite the high demand for these compounds across various industries, traditional extraction methods face limitations due to biological variability and the fact that plants sometimes produce insufficient quantities for industrial scaling (Bai et al., 2025).

For this reason, it is imperative to implement rational management practices aimed at biomass production for chemical and medicinal purposes. Given the relevance of their properties and the growing demand, the need to explore alternative production methods is highlighted; for example, metabolically engineered yeasts have been developed to synthesize these compounds from simple precursors, such as glucose (Bai et al., 2025).

The biomass yield of *B. globosa* is closely related to its origin and management practices. Cultivated accessions exhibit higher leaf biomass production than wild populations, a pattern attributed to prior informal selection. From an optimal management perspective, one or two harvests per year are recommended to maximize total dry weight, while three harvests per season improve the leaf-to-stem ratio (Vogel et al., 2004).

From a chemical perspective, no significant differences in flavonoid and tannin concentrations were detected between wild and cultivated specimens (Vogel et al., 2004). However, it is crucial to consider the temporal evolution of individual phytochemistry, since over the years, variability in polyphenol content among different cultivated origins begins to emerge (Vogel et al., 2010), with the harvest time being a key aspect for taking advantage of the higher metabolite and biomass content (Vogel et al., 2002). Additionally, developmental factors, such as plant age and tissue maturity, affect composition; flavonoid concentration increases during the second growing season and is higher in mature leaves than in young ones (Vogel et al., 2004). This has been observed in other verbascoside-containing species, with significant variation (Deb and Sarkar, 2022; Mabusela et al., 2025).

Another study observed that flavonoid content ranged from 9.7% in leaves from plants in the Andes Mountains to 13.9% in leaves from plants in the Chilean Coastal Range (Fischer et al., 2011). This indicates a significant gap in our understanding of the genetic influences and interactions with environmental factors on secondary metabolite variability (Fuentes-Barros et al., 2018; Fuentes-Barros et al., 2023).

Intrinsic and extrinsic factors influence this phytochemical variability. The content of secondary metabolites in native populations has been shown to vary with geographic origin, environmental factors, and biological variables, including plant sex, age, and tissue type (young or mature) (Fuentes-Barros et al., 2018; 2023; Giordano et al., 2019). In the specific case of *B. globosa*, environmental factors could play a critical role in differentiating chemical profiles at the population level.

Furthermore, management practices directly influence the metabolic profile: subjecting plants to multiple harvests in the same season significantly increases flavonoid concentrations toward the last harvest (Vogel et al., 2004). Water stress also plays a relevant role, with higher polyphenol content observed in plants with lower irrigation (Vogel et al., 2010).

Regarding nutritional requirements, the nitrogen needs of *B. globosa* are comparable to those reported for other medicinal species (Vogel et al., 2004). However, monitoring should consider a sufficient time horizon and greater specificity in the metabolites analyzed. This is because the authors later confirmed that geographic origin, irrigation level, or the initial domestication process did not significantly affect the flavonoid and polyphenol content. Although water stress reduced foliar biomass size and yield, medicinal quality (based on active principles) did not differ significantly (Vogel et al., 2011).

The roots of *B. globosa* develop easily (Doll et al., 2003; Seemann et al., 2013). However, applying indolebutyric acid (IBA) at the base of the cutting promotes and accelerates this process, with the best results obtained at concentrations of 1000 and 2000 ppm (83%). To optimize propagation, it is recommended to use apical cuttings and a substrate that retains adequate moisture, such as a perlite-vermiculite mixture (Doll et al., 2003).

## 14 Perspectives and challenges in the standardization of *Buddleja globosa*

Currently, precise records of verbascoside yield in the different organs of *B. globosa* are not available. However, general studies suggest that this metabolite is usually present in concentrations ranging from 0.2% to 3% (Deng et al., 2024), which depends critically on factors such as origin, cultivation practices, and harvesting conditions.

To advance the biotechnological valorization of the species, future research must broaden its scope. This requires significant investment in human resources and technology to investigate growth and cultivation and to expand the sample size to at least 25 individuals per treatment. This is based on the fact that the content of some metabolites is highly variable, and systematically monitoring all plant components (leaf, stem, and bark) for the main secondary metabolites is an indispensable scientific pillar to guarantee the reproducibility and quality of extracts intended for therapeutic purposes (Castro-Saavedra et al., 2016; Fuentes-Barros et al., 2018; 2023; 2025b; Cassels et al., 2019).

## 15 Influence of drying on active ingredients

To preserve the integrity of secondary metabolites, it is recommended to dry *B. globosa* leaves at low temperatures, in a protected location away from direct sunlight. Evidence indicates that using high temperatures (60 °C and 80 °C) yields extracts with significantly lower polyphenol concentrations (Vogel et al., 2010). This thermal degradation is critical even at moderate temperatures; for example, drying at 45 °C reduces the flavonoid content by 66.2% compared to drying at 5 °C (Hevia et al., 2006).

Furthermore, although dehydration alters the chromatic properties of the plant material, no direct association was found between final leaf color (determined using Munsell color charts) and the plant material’s flavonoid concentration (Hevia et al., 2006).

Taken together, these findings highlight that drying conditions can significantly affect the preservation of bioactive compounds in *B. globosa* and underscore the need to further investigate cultivation, processing, and chemotaxonomic variability in relation to verbascoside and other pharmacologically relevant metabolites.

## 16 Methodological considerations and reporting standards

The 50 primary studies included in this systematic review were published over nearly 5 decades (1979–2026), during which reporting standards for phytochemical and pharmacological research on medicinal plant extracts have evolved considerably. Many earlier studies, and even some recent publications, did not report complete methodological details on plant material authentication, voucher specimen deposition, extraction conditions (e.g., temperature, particle size, plant-to-solvent ratio), or comprehensive chemical characterization of extracts.

This heterogeneity in reporting quality is a recognized limitation in ethnopharmacology and natural products research, as it hinders reproducibility and comparability across studies. In this context, we support the recent consensus-based reporting guidelines for Phytochemical Characterisation of Medicinal Plant extracts (ConPhyMP), which provide a structured framework for reporting essential information on plant material, extraction procedures, and analytical methods (Heinrich et al., 2022).

Future experimental studies on *B. globosa* and other medicinal plant species should adhere to these ConPhyMP guidelines to ensure transparency, reproducibility, and scientific rigor. This will be particularly important as the field moves toward developing standardized phytotherapeutic products and translating traditional knowledge into evidence-based healthcare applications.

## 17 Quality of the evidence and translational value of the included studies

The methodological quality and risk-of-bias assessments, summarized in Supplementary Table S5, revealed a heterogeneous body of evidence. Of the 50 included studies, only two provided Level I evidence (clinical trials), while the majority were classified as Level IV or V (mechanistic *in vitro* and exploratory studies). Although several *in vitro* studies (Level V) suggest antioxidant and antimicrobial activity, their translational value is limited by non-physiological concentrations and the lack of pharmacokinetic data. In contrast, the clinical trial reported by Rubio Lara (2011) provides robust Level I evidence, despite its moderate sample size.

The main limitations identified across all studies include the lack of standardized extracts, the absence of chronic toxicity studies, and the predominance of *in vitro* models that do not fully capture the complexity of the human organism. These limitations, detailed in Supplementary Table S5, have important implications for interpreting the overall body of evidence and highlight the need for future research.

Based on the critical limitations identified, future research should prioritize: (i) dose-finding studies to establish the optimal concentration of standardized *B. globosa* extracts; (ii) long-term safety evaluations, including chronic toxicity and genotoxicity studies; (iii) mechanism-of-action studies in humans, particularly for the clinical effects observed in wound healing and radioepithelitis; and (iv) standardization of extracts to ensure reproducibility and comparability across studies.

## 18 Final observations and perspectives

*Buddleja globosa* is a shrub whose leaves are used medicinally. Its medicinal use has a documented history of over 140 years, although efficacy and safety require further toxicological and clinical evaluation. It is currently one of the three most widely used native medicinal species in Chile. In particular, given the feasibility of its cultivation, it is imperative to further its scientific study, specifically in the development of propagation techniques that allow us to address population chemotaxonomic variability in relation to verbascoside content and other variables of pharmacological interest, as has been observed in the study of secondary metabolites in other species (Estrada-Zúñiga et al., 2016; Soares et al., 2016; Fuentes-Barros et al., 2023).

Considering available technologies, all activities aimed at producing NTFPs from *B. globosa* should strategically focus on orchard management models or silvicultural systems that allow for the sustainable and standardized production of its key metabolites, such as verbascoside, echinacoside, buddledin A, α-amyrin, β-amyrin, luteolin, apigenin, luteolin-7-*O*-glucoside, apigenin-7-*O*-glucoside, and rutin. This development must integrate the traditional knowledge involved, ensuring recognition and compensation for these communities.

As a research strategy, it is necessary to more clearly establish the chemical signature of all components of the species (Fuentes-Barros et al., 2025b). This will enable the combined application of tools such as integrative dereplication, molecular networks, and systems pharmacology, constituting a rational and efficient approach to studying natural products (Castro-Saavedra et al., 2026). Furthermore, the incorporation of genomic and transcriptomic tools would enable the identification of verbascoside biosynthetic pathways, particularly those associated with the phenylpropanoid pathway, thereby facilitating understanding of the chemical variability observed among populations, seasons, and management systems.

From a public policy perspective, given the high consumption of this species by the population, the formal information on the biomass of *B. globosa* must incorporate quantitative analyses of its metabolites in the species monograph. Verbascoside emerges as the main chemomarker; validated analytical protocols using HPLC-DAD could be adapted specifically to *B. globosa* and incorporated into the national pharmacopeia in open-access digital formats, facilitating compliance with current regulations.

Finally, given the high concentration of active metabolites in the leaves, their recognized biological activity, and their adequate solubility in infusions and topical formulations, systematic quality studies are essential. The lack of evidence that production is conducted in accordance with Good Manufacturing Practices (GMP) currently limits oversight, affecting the safety and efficacy of products on the market (Fuentes-Barros et al., 2025a). It is imperative to adopt the One Health approach to provide a scientific basis that answers what we want to achieve and how to achieve it, ensuring the standardization of biomass and the development of public policies aimed at the sustainable use of this valuable native medicinal species (Sanchéz et al., 2026).

Beyond standardization and quality control, the clinical translation of *B. globosa* remains in its early stages and requires deeper mechanistic characterization of its bioactive extracts and marker compounds. Although traditional use and preclinical evidence support its relevance to wound healing, inflammation, antimicrobial protection, gastrointestinal disorders, and liver-related conditions, most available studies remain based on crude extracts, isolated compounds, or early experimental models. Future studies should therefore evaluate chemically standardized *B. globosa* preparations in well-controlled dose-response and time-course assays using disease-relevant cellular systems, including keratinocytes, fibroblasts, macrophages, dendritic cells, and T lymphocytes. These studies should clarify how the extracts and key metabolites, such as verbascoside, echinacoside, linarin, luteolin derivatives, buddledin A, and amyrins, modulate inflammatory, oxidative, antimicrobial, and tissue-repair pathways, including cytokine production, inflammasome activation, NF-κB/MAPK/Nrf2 signaling, macrophage polarization, T-cell activation and differentiation, extracellular matrix remodeling, cell migration, and epithelial differentiation. Particular the biological effects depend on individual compounds or on synergistic interactions within the whole extract. These cellular studies should be complemented by *in vivo* experiments using standardized preparations in disease-relevant models, including cutaneous wound healing, infected wounds and biofilm-associated lesions, gastric ulcer models, liver injury models, and oral inflammatory diseases such as periodontitis and apical periodontitis, where outcomes should include tissue repair, microbial burden, inflammatory infiltrates, immune-cell phenotyping, bone or soft-tissue destruction, histological regeneration, systemic toxicity, pharmacokinetics, and local biodistribution. Only after these mechanistic, pharmacological, and toxicological gaps are addressed should *B. globosa*-based products advance to well-designed clinical studies. These should begin with safety, tolerability, and dose-finding trials using chemically standardized preparations, followed by controlled clinical trials in specific indications, such as wound healing, oral inflammatory diseases, gastrointestinal protection, and other conditions in which traditional use and preclinical evidence converge.

## Data Availability

The original contributions presented in the study are included in the article/Supplementary Material, further inquiries can be directed to the corresponding author.

## Footnotes

1 The Mapuche are an Indigenous people of south-central Chile and neighboring Argentina, with a long-standing cultural tradition and a deep historical connection to medicinal plants and local ecological knowledge.

2 Quechua is an Indigenous language and cultural tradition of the Andean region; “palguín” is one of the local names used for Buddleja globosa in Quechua-speaking contexts. In Chile, the plant is also widely known as “matico,” a common vernacular name used in traditional medicine.
