# Supplementary material for: Phytochemistry, pharmacology, safety, and applications of Buddleja globosa Hope [Scrophulariaceae]: a systematic review from bioactive metabolites and mechanisms to translational perspectives
Source: Front Pharmacol. 2026 Oct 14;17:1940132. doi: 10.3389/fphar.2026.1940132 (PMC13713865; doi:10.3389/fphar.2026.1940132)
Supplement: Supplementary file 1 [file Table1.docx]

**SUPPLEMENTARY MATERIAL**

**Phytochemistry, pharmacology, safety, and applications of *Buddleja globosa* Hope [Scrophulariaceae]: A systematic review from bioactive metabolites and mechanisms to translational perspectives**

Gonzalo Fuentes-Barros^1,2^, Sebastián Castro-Saavedra^1,3^, Nicolás Montalva^4^, Marco Mellado^5,6^, Rolando Vernal^7,8^, Marcela Hernández^7,9^, Antonia Díaz-Valdés^4,10,11^, Camila Zoppi^1^, Javier Echeverría^3,*^

^1^ SAPHYCHEM, Santiago, Chile.

^2^ Programa de Doctorado en Políticas Públicas, Universidad Mayor, Santiago, Chile.

^3^ Departamento de Ciencias del Ambiente, Facultad de Química y Biología, Universidad de Santiago de Chile, Santiago, Chile.

^4^ Centro de Investigación en Sociedad y Salud, Facultad de Ciencias Sociales y Artes, Universidad Mayor, Santiago, Chile.

^5^ Dirección de Investigación, Universidad Bernardo O’Higgins, Santiago, Chile.

^6^ Laboratorio de Espectroscopía y Química Aplicada, Grupo de Investigación en Ciencias Biomédicas Aplicadas, Universidad Central de Chile, Santiago, Chile.

^7^ Laboratorio de Biología Periodontal, Facultad de Odontología, Universidad de Chile, Santiago, Chile.

^8^ Departamento de Odontología Conservadora, Facultad de Odontología, Universidad de Chile, Santiago, Chile.

^9^ Departamento de Patología y Medicina Oral, Facultad de Odontología, Universidad de Chile, Santiago, Chile

^10^ Facultad de Ciencias Sociales y Artes, Psicología, Universidad Mayor, Santiago, Chile.

^11^ Centro de Gerociencia para la Salud Cerebral y el Metabolismo, Santiago, Chile.

*Correspondence: [javier.echeverriam@usach.cl](mailto:javier.echeverriam@usach.cl)

**Figure S1**. Egg-boiling scheme of some representative compounds identified in *B. globosa*. luteolin (**1**), luteolin 7-*O*-glucoside (**3**), apigenin 7-*O*-glucoside (**5**), apigenin (**8**), acteoside / verbascoside (**39**), echinacoside (**40**), α-amyrin (**59**), β-amyrin (**60**), buddledin A (**100**).

**Table S1.** Methodological and thematic comparison between the systematic review by Becerra et al. (2026) and the present systematic and bibliometric review of *B. globosa*.

| **Dimension** | **Becerra et al. (2026)** | **Present review** |
| --- | --- | --- |
| **Article type** | Systematic review | Systematic review |
| **Primary scope** | Phytochemical composition and antioxidant/anti-inflammatory activities of *Buddleja globosa.* | Integrated assessment of the phytochemistry, pharmacology, safety, clinical evidence, applications, cultivation, regulation, and standardization of *Buddleja globosa.* |
| **Studies included** | 14 | 50 |
| **Bioactive compounds reported** | 27 | 119 |
| **Main biological endpoints** | Primarily antioxidant and anti-inflammatory activities. | Antioxidant, anti-inflammatory, analgesic, wound-healing, antimicrobial, antifungal, antiplatelet, antihepatotoxic, and other preclinical activities, as well as evidence from clinical studies. |
| **Clinical evidence** | Not a primary focus of the review. | Clinical evidence incorporated to complement the preclinical and pharmacological evidence. |
| **Evidence base** | Focused on the phytochemical and pharmacological literature addressing antioxidant and anti-inflammatory activities. | Broader evidence base encompassing phytochemical, preclinical, clinical, ethnopharmacological, regulatory, silvicultural, technological, and standardization-related evidence. |
| **Translational perspective** | Primarily focused on the pharmacological relevance of antioxidant and anti-inflammatory activities. | Expanded translational perspective encompassing clinical evidence, standardization, regulation, cultivation, processing, formulation, nanotechnology, green chemistry, and product-development implications |
| **Search sources** | PubMed, EBSCOhost, Springer Nature, and Google Scholar as a complementary source | Web of Science Core Collection, Scopus, PubMed, SciFinder, and the University of Chile Repository; WIPO searched separately for patent information |
| **Verbascoside** | Identified as an important phytochemical constituent associated with the biological activity of *B. globosa* within the focused phytochemical and pharmacological scope. | Examined as a major bioactive marker compound, with evidence discussed in relation to pharmacological activity, standardization, formulation, and translational relevance. |
| **Clinical and emerging applications** | Not a primary focus. | Includes clinical evidence and emerging applications of *B. globosa* in nanotechnology and green chemistry. |
| **Regulatory and production aspects** | Not a primary focus. | Includes the regulatory status of the medicinal use of *B. globosa* in Chile, propagation, cultivation, and silvicultural management. |
| **Standardization and processing** | Not a primary focus. | Addresses perspectives and challenges in standardizing *B. globosa*, including how drying conditions influence active constituents. |
| **Future perspectives** | Focused on the pharmacological relevance of the reviewed evidence. | Provides integrated final observations and perspectives addressing research gaps, standardization, translational challenges, and future research priorities. |
| **Coverage of landmark authors and studies** | Focused on the studies included within its narrower phytochemical and pharmacological scope. | Integrates major international and national contributions to *B. globosa*, including landmark work by Houghton and Mensah and foundational Chilean research led by María Eugenia Letelier and other national researchers. |
| **Overall contribution** | Provides a focused synthesis of the phytochemical composition and antioxidant/anti-inflammatory evidence of *B. globosa.* | Extends and complements the previous focused review by integrating a broader systematic evidence base with bibliometric analysis and additional clinical, translational, regulatory, technological, cultivation, and standardization perspectives. |
| **Language coverage** | The review included studies published in English. | The present review included studies published in English and Spanish, allowing the incorporation of relevant Latin American and Chilean literature. |

**Table S2.** Database coverage of the publications included in the systematic review.

| **N°** | **Title** | **WoS** | **Scopus** | **Pubmed** | **Scifinder** | **University of Chile repository** | **Grey literature** |
| --- | --- | --- | --- | --- | --- | --- | --- |
| 1 | An In Vitro Study of the Antioxidant and Antihemolytic Properties of *Buddleja globosa* (Matico) | ✓ | ✓ | ✓ | ✓ |  |  |
| 2 | Analgesic, anti-inflammatory and antioxidant properties of *Buddleja globosa*, Buddlejaceae | ✓ | ✓ | ✓ | ✓ |  |  |
| 3 | Anti-Hepatotoxic Activity of Extracts and Constituents of Buddleja Species |  |  | ✓ | ✓ |  |  |
| 4 | Antimutagenic activity of *Buddleja globosa* extracts in the drosophila wing-spot test | ✓ | ✓ |  | ✓ |  |  |
| 5 | Antinociceptive activity of *Buddleja globosa* (matico) in several models of pain | ✓ | ✓ | ✓ | ✓ |  |  |
| 6 | Antioxidant activity and cytotoxic effect of Chilean *Buddleja globosa* (matico) and *Ribes magellanicum* (zarzaparrilla) flower extracts | ✓ | ✓ |  |  |  |  |
| 7 | Antioxidant Activity as an Indicator of the Efficiency of Plant Extract-Mediated Synthesis of Zinc Oxide Nanoparticles | ✓ |  | ✓ | ✓ |  |  |
| 8 | Antioxidant activity in teas, tinctures and essential oils of native species from Patagonia Argentina |  | ✓ |  | ✓ |  |  |
| 9 | Antioxidant capacity of herbal infusions and tea extracts: A comparison of ORAC-fluorescein and ORAC-pyrogallol red methodologies |  |  |  | ✓ |  |  |
| 10 | Antioxidant screening of medicinal herbal teas |  | ✓ |  | ✓ |  |  |
| 11 | BG126® phytodrug improves urinary tract infection treatment with nitrofurantoin in adult women in a double-blind randomized clinical trial | ✓ | ✓ |  |  |  |  |
| 12 | *Buddleja globosa* (matico) prevents collagen-induced platelet activation by decreasing phospholipase C-gamma 2 and protein kinase C phosphorylation signaling |  | ✓ | ✓ | ✓ |  |  |
| 13 | *Buddleja globosa* Extract-Enriched Gelatin-Chitosan-Hyaluronic Acid Scaffold Biocomposite Exhibits Enhanced Angiogenesis and Dermal Tissue Organization in a Chronic Wound Model | ✓ |  | ✓ | ✓ |  |  |
| 14 | *Buddleja globosa* Leaf Methanolic Extract Acts Against *Trypanosoma cruzi* Parasites by Inducing Mitochondrial Inner Membrane Hyperpolarization | ✓ | ✓ | ✓ | ✓ |  |  |
| 15 | Buddlejone, a diterpene from *Buddleja albiflora* | ✓ |  |  | ✓ |  |  |
| 16 | Characterization and propagation of some medicinal plants in the central-south region of Chile | ✓ |  |  |  |  |  |
| 17 | Chemical Composition, Antimicrobial Activity, and Mode of Action of Essential Oils against *Paenibacillus* larvae, Etiological Agent of American Foulbrood on *Apis mellifera* | ✓ |  | ✓ | ✓ |  |  |
| 18 | Complementary and alternative medicine in patients with inflammatory bowel disease: A survey performed in a tertiary center in Chile | ✓ |  |  |  |  |  |
| 19 | DPPH and oxygen free radicals as pro-oxidant of biomolecules | ✓ | ✓ | ✓ | ✓ |  |  |
| 20 | Effect of different provenances and production conditions on antioxidant properties in *Buddleja globosa* leaves | ✓ |  |  | ✓ |  |  |
| 21 | Effects of *Buddleja globosa* leaf and its constituents relevant to wound healing | ✓ | ✓ | ✓ | ✓ |  |  |
| 22 | Efficient in vitro shoot regeneration responses of *Buddelia globosa* |  |  |  | ✓ |  |  |
| 23 | Electrospun fibers loaded with extracts of *Gunnera tinctoria* and *Buddleja globosa* with potential application in the treatment of skin lesions | ✓ | ✓ |  | ✓ |  |  |
| 24 | Estrous cycle disruptor effect of an ethanolic extract from *Buddleja globosa* leaves and its main component (verbascoside) | ✓ | ✓ |  |  |  |  |
| 25 | Evaluation of the antioxidant capacity of herbal teas by a procedure based on the bleaching of ABTS radical cations |  |  |  | ✓ |  |  |
| 26 | Formulation of water-soluble *Buddleja globosa* Hope extracts and characterization of their antimicrobial properties against Pseudomonas aeruginosa | ✓ | ✓ | ✓ | ✓ |  |  |
| 27 | Green synthesis of silver nanoparticles by using leaf extracts from the endemic *Buddleja globosa* hope | ✓ | ✓ |  | ✓ |  |  |
| 28 | Herbal extracts differentially inhibit oxidative effects caused by the biotransformation of nifurtimox, nitrofurantoin and acetaminophen on rat liver microsomes | ✓ | ✓ |  | ✓ |  |  |
| 29 | In vitro antiplasmodial activity of extracts of Argentinian plants | ✓ | ✓ | ✓ | ✓ |  |  |
| 30 | In vitro tests and ethnopharmacological investigations: Wound healing as an example | ✓ |  | ✓ | ✓ |  |  |
| 31 | In-vitro investigation of the wound-healing properties of *Buddleja globosa* |  | ✓ |  | ✓ |  |  |
| 32 | Influence of dry leaves of common tree and shrub species from southern Chile on the growth of lettuce infected with *Meloidogyne hapla* | ✓ |  |  |  |  |  |
| 33 | Influence of the Target Molecule on the Oxygen Radical Absorbance Capacity Index: A Comparison Between Alizarin Red- and Fluorescein-Based Methodologies |  |  |  | ✓ |  |  |
| 34 | Isolation of verbascoside, an antimicrobial constituent of *Buddleja globosa* leaves | ✓ |  | ✓ | ✓ |  |  |
| 35 | Known and novel terpenes from *Buddleja globosa* displaying selective antifungal activity against dermatophytes | ✓ | ✓ | ✓ | ✓ |  |  |
| 36 | Morphological characters, yields and active principles in wild and cultivated accessions of the Chilean medicinal plant *Buddleja globosa* Hope | ✓ |  |  |  |  |  |
| 37 | Nematicidal activity of leaves of common shrub and tree species from Southern Chile against *Meloidogyne hapla* | ✓ | ✓ |  |  |  |  |
| 38 | Novel and known constituents from *Buddleja* species and their activity against leukocyte eicosanoid generation | ✓ |  | ✓ | ✓ |  |  |
| 39 | Novel glycoside from *Buddleja globosa* with bactericidal activity | ✓ | ✓ |  | ✓ |  |  |
| 40 | Nutraceutical Properties of Herbal Infusions from Six Native Plants of Argentine Patagonia | ✓ |  | ✓ | ✓ |  |  |
| 41 | Optimization of *Buddleja globosa*-Loaded Polymeric Scaffolds for the Treatment of Biofilm-Infected Wounds | ✓ | ✓ | ✓ | ✓ |  |  |
| 42 | Phenolic fatty acid esters from *Buddleja globosa* stem bark | ✓ |  |  | ✓ |  |  |
| 43 | Phytochemical study of *Buddleja globosa* Lam. (Buddlejaceae) | ✓ |  |  | ✓ |  |  |
| 44 | Polyphenolic Compounds Extracted and Purified from *Buddleja Globosa* Hope (Buddlejaceae) Leaves Using Natural Deep Eutectic Solvents and Centrifugal Partition Chromatography | ✓ | ✓ | ✓ | ✓ |  |  |
| 45 | Southern Chilean Native Plants as Novel Sources of Antioxidant and Antibacterial Extracts | ✓ |  | ✓ | ✓ |  |  |
| 46 | Sustainable management and use of a medicinal emblematic plant in Chile: *Buddleja globosa* Hope | ✓ | ✓ |  |  |  |  |
| 47 | Tailoring Polymeric Scaffolds with *Buddleja globosa* Extract for Dual Antimicrobial and Biocompatible Wound Healing Applications | ✓ | ✓ | ✓ | ✓ |  |  |
| 48 | The aromatic plant essential oils and their hormetic effect on *Rhyzopertha dominica* (Coleoptera: Bostrichidae) |  |  |  | ✓ |  |  |
| 49 | Clinical evaluation of a matico extract in the treatment of palmar-plantar erythrodysesthesia secondary to chemotherapy and in the prophylaxis of radioepithelitis in patients undergoing radiotherapy (Doctoral thesis) |  |  |  |  | ✓ |  |
| 50 | Castro-Saavedra et al. (2016). Potencial Medicinal de 19 Especies de la Flora Chilena de la Zona Central, in V Congreso Iberoamericano de Productos Naturales, Santiago: Sociedad Colombiana de Ciencias Químicas. |  |  |  |  |  | ✓ |

Data extracted from each study include title, author(s), journal, year of publication, country of publication, study characteristics (study design, study duration), characteristics of participants (gender, age, sample size), plant type and extractions, intervention (type of treatment) and control used, number of dropouts, methods, and results from the studies.

**Table S3**. Pharmacokinetic properties of secondary metabolites identified from *Buddleja globosa*.

| **n°** | **MW^1^** | **HBA^2^** | **HBD^3^** | **iLOGP^4^** | **LogS^5^** | **GI^6^** | **BBB^7^** | **P-gp^8^** | **CYP_1A2_^9^** | **CYP_2C19_^10^** | **CYP_2C9_^11^** | **CYP_2D6_^12^** | **CYP_3A4_^13^** | **Log K^14^** | **Lipinski^15^** |
| --- | --- | --- | --- | --- | --- | --- | --- | --- | --- | --- | --- | --- | --- | --- | --- |
| **1** | 286.24 | 6 | 4 | 1.86 | -3.71 | High | No | No | Yes | No | No | Yes | Yes | -6.25 | 0 |
| **2** | 302.24 | 7 | 5 | 1.94 | -3.55 | High | No | No | Yes | No | No | Yes | Yes | -6.6 | 0 |
| **3** | 448.38 | 11 | 7 | 1.76 | -3.65 | Low | No | Yes | No | No | No | No | No | -8 | 2 |
| **4** | 464.38 | 12 | 8 | 2.1 | -3.51 | Low | No | Yes | No | No | No | No | No | -8.35 | 2 |
| **5** | 432.38 | 10 | 6 | 1.98 | -3.78 | Low | No | Yes | No | No | No | No | No | -7.65 | 1 |
| **6** | 610.52 | 16 | 10 | 0.46 | -3.3 | Low | No | Yes | No | No | No | No | No | -10.26 | 3 |
| **7** | 302.24 | 7 | 5 | 1.63 | -3.16 | High | No | No | Yes | No | No | Yes | Yes | -7.05 | 0 |
| **8** | 270.24 | 5 | 3 | 1.89 | -3.94 | High | No | No | Yes | No | No | Yes | Yes | -5.8 | 0 |
| **9** | 448.38 | 11 | 7 | 1.6 | -3.33 | Low | No | No | No | No | No | No | No | -8.42 | 2 |
| **10** | 254.24 | 4 | 2 | 2.27 | -4.19 | High | Yes | No | Yes | No | No | Yes | Yes | -5.35 | 0 |
| **11** | 464.38 | 12 | 8 | 0.94 | -3.04 | Low | No | No | No | No | No | No | No | -8.88 | 2 |
| **12** | 594.52 | 15 | 9 | 2.08 | -3.29 | Low | No | Yes | No | No | No | No | No | -10.06 | 3 |
| **13** | 740.66 | 19 | 11 | 2.56 | -3.33 | Low | No | Yes | No | No | No | No | No | -11.73 | 3 |
| **14** | 592.55 | 14 | 7 | 2.75 | -3.65 | Low | No | Yes | No | No | No | No | No | -9.56 | 3 |
| **15** | 300.26 | 6 | 3 | 2.27 | -3.99 | High | No | No | Yes | No | No | Yes | Yes | -6.01 | 0 |
| **16** | 284.26 | 5 | 2 | 2.48 | -4.14 | High | No | No | Yes | No | Yes | Yes | Yes | -5.66 | 0 |
| **17** | 624.54 | 16 | 9 | 2.94 | -3.73 | Low | No | Yes | No | No | No | No | No | -9.87 | 3 |
| **18** | 592.55 | 14 | 7 | 2.47 | -3.65 | Low | No | Yes | No | No | No | No | No | -9.56 | 3 |
| **19** | 610.52 | 16 | 10 | 0.46 | -3.3 | Low | No | Yes | No | No | No | No | No | -10.26 | 3 |
| **20** | 448.38 | 11 | 7 | 0.99 | -3.05 | Low | No | No | No | No | No | No | No | -8.67 | 2 |
| **21** | 606.57 | 14 | 7 | 3.53 | -3.29 | Low | No | Yes | No | No | No | No | No | -10.13 | 3 |
| **22** | 610.52 | 16 | 12 | 1.8 | -1.93 | Low | No | No | No | No | No | No | No | -11.88 | 3 |
| **23** | 256.25 | 4 | 2 | 2.11 | -3.64 | High | Yes | No | Yes | Yes | No | No | No | -5.82 | 0 |
| **24** | 290.27 | 6 | 5 | 1.33 | -2.22 | High | No | Yes | No | No | No | No | No | -7.82 | 0 |
| **25** | 290.27 | 6 | 5 | 1.47 | -2.22 | High | No | Yes | No | No | No | No | No | -7.82 | 0 |
| **26** | 287.24 | 6 | 5 | -2.62 | -2.6 | High | No | Yes | Yes | No | No | No | No | -7.51 | 0 |
| **27** | 331.3 | 7 | 4 | -1.96 | -3.6 | High | No | Yes | Yes | No | No | No | No | -6.73 | 0 |
| **28** | 122.12 | 2 | 1 | 1.11 | -2.2 | High | Yes | No | No | No | No | No | No | -5.72 | 0 |
| **29** | 138.12 | 3 | 2 | 1.13 | -2.5 | High | Yes | No | No | No | No | No | No | -5.54 | 0 |
| **30** | 198.17 | 5 | 2 | 1.54 | -1.84 | High | No | No | No | No | No | No | No | -6.77 | 0 |
| **31** | 170.12 | 5 | 4 | 0.21 | -1.64 | High | No | No | No | No | No | No | Yes | -6.84 | 0 |
| **32** | 180.16 | 4 | 3 | 0.97 | -1.89 | High | No | No | No | No | No | No | No | -6.58 | 0 |
| **33** | 194.18 | 4 | 2 | 1.62 | -2.11 | High | Yes | No | No | No | No | No | No | -6.41 | 0 |
| **34** | 164.16 | 3 | 2 | 0.95 | -2.02 | High | Yes | No | No | No | No | No | No | -6.26 | 0 |
| **35** | 152.15 | 3 | 1 | 1.57 | -1.82 | High | Yes | No | No | No | No | No | No | -6.37 | 0 |
| **36** | 336.29 | 8 | 5 | 0.82 | -1.92 | Low | No | No | No | No | No | No | No | -8.19 | 0 |
| **37** | 354.31 | 9 | 6 | 0.87 | -1.62 | Low | No | No | No | No | No | No | No | -8.76 | 1 |
| **38** | 354.31 | 9 | 6 | 1.01 | -1.62 | Low | No | No | No | No | No | No | No | -8.76 | 1 |
| **39** | 624.59 | 15 | 9 | 2.15 | -2.87 | Low | No | Yes | No | No | No | No | No | -10.46 | 3 |
| **40** | 786.73 | 20 | 12 | 2.08 | -2.63 | Low | No | No | No | No | No | No | No | -12.59 | 3 |
| **41** | 624.59 | 15 | 9 | 2.71 | -2.87 | Low | No | Yes | No | No | No | No | No | -10.46 | 3 |
| **42** | 624.59 | 15 | 9 | 2.49 | -2.87 | Low | No | Yes | No | No | No | No | No | -10.46 | 3 |
| **43** | 654.61 | 16 | 9 | 0.58 | -2.62 | Low | No | No | No | No | No | No | No | -11.06 | 3 |
| **44** | 652.64 | 15 | 7 | 3.55 | -3.32 | Low | No | Yes | No | No | No | No | No | -10.17 | 3 |
| **45** | 608.59 | 14 | 8 | 3.11 | -3 | Low | No | Yes | No | No | No | No | No | -10.12 | 3 |
| **46** | 666.62 | 16 | 8 | 3.23 | -3.17 | Low | No | Yes | No | No | No | No | No | -10.52 | 3 |
| **47** | 756.7 | 19 | 11 | 1.87 | -2.69 | Low | No | No | No | No | No | No | No | -12.14 | 3 |
| **48** | 756,7 | 19 | 11 | Un | -2,56 | Low | No | No | Un | Un | Un | Un | Un | -12,36 | 3 |
| **49** | 346.33 | 9 | 6 | 0.56 | 0.18 | Low | No | Yes | No | No | No | No | No | -10.56 | 1 |
| **50** | 362.33 | 10 | 6 | 1.45 | 0.21 | Low | No | Yes | No | No | No | No | No | -10.8 | 1 |
| **51** | 506.5 | 11 | 5 | 2.86 | -2.26 | Low | No | No | No | No | No | No | No | -9.67 | 2 |
| **52** | 522.5 | 12 | 5 | 2.38 | -2.23 | Low | No | Yes | No | No | No | No | No | -9.91 | 2 |
| **53** | 482.43 | 12 | 6 | 2.11 | -1.66 | Low | No | No | No | No | No | No | No | -10.19 | 2 |
| **54** | 376.36 | 10 | 5 | 1.6 | -0.16 | Low | No | Yes | No | No | No | No | No | -10.5 | 0 |
| **55** | 300.44 | 2 | 1 | 3.44 | -5.73 | High | Yes | No | No | Yes | Yes | No | No | -3.75 | 0 |
| **56** | 284.44 | 1 | 0 | 3.6 | -5.17 | High | Yes | No | No | Yes | Yes | No | Yes | -3.94 | 1 |
| **57** | 604.9 | 4 | 2 | 4.62 | -9.07 | Low | No | No | No | No | No | No | No | -3.66 | 2 |
| **58** | 426.72 | 1 | 1 | 4.72 | -8.64 | Low | No | No | No | No | No | No | No | -1.9 | 1 |
| **59** | 426.72 | 1 | 1 | 4.8 | -8.16 | Low | No | No | No | No | No | No | No | -2.51 | 1 |
| **60** | 426.72 | 1 | 1 | 4.63 | -8.25 | Low | No | No | No | No | No | No | No | -2.41 | 1 |
| **61** | 414.71 | 1 | 1 | 5.05 | -7.9 | Low | No | No | No | No | No | No | No | -2.2 | 1 |
| **62** | 576.85 | 6 | 4 | 5.17 | -7.7 | Low | No | No | No | No | No | No | No | -4.32 | 1 |
| **63** | 412.69 | 1 | 1 | 5.08 | -7.46 | Low | No | No | No | No | Yes | No | No | -2.74 | 1 |
| **64** | 400.68 | 1 | 1 | 4.97 | -7.54 | Low | No | No | No | No | No | No | No | -2.5 | 1 |
| **65** | 416.72 | 1 | 1 | 5.17 | -7.27 | Low | No | No | No | No | No | No | No | -2.93 | 1 |
| **66** | 396.65 | 1 | 1 | 4.81 | -6.72 | Low | No | No | No | No | Yes | No | No | -3.44 | 1 |
| **67** | 150.22 | 1 | 1 | 2.32 | -3.19 | High | Yes | No | Yes | No | No | No | No | -4.87 | 0 |
| **68** | 150.22 | 1 | 1 | 2.24 | -3.31 | High | Yes | No | Yes | No | No | No | No | -4.74 | 0 |
| **69** | 134.22 | 0 | 0 | 2.51 | -3.63 | Low | Yes | No | No | No | No | Yes | No | -4.21 | 1 |
| **70** | 136.23 | 0 | 0 | 2.89 | -3.05 | Low | Yes | No | No | No | No | No | No | -4.17 | 0 |
| **71** | 154.25 | 1 | 1 | 2.7 | -2.4 | High | Yes | No | No | No | No | No | No | -5.13 | 0 |
| **72** | 154.25 | 1 | 0 | 2.49 | -2.88 | High | Yes | No | No | No | No | No | No | -4.52 | 0 |
| **73** | 152.23 | 1 | 0 | 0 | -2.15 | High | Yes | No | No | No | No | No | No | -5.62 | 0 |
| **74** | 152.23 | 1 | 0 | 2.29 | -2.15 | High | Yes | No | No | No | No | No | No | -5.62 | 0 |
| **75** | 154.25 | 1 | 1 | 2.46 | -2.07 | High | Yes | No | No | No | No | No | No | -5.74 | 0 |
| **76** | 152.23 | 1 | 1 | 2.16 | -1.85 | High | Yes | No | No | No | No | No | No | -5.96 | 0 |
| **77** | 138.21 | 1 | 0 | 2.05 | -1.67 | High | Yes | No | No | No | No | No | No | -5.97 | 0 |
| **78** | 154.25 | 1 | 1 | 2.35 | -2.07 | High | Yes | No | No | No | No | No | No | -5.74 | 0 |
| **79** | 136.23 | 0 | 0 | 2.65 | -2.57 | Low | Yes | No | No | No | No | No | No | -4.94 | 1 |
| **80** | 150.22 | 1 | 0 | 2.16 | -2.06 | High | Yes | No | No | No | No | No | No | -5.61 | 0 |
| **81** | 154.25 | 1 | 1 | 2.29 | -2.51 | High | Yes | No | No | No | No | No | No | -5.31 | 0 |
| **82** | 152.23 | 1 | 0 | 2.12 | -2.16 | High | Yes | No | No | No | No | No | No | -5.67 | 0 |
| **83** | 136.23 | 0 | 0 | 2.58 | -3.34 | Low | Yes | No | No | No | Yes | No | No | -4.13 | 1 |
| **84** | 136.23 | 0 | 0 | 2.63 | -2.48 | Low | Yes | No | No | No | No | No | No | -5.11 | 1 |
| **85** | 136.23 | 0 | 0 | 2.59 | -3.31 | Low | Yes | No | No | No | Yes | No | No | -4.18 | 1 |
| **86** | 154.25 | 1 | 0 | 2.58 | -2.52 | High | Yes | No | No | No | No | No | No | -5.3 | 0 |
| **87** | 152.23 | 1 | 1 | 2.33 | -1.85 | High | Yes | No | No | No | No | No | No | -6.03 | 0 |
| **88** | 136.23 | 0 | 0 | 2.72 | -3.5 | Low | Yes | No | No | No | Yes | No | No | -3.89 | 0 |
| **89** | 154.25 | 1 | 1 | 2.51 | -2.78 | High | Yes | No | No | No | No | No | No | -4.93 | 0 |
| **90** | 154.25 | 1 | 1 | 2.51 | -2.87 | High | Yes | No | No | No | No | No | No | -4.83 | 0 |
| **91** | 152.23 | 1 | 1 | 2.5 | -2.68 | High | Yes | No | No | No | No | No | No | -5.01 | 0 |
| **92** | 150.22 | 1 | 0 | 2.27 | -2.41 | High | Yes | No | No | No | No | No | No | -5.29 | 0 |
| **93** | 136.23 | 0 | 0 | 2.73 | -3.45 | Low | Yes | No | No | No | No | No | No | -3.94 | 0 |
| **94** | 152.23 | 1 | 1 | 2.42 | -2.23 | High | Yes | No | No | No | No | No | No | -5.52 | 0 |
| **95** | 154.25 | 1 | 1 | 2.48 | -2.75 | High | Yes | No | No | No | No | No | No | -4.96 | 0 |
| **96** | 234.33 | 2 | 1 | 2.32 | -3.12 | High | Yes | No | No | No | No | No | No | -5.67 | 0 |
| **97** | 278.39 | 3 | 0 | 2.71 | -3.78 | High | Yes | No | No | No | Yes | No | No | -5.36 | 0 |
| **98** | 220.35 | 1 | 1 | 2.87 | -3.3 | High | Yes | No | No | Yes | Yes | No | No | -5.29 | 0 |
| **99** | 204.35 | 0 | 0 | 3.24 | -3.87 | Low | No | No | No | Yes | Yes | No | No | -4.44 | 1 |
| **100** | 276.37 | 3 | 0 | 2.95 | -3.61 | High | Yes | No | No | Yes | Yes | No | No | -5.52 | 0 |
| **101** | 218.33 | 1 | 0 | 2.72 | -3.37 | High | Yes | No | No | Yes | Yes | No | No | -5.18 | 0 |
| **102** | 218.33 | 1 | 0 | 2.78 | -3.68 | High | Yes | No | No | No | Yes | No | No | -4.83 | 0 |
| **103** | 220.35 | 1 | 0 | 2.93 | -3.84 | High | Yes | No | No | No | Yes | No | No | -4.68 | 0 |
| **104** | 234.33 | 2 | 0 | 2.6 | -3.38 | High | Yes | No | No | No | Yes | No | No | -5.38 | 0 |
| **105** | 204.35 | 0 | 0 | 3.38 | -3.76 | Low | No | No | No | Yes | Yes | No | No | -4.49 | 1 |
| **106** | 204.35 | 0 | 0 | 3.41 | -3.43 | Low | No | No | No | Yes | Yes | No | No | -4.85 | 1 |
| **107** | 222.37 | 1 | 1 | 3.12 | -3.26 | High | Yes | No | No | Yes | No | No | No | -5.29 | 0 |
| **108** | 204.35 | 0 | 0 | 3.29 | -4.36 | Low | No | No | No | Yes | Yes | No | No | -3.8 | 1 |
| **109** | 222.37 | 1 | 1 | 3.19 | -2.99 | High | Yes | No | No | No | No | No | No | -5.59 | 0 |
| **110** | 222.37 | 1 | 1 | 3.2 | -3.8 | High | Yes | No | No | No | Yes | No | No | -4.53 | 0 |
| **111** | 220.35 | 1 | 1 | 3.04 | -3.17 | High | Yes | No | No | Yes | No | No | No | -5.44 | 0 |
| **112** | 220.35 | 1 | 0 | 3.15 | -3.45 | High | Yes | No | No | Yes | Yes | No | No | -5.12 | 0 |
| **113** | 234.38 | 1 | 0 | 3.27 | -3.98 | High | Yes | No | No | No | No | No | No | -4.63 | 0 |
| **114** | 202.34 | 0 | 0 | 3.5 | -4.52 | Low | No | No | No | No | No | Yes | No | -3.71 | 1 |
| **115** | 204.35 | 0 | 0 | 3.29 | -3.97 | Low | No | No | No | No | Yes | No | No | -4.32 | 1 |
| **116** | 488.79 | 3 | 1 | 6.87 | -9.71 | Low | No | Yes | No | No | No | No | No | 0.22 | 1 |
| **117** | 460.73 | 3 | 1 | 6.09 | -8.99 | Low | No | No | No | Yes | No | No | No | -0.38 | 1 |
| **118** | 500.8 | 3 | 1 | 6.99 | -9.9 | Low | No | Yes | No | No | No | No | No | 0.27 | 2 |
| **119** | 472.74 | 3 | 1 | 6.53 | -9.17 | Low | No | No | No | Yes | No | No | No | -0.33 | 1 |

MW^1^: Molecular weigth; HBA^2^: Number of Hydrogen Bonding Acceptor; HBD^3^: Number of Hydrogen Bonding Donor; _i_LOGP^4^: -Log_10_ (Organic/Water); LogS^5^: Water Solubility Class (VS= Very Soluble, HS= Highly soluble, S= Soluble, MS= Moderately soluble, PS= Poorly soluble); GI^6^: Gastrointestinal Absorption; BBB^7^: blood-brain barrier; P-gp^8^: P-glycoprotein substrate; CYP_1A2_^9^: CYP_1A2_ inhibitor; CYP_2C19_^10^: CYP_2C19_ inhibitor; CYP_2C9_^11^: CYP_2C9_ inhibitor; CYP_2D6_^12^: CYP_2D6_ inhibitor; CYP_3A4_^13^: CYP_3A4_ inhibitor; LogK^14^: Log_10_ (K) representative of Skin permeation; Lipinski^15^: Violation of any Lipinski’s Rules.

**Table S4**. PAINS alerts for metabolites from *Buddleja globosa* (SwissADME predictions).

| **N°** | **Compound** | **PAINS** |
| --- | --- | --- |
| 1 | Luteolin | 1 alert: catechol_A |
| 2 | 6-hydroxyluteolin | 1 alert: catechol_A |
| 3 | Luteolin 7-*O*-glucoside | 1 alert: catechol_A |
| 4 | 6-Hydroxyluteolin-7-*O*-glucoside | 1 alert: catechol_A |
| 5 | Apigenin 7-*O*-glucoside | 0 alert |
| 6 | Rutin | 1 alert: catechol_A |
| 7 | Quercetin | 1 alert: catechol_A |
| 8 | Apigenin | 0 alert |
| 9 | Quercitrin | 1 alert: catechol_A |
| 10 | Chrysin | 0 alert |
| 11 | Quercetin 3-*O*-glucoside | 1 alert: catechol_A |
| 12 | Luteolin 4'-*O*-rutinoside | 0 alert |
| 13 | Robinin | 0 alert |
| 14 | 4'-*O*-Rutinoside of 7-*O*-methylated apigenin / Genkwanin 4′-*O*-rutinoside | 0 alert |
| 15 | 6-Methoxy-apigenin / Hispidulin | 0 alert |
| 16 | 7-*O*-Methylated apigenin / Genkwanin | 0 alert |
| 17 | Isorhamnetin glucosyl-rhamnoside | 0 alert |
| 18 | Acacetin-7-*O*-rutinoside / Linarin | 0 alert |
| 19 | Quercetin‑3‑*O*‑rutinoside | 1 alert: catechol_A |
| 20 | Scutellarein-7-*O*-glucoside | 1 alert: catechol_A |
| 21 | 3'-*O*-Rutinoside of 7-methylated flavone | 0 alert |
| 22 | Luteolin 6,8-di-C-glucoside succinate | 1 alert: catechol_A |
| 23 | Pinocembrine | 0 alert |
| 24 | Catechin | 1 alert: catechol_A |
| 25 | Epicatechin | 1 alert: catechol_A |
| 26 | Cyanidin | 1 alert: catechol_A |
| 27 | Malvidin | 0 alert |
| 28 | Benzoic acid | 0 alert |
| 29 | Salicylic acid | 0 alert |
| 30 | Syringic acid | 0 alert |
| 31 | Gallic acid | 1 alert: catechol_A |
| 32 | Caffeic acid | 1 alert: catechol_A |
| 33 | Ferulic acid | 0 alert |
| 34 | *p*-coumaric acid | 0 alert |
| 35 | Vanillin | 0 alert |
| 36 | Caffeoylshikimic acid | 1 alert: catechol_A |
| 37 | Chlorogenic acid | 1 alert: catechol_A |
| 38 | Cryptochlorogenic acid | 1 alert: catechol_A |
| 39 | Acteoside / Verbascoside | 1 alert: catechol_A |
| 40 | Echinacoside | 1 alert: catechol_A |
| 41 | Isoverbascoside | 1 alert: catechol_A |
| 42 | Forsythoside A | 1 alert: catechol_A |
| 43 | Campneoside I | 1 alert: catechol_A |
| 44 | Martynoside | 0 alert |
| 45 | Lipedoside A | 1 alert: catechol_A |
| 46 | Eukovoside | 1 alert: catechol_A |
| 47 | Forsythoside B | 1 alert: catechol_A |
| 48 | Angoroside A | 1 alert: catechol_A |
| 49 | Aucubin | 0 alert |
| 50 | *p*-methoxycinnamoyl-aucubin | 0 alert |
| 51 | Catalpol | 0 alert |
| 52 | *p*-methoxycinnamoyl-catalpol | 0 alert |
| 53 | Catalposide | 0 alert |
| 54 | 6-*O*-methylcatalpol | 0 alert |
| 55 | Buddlejone | 0 alert |
| 56 | Desoxybuddlejone | 0 alert |
| 57 | Maytenone | 0 alert |
| 58 | Lupeol | 0 alert |
| 59 | α-amyrin | 0 alert |
| 60 | β-amyrin | 0 alert |
| 61 | β-sitosterol | 0 alert |
| 62 | β-sitosterol glucoside | 0 alert |
| 63 | Stigmasterol | 0 alert |
| 64 | Campesterol | 0 alert |
| 65 | Stigmastanol | 0 alert |
| 66 | Ergosterol | 0 alert |
| 67 | Thymol | 0 alert |
| 68 | Carvacrol | 0 alert |
| 69 | *p*-cymene | 0 alert |
| 70 | Myrcene | 0 alert |
| 71 | Linalool | 0 alert |
| 72 | Citronellal | 0 alert |
| 73 | *cis-*Thujone | 0 alert |
| 74 | *trans*-Thujone | 0 alert |
| 75 | Thujanol | 0 alert |
| 76 | *trans*-Sabinol | 0 alert |
| 77 | Sabina ketone | 0 alert |
| 78 | *cis*-Sabinene hydrate | 0 alert |
| 79 | Sabinene | 0 alert |
| 80 | Thuj-3-en-10-al | 0 alert |
| 81 | Borneol | 0 alert |
| 82 | Camphor | 0 alert |
| 83 | Camphene | 0 alert |
| 84 | α-Pinene | 0 alert |
| 85 | β-Pinene | 0 alert |
| 86 | 1,8-Cineole | 0 alert |
| 87 | *cis*-Chrysanthenol | 0 alert |
| 88 | Limonene | 0 alert |
| 89 | Terpinen-4-ol | 0 alert |
| 90 | α-Terpineol | 0 alert |
| 91 | *trans*-Carveol | 0 alert |
| 92 | Carvone | 0 alert |
| 93 | γ-Terpinene | 0 alert |
| 94 | *cis*-*p*-Mentha2,8-dien-1-ol | 0 alert |
| 95 | Dihydro carveol | 0 alert |
| 96 | Buddledin B | 0 alert |
| 97 | Dihydrobuddledin A | 0 alert |
| 98 | Caryophylla4(12),8(13)-dien5-ol | 0 alert |
| 99 | (E)-Caryophyllene | 0 alert |
| 100 | Buddledin A | 0 alert |
| 101 | Buddledin C | 0 alert |
| 102 | Zerumbone | 0 alert |
| 103 | Buddledone A | 0 alert |
| 104 | Buddledone B | 0 alert |
| 105 | γ-Cadinene | 0 alert |
| 106 | δ-Cadinene | 0 alert |
| 107 | Murrolol | 0 alert |
| 108 | γ-Gurjunene | 0 alert |
| 109 | Bulnesol | 0 alert |
| 110 | Elemol | 0 alert |
| 111 | Spathulenol | 0 alert |
| 112 | Caryophyllene oxide | 0 alert |
| 113 | β-Cedrene epoxide | 0 alert |
| 114 | α-Curcumene | 0 alert |
| 115 | α-Humulene | 0 alert |
| 116 | 2-(4-hydroxyphenyl)ethanol ester of lignoceric acid | 0 alert |
| 117 | Ester of 2-(4-hydroxyphenyl) ethanol ester of docosanolc acid / 2-(4-hydroxyphenyl)ethyl dodecanoate | 0 alert |
| 118 | Ester of lignoceric acid and 4-hydroxycinnamyl alcohol / Lignoceryl 4-hydroxycinnamyl alcohol | 0 alert |
| 119 | 4-hydroxy-cinnamyl alcohol and docosanoic acid | 0 alert |

**Table S5**. Hierarchical classification, methodological quality assessment, and limitations of the included studies on *B. globosa.*

| **Document (No.)** | **Authors** | **Study type / Biological model** | **Intervention evaluated** | **Level of evidence** | **Quality / Risk of bias** | **Critical limitation** |
| --- | --- | --- | --- | --- | --- | --- |
| 1 | Suwalsky et al., 2017 | *In vitro* / Human erythrocytes and model membranes (DMPC/DMPE) | Aqueous leaf extract of *B. globosa* | **Level V**  (Crude extract *in vitro*) | Moderate | Concentrations used (µM to mM) are not achievable in human plasma. |
| 2 | Backhouse et al., 2008a | *In vivo* (rodents) / Analgesia (writhing, tail flick), anti-inflammatory (ear and paw edema) assays | Sequential extracts (hexane, DCM, methanol) and isolated compounds (amyrins, sterols, verbascoside) | **Level III**  (*In vivo* - animal) | High | Doses used in mice (e.g., 600 mg/kg) are very high for human extrapolation; no associated acute toxicity studies. |
| 3 | Houghton & Hikino, 1989 | *In vitro* / Cultured hepatocytes (CCl_4_, galactosamine-induced toxicity) | Aqueous extracts of *B.globosa* leaves and isolated compounds (iridoids, phenylpropanoids, flavonoids) | **Level IV**  (Mechanistic - isolated compound) | Moderate | *In vitro* assay in isolated cells; does not reflect complete hepatic metabolism or oral bioavailability. |
| 4 | Carmona et al., 2016 | *In vivo* (*Drosophila melanogaste*r) / Wing-spot assay (SMART) | Aqueous leaf extract of *B. globosa* | **Level III**  (*In vivo* - animal) | Moderate | Drosophila model; DNA repair mechanisms and metabolism differ from mammals. |
| 5 | Backhouse et al., 2008b | *In vivo* (rodents) / Nociception assays (formalin, tail flick, writhing) with pharmacological antagonists | Methanolic and ethanolic extracts (seasonal) and pure compounds (verbascoside, luteolin-7-*O*-glucoside) | **Level III**  (*In vivo* - animal) | High | Seasonal variation in verbascoside concentration affects result reproducibility. |
| 6 | Zamorano-Aguilar et al., 2020 | *In vitro* / CHO-K1 cell line (Chinese hamster ovary) | Flower extracts (aqueous) | **Level V**  (Crude extract *in vitro*) | Low | Lack of evaluation in inflammatory or wound healing models; the extract is cytotoxic to healthy cells at high concentrations. |
| 7 | Vera et al., 2023 | Green synthesis / ZnO nanoparticles | *B. globosa* leaf extract as reducing and stabilizing agent | **Level V**  (Materials chemistry study) | Moderate | The study does not evaluate the pharmacological activity of the NPs; it only characterizes the synthesis process. |
| 8 | Gastaldi et al., 2016 | *In vitro* / Chemical assays (DPPH) | Infusions, tinctures, and essential oils of native species | **Level V**  (Chemical *in vitro* study) | Low | Does not evaluate biological effects on cells or animals; only a chemical measurement. |
| 9 | Alarcón et al., 2008 | *In vitro* / Chemical assays (ORAC with different probes) | Infusions of *B. globosa* and other herbs | **Level V**  (Methodological chemical study) | Low | The study is methodological; no biological evaluation. ORAC results are not directly translatable to in vivo effects. |
| 10 | Speisky et al., 2006 | *In vitro* / Chemical assays (TEAC, HClO and peroxynitrite scavenging) | Herb infusions (including *B. globosa*) | **Level V**  (Chemical *in vitro* study) | Low | No cellular models; in vitro radical-scavenging capacity does not guarantee *in vivo* effects. |
| 11 | Letelier et al., 2017 | Clinical trial / Women with urinary tract infection (*n* = 56) | Nitrofurantoin + Standardized BG-126 extract (vs. placebo) | **Level I**  (RCT / Human) | High | Small sample size (*n* = 56); only evaluates gastrointestinal adverse effects, not antimicrobial efficacy (although reported). |
| 12 | Fuentes et al., 2018 | *In vitro* / Human platelets (aggregation, flow cytometry, Western blot) | Ethanolic leaf extract of *B. globosa* | **Level IV**  (Mechanistic in vitro - extract) | Moderate | Lack of *in vivo* studies to confirm an antithrombotic effect; the extract is complex, and the responsible compound was not isolated. |
| 13 | Ceriani et al., 2026 | *In vivo* (rats) / Chronic ischemic wound model | Gelatin-chitosan-hyaluronic acid scaffold with *B. globosa* extract | **Level III**  (*In vivo* - animal) | High | No acceleration of wound closure was observed (contraction). Improvement was in tissue quality, not speed. |
| 14 | Quintero-Pertuz et al., 2025 | *In vitro* / *Trypanosoma cruzi* (trypomastigotes) and Vero cells | Methanolic leaf extract and iridoid-enriched fraction (BG500) | **Level IV**  (Mechanistic - extract and fraction) | Moderate | Moderate activity (IC50 280 µg/mL) compared to nifurtimox (5 µg/mL); *in vivo* studies are needed to confirm efficacy. |
| 15 | Houghton et al., 1996 | Chemical isolation / Roots of *B. albiflora* and *B. globosa* | Isolation of buddlejone (diterpene) | **Level V**  (Natural products chemistry) | Very low | No biological activity is evaluated. It is a purely chemical study. |
| 16 | Fischer et al., 2011 | Agronomic study / Habitat characterization and germination | Accessions of *B. globosa* from different provenances | **Level V**  (Cultivation and domestication study) | N/A | Does not evaluate any biological activity. |
| 17 | Pellegrini et al., 2017 | *In vitro* / *Paenibacillus larvae* (bacteria) | Essential oils of several species (including *B. globosa*) | **Level V**  (Crude extract *in vitro*) | Moderate | Very low yield of *B. globosa* essential oil; the EO is complex and variable; activity is correlated with unspecified volatile compounds. |
| 18 | Fernández et al., 2018 | Observational study / Patients with IBD (*n* = 200) | Use of complementary and alternative medicine (including *B. globosa* as an herbal medicine) | **Level II**  (Human - observational) | Moderate | Efficacy is not evaluated; only prevalence of use. The quality of the consumed product is not controlled. |
| 19 | Letelier et al., 2008 | *In vitro* / Rat liver microsomes | *B. globosa* extract and DPPH/oxygen free radicals | **Level V**  (Crude extract *in vitro*) | Moderate | Subcellular study; does not reflect the complexity of a living cell; crude extract may have artifacts. |
| 20 | Vogel et al., 2010 | Agronomic study / Variation of polyphenols and antioxidant activity in BG leaves | Different provenances, harvest times, irrigation, and drying methods | **Level V**  (Raw material quality study) | N/A | Does not evaluate biological activities beyond chemistry. It is a study to optimize cultivation and harvesting. |
| 21 | Mensah et al., 2001 | *In vitro* / Human dermal fibroblasts (proliferation and antioxidant protection) | Aqueous leaf extract of *B. globosa* and isolated compounds (verbascoside, linarin, luteolin) | **Level IV**  (Mechanistic - isolated compounds) | High | The extract slightly stimulates proliferation, but it is not statistically significant; at high concentrations, it is cytotoxic. |
| 22 | Aboshama, 2011 | *In vitro* culture / Plant propagation | Different culture media and growth regulators for *B. globosa* | **Level V**  (Biotechnological study) | N/A | Does not evaluate any biological or pharmacological activity. It is a micropropagation study. |
| 23 | Hermosilla et al., 2025 | *In vitro* / 3T3-L1 fibroblasts and antimicrobial assays (*S. aureus, P. aeruginosa, C. albicans*) | PCL fibers with extracts of *G. tinctoria* and *B. globosa* (M1) | **Level V**  (Crude extract *in vitro*) | Moderate | The *B. globosa* (M1) extract individually did not show potent antibacterial activity; the antifungal activity observed is modest. |
| 24 | Parada et al., 2014 | *In vivo* (rats) / Estrous cycle and estrogen receptor binding | Ethanolic leaf extract of BG standardized in verbascoside (EMATst) | **Level III**  (*In vivo* - animal) | High | Effect observed only at very high doses (10x); relevance to humans is uncertain; verbascoside shows low affinity for the receptor. |
| 25 | Campos & Lissi, 1995 | *In vitro* / Chemical assays (ABTS) | Herb infusions (including *B. globosa*) | **Level V**  (Methodological chemical study) | Very low | There is no biological evaluation; it is an analytical chemistry work describing a method. |
| 26 | Araya et al., 2022 | *In vitro* / *Pseudomonas aeruginosa* (proliferation and biofilm) | BG-126 extract and spray-dried formulations (BG-SD, BG-SD PVP, BG-SD SP) | **Level V**  (Crude extract *in vitro*) | Moderate | The crude extract is not standardized beyond catechin; the activity is modest compared to conventional antibiotics. |
| 27 | Carmona et al., 2017 | Green synthesis / Silver nanoparticles (AgNPs) | *B. globosa* leaf extract as a reducing agent | **Level V**  (Materials chemistry study) | Moderate | The study focuses on NP synthesis, not on their antimicrobial or biological activity. |
| 28 | Letelier et al., 2017b | *In vitro* / Rat liver microsomes | Extracts of several plants (including BG) and drugs (nitrofurantoin, nifurtimox, acetaminophen) | **Level V**  (Crude extract *in vitro*) | Moderate | Subcellular study; does not evaluate pharmacokinetic interactions in the whole organism. |
| 29 | Debenedetti et al., 2002 | *In vitro* / *Plasmodium falciparum* (chloroquine-resistant strain) | Aqueous and methanolic extracts of Argentine plants | **Level V**  (Crude extract *in vitro*) | Moderate | The active compounds are not identified; the activity is lower than the positive control. |
| 30 | Houghton et al., 2005 | Review / *In vitro* tests for wound healing | *B. globosa* as an example of a wound healing study | **Level V**  (Methodology review) | N/A | It is not an experimental study; it is a review that mentions *B. globosa* as an example. |
| 31 | Mensah et al., 2001 | *In vitro* / Human fibroblasts | Aqueous leaf extract of BG | **Level IV**  (Mechanistic - extract) | Moderate | It is a conference abstract (Supplement). Full data are not available. Preliminary results; no statistical details are provided for the collagen lattice contraction assay***.*** |
| 32 | Arismendi & Böhm, 2011 | Ecological study / Lettuce (*Lactuca sativa*) infected with M. hapla | Dry leaves of BG and other species incorporated into the substrate | **Level V**  (Plant-nematode interaction study) | Low | Does not evaluate pharmacological activity in mammals. It is an agronomic study on nematode control. |
| 33 | López-Alarcón et al., 2009 | *In vitro* / Chemical assays (ORAC with alizarin red vs. fluorescein) | Herb infusions (including *B. globosa*) | **Level V**  (Methodological chemical study) | Very low | There is no biological evaluation; it is a purely methodological study. |
| 34 | Pardo et al., 1993 | *In vitro* / *Staphylococcus aureus* and *Escherichia coli* | Ethanolic leaf extract of BG and isolated compound (verbascoside) | **Level IV**  (Mechanistic - isolated compound) | Moderate | Only evaluates antibacterial activity in two strains; does not evaluate cytotoxicity or *in vivo* activity. |
| 35 | Mensah et al., 2000 | *In vitro* / Dermatophyte fungi (*Trichophyton*, *Epidermophyton*) | Extracts and isolated compounds (buddledins, maytenone, buddlejone) | **Level IV**  (Mechanistic - isolated compounds) | High | The toxicity of the compounds is not evaluated; only their *in vitro* antifungal activity. |
| 36 | Vogel et al., 2011 | Agronomic study / Morphological characterization and yield of BG | Wild and cultivated accessions of BG | **Level V**  (Domestication study) | N/A | Does not evaluate any pharmacological activity. It is a study for raw material production. |
| 37 | Böhm et al., 2009 | Ecological study / *Meloidogyne hapla* (nematode) on lettuce | Dry leaves of BG and other species as organic amendment | **Level V**  (Plant-nematode interaction study) | Low | Does not evaluate pharmacology in mammals; it is an agronomic study. |
| 38 | Liao et al., 1999 | *In vitro* / Rat leukocytes (eicosanoids) and compound isolation | Extracts of several *Buddleja* species (including BG) and isolated compounds | **Level IV**  (Mechanistic - isolated compounds) | High | Studies in rat leukocytes; lack of *in vivo* evaluation to confirm anti-inflammatory activity. |
| 39 | Pardo et al., 1997 | *In vitro* / *Staphylococcus aureus* | Ethanolic extract of BG and isolated compound (angoroside A) | **Level IV**  (Mechanistic - isolated compound) | Moderate | Only evaluates antibacterial activity *in vitro*; cytotoxicity is not evaluated. |
| 40 | Gastaldi et al., 2015 | *In vitro* / Chemical assays (DPPH, BCB), *Artemia salina* toxicity, and antiproliferative activity in T84 and HTR-8 cells | Infusions of native Patagonian plants (including BG) | **Level V**  (Crude extract *in vitro*) | Moderate | The antiproliferative activity in T84 cells is moderate (EC_50_ 1.37 mg/mL); the mechanism of action is not evaluated. |
| 41 | Ceriani et al., 2026 | *In vitro* / *In vivo* (rats) / Wounds infected with dual-species biofilm (*P. aeruginosa* and *S. aureus*) | Polymeric scaffolds (gelatin-chitosan-HA) with BG2, BG3, and BG4 | **Level III**  (*In vivo* - animal) | High | Bacterial load was measured only at the end of treatment; the model uses healthy rats, not diabetic or immunocompromised**.** |
| 42 | Houghton, 1989 | Chemical isolation / Stem bark of BG | Isolation of phenolic fatty acid esters (suberin) | **Level V**  (Natural products chemistry) | Very low | No biological activity is evaluated. |
| 43 | Marín et al., 1979 | Chemical isolation / Flowers of *B. globosa* | Isolation of flavonoids (acacetin-7-*O*-rutinoside, apigenin-7-*O*-glucoside) and lupeol | **Level V**  (Natural products chemistry) | Very low | No biological activity is evaluated. It is a purely chemical study. |
| 44 | Torres-Vega et al., 2021 | Analytical chemistry / Development of extraction and purification methods | *B. globosa* leaf extracts with NADES and CPC | **Level V**  (Analytical chemistry study) | Moderate | No pharmacological activity is evaluated. This study focuses on extraction and purification optimization. |
| 45 | Hernández et al., 2025 | *In vitro* / Chemical assays (antioxidants), antibacterial (*E. coli, S. aureus*), and cytotoxicity in human cells (HCT116) | Ethanolic leaf extracts of *B. globosa* and other native species | **Level V**  (Crude extract *in vitro*) | Moderate | The antibacterial activity of *B. globosa* was moderate (MIC 50 mg/mL) compared to other species; the mechanism of action is not evaluated. |
| 46 | Wilckens et al., 2013 | Agronomic study / Sustainable management of *B. globosa* | Comparison of wild and cultivated plants; different pruning and irrigation methods | **Level V**  (Domestication and management study) | N/A | Does not evaluate any biological activity. It is a study of sustainable raw-material production. |
| 47 | Ceriani et al., 2025 | *In vitro* / Antimicrobial assays (*S. aureus, P. aeruginosa*) and biocompatibility (human fibroblasts) | Polymeric scaffolds (gelatin-chitosan-HA) with BG-126 extract (Box-Behnken design) | **Level IV**  (Mechanistic - extract in a matrix) | High | Lack of *in vivo* studies; only evaluates antimicrobial and compatibility properties in cell cultures. |
| 48 | Parichanon et al., 2025 | *In vitro* / Rhyzoptera dominica (grain beetle) | Essential oil of *B. globosa* and 29 other plants | **Level V**  (Crude extract *in vitro* - ecology) | Moderate | Does not evaluate pharmacological activity in mammals; it is a pest control study. The yield of *B. globosa* EO is low. |
| 49 | Rubio, 2011 | Clinical trial / Patients with palmar-plantar erythrodysesthesia (PPE) (n=121) and radioepithelitis (n=78) | Lotion and gel with standardized BG extract (EMAT-1) vs. placebo (double-blind) | **Level I**  (RCT / Human) | High | Moderate sample size (121 and 78); single-country study; mechanism of action in humans was not evaluated. |
| 50 | Castro-Saavedra et al., 2016 | *In vitro* / Chemical assays (ABTS, DPPH, FRAP, CUPRAC, TPC, TFC), 15-LOX inhibition, and cytotoxicity in MCF-7 and MCF-10A cell lines | Ethanolic leaf extracts of 19 Chilean species (including *B. globosa*) | **Level V**  (Crude extract *in vitro*) | Low | Conference abstract with limited methodological detail. Available data for *B. globosa* include total phenolic and flavonoid content, rutin and polyphenol quantification, moderate antioxidant activity, and no 15-LOX inhibition or cytotoxicity. |
